# Synthesis and Preliminary Cytotoxicity Evaluation of 3-Lup-20(29)-Ene-3β,28-Diol Glycoconjugates Containing a Succinic Linker and a 1,2,3-Triazole Ring

**DOI:** 10.3390/cancers17111737

**Published:** 2025-05-22

**Authors:** Julia Szreder, Klaudia Woźniak, Karol Erfurt, Mirosława Grymel, Gabriela Pastuch-Gawołek

**Affiliations:** 1 Department of Organic Chemistry, Bioorganic Chemistry and Biotechnology, Silesian University of Technology, B. Krzywoustego 4, 44-100 Gliwice, Poland; julia.szreder@polsl.pl (J.S.);; 2 Department of Chemical Organic Technology and Petrochemistry, Silesian University of Technology, B. Krzywoustego 4, 44-100 Gliwice, Poland; karol.erfurt@polsl.pl; 3 Biotechnology Center, Silesian University of Technology, B. Krzywoustego 8, 44-100 Gliwice, Poland

**Keywords:** betulin glycoconjugates, click chemistry, cytotoxicity

## Abstract

**Background**: 3-Lup-20(29)-ene-3β,28-diol (betulin, BN) is a natural bioactive compound with significant synthetic and pharmacological potential. A growing body of research highlights the increasing interest in BN and its derivatives, driven by their broad biological activities (anticancer, antibacterial, anti-inflammatory, antiretroviral). However, poor bioavailability and low intracellular accumulation limit its pharmaceutical application. **Methods**: A promising strategy to enhance BN’s therapeutic potential is glycoconjugation. This approach improves drug bioavailability, solubility, and selectivity, particularly in cancer therapy, by leveraging cancer cells’ heightened glucose demand and overexpression of glucose transporters. Incorporating an *N*-heterocyclic linker, such as a 1,2,3-triazole ring, further enhances biological activity. **Results**: We developed an efficient method for modifying the betulin backbone at position C28 with sugar units via a (CO)CH_2_CH_2_COOH linker, based on CuAAC, yielding ten new betulin glycoconjugates with good yields and purity confirmed by spectroscopic analysis (NMR, HRMS). The potential for inhibition of cancer cell proliferation (HCT-116 human colorectal carcinoma cell line and MCF-7 human breast cancer cell line) and cytotoxicity toward normal human dermal fibroblasts (NHDF-Neo) was assessed. **Conclusions**: The obtained glycoconjugates exhibited higher activity against MCF-7, indicating the selectivity of their action. The development of glycoconjugates based on increased glucose demand and overexpression of its transporters could be an interesting strategy for acquiring anticancer agents, combining innovative chemical solutions with biological complexity. Such an approach may be crucial in the effective fight against cancer diseases.

## Simple Summary

3-Lup-20(29)-ene-3β,28-diol (betulin, BN) is a natural bioactive compound with broad biological activities, especially anticancer, antibacterial, anti-inflammatory, and antiretroviral. However, poor bioavailability and low intracellular accumulation limit its pharmaceutical application. A promising strategy to enhance BN’s therapeutic potential is glycoconjugation. We developed an efficient method for modifying the betulin backbone at position C28 with sugar units via a (CO)CH_2_CH_2_COOH linker, based on CuAAC, yielding ten new betulin glycoconjugates with good yields and purity confirmed by spectroscopic analysis (NMR, HRMS).

## 1. Introduction

**Figure 1:**
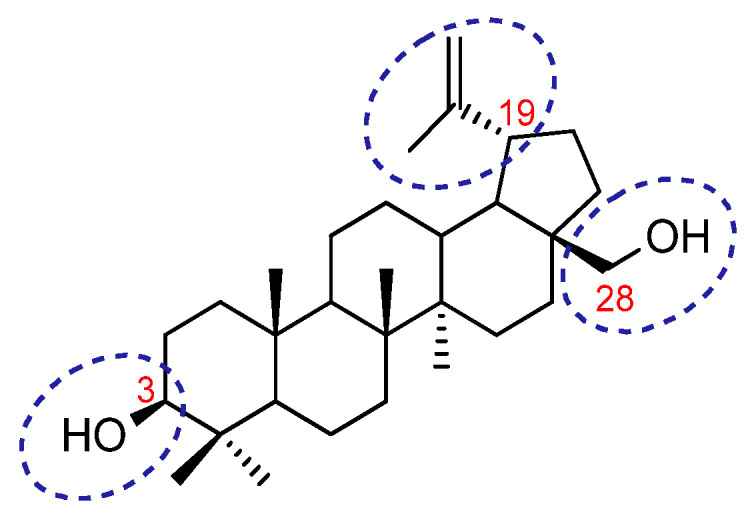
Chemical structure of betulin with indication of items for possible derivatization.

3-Lup-20(29)-ene-3β,28-diol, commonly known as betulin (BN), is an example of a natural bioactive substance with significant synthetic and pharmacological potential (Figure 1). Scientific articles and patents available in various databases highlight researchers’ growing interest in betulin. Its abundant availability, low cost, and structurally versatile parent framework make betulin an excellent candidate for the design of new derivatives with improved pharmacokinetic properties, particularly enhanced bioavailability [1,2,3]. Despite its wide range of biological activity, betulin has limitations, such as poor solubility and bioavailability. The parent skeleton therefore requires structural modifications to release its full therapeutic potential.

Therefore, research in this area is justified, allowing for the expansion of the library of BN derivatives, and enhancing the information on the structure-biological activity correlation.

One promising approach to improving the properties of bioactive substances is glycoconjugation, which involves attaching sugar units to the biologically active backbone. The sugar moiety can be linked to the active compound by forming a glycosidic bond or using various types of linkers. The linker may also contain structural elements important from the point of view of biological activity [4]. This strategy offers several advantages, including the ability to improve water solubility and bioavailability while improving selectivity toward specific molecular targets. Sugar moieties play a crucial role in directing glycoconjugates of biologically active compounds toward specific receptors or transporters that are overexpressed in cancer cells. This targeted delivery mechanism reduces off-target effects and increases the therapeutic efficacy of the compound [5]. The phenomenon that allows us to understand the role of sugars in targeted drug delivery in anticancer therapy is the Warburg effect. It explains that cancer cells have a different metabolism than healthy cells. Even with sufficient oxygen supply, anaerobic respiration associated with lactic acid fermentation dominates, as a result of which cancer cells convert glucose mainly into lactate [6,7]. The effect of this different metabolism is an increased demand for glucose in cancer cells and its enhanced transport across the cell membrane, which results in GLUT transporters overexpression [8,9,10]. The Warburg effect is particularly visible in the cases of colorectal [9,11], breast [12], lung cancers [13], or glioblastoma [14].

The glycoconjugation strategy has been used to modify the properties of anticancer drugs such as ifosfamide [15], gemcitabine [16], platinum derivatives [17], doxorubicin [18], or paclitaxel [19].

In the structure of many pharmaceuticals, both those already approved for use and those still under evaluation, an *N*-heterocyclic fragment is often present, and its presence has a significant impact on the biological activity [20,21]. Derivatives containing 1,2,3-triazole ring are of particular interest in drug design [22]. Such compounds have been widely studied for their antibacterial, antiviral, anti-inflammatory, anticancer, and antimicrobial activity [23]. Examples of drugs with a 1,2,3-triazole system in their structure include tazobactam, a β-lactam antibiotic [24], and savolitinib, a receptor tyrosine kinase (c-Met) inhibitor, approved in China for the treatment of selected types of cancer [25].

Based on this observation, it seems advantageous to connect the sugar with the BN backbone via a linker containing an *N*-heterocyclic system. This is possible due to the use of 1,3-dipolar azido-alkyne cycloaddition catalyzed by copper (CuAAC), which allows for a simple and efficient connection of the sugar and the bioactive compound via the 1,2,3-triazole ring [23].

**Figure 2:**
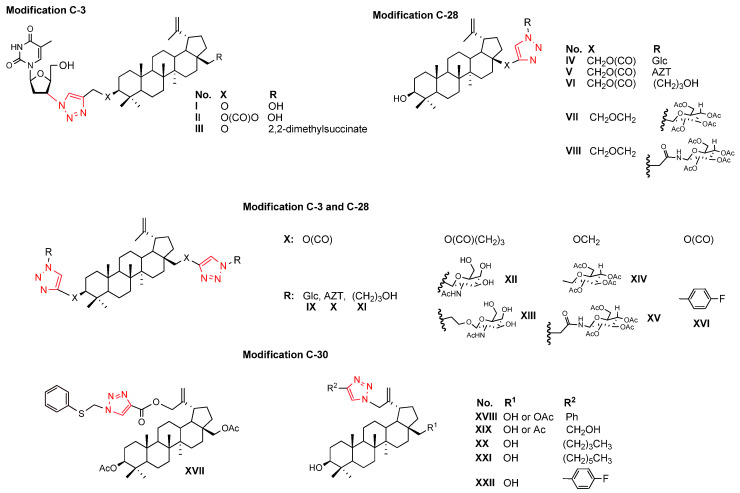
Selected structural modifications of betulin skeleton at positions C3, C28, and C30 via CuAAC method [26,27,28,29,30,31,32].

A variety of triazole derivatives of betulin, including glycoconjugates, have been described in the scientific literature and evaluated for their biological activities. In vitro studies have revealed that some of the obtained compounds show promising antiviral, anticancer (cytotoxic and antiproliferative), and antimicrobial (antibacterial and antifungal) properties, with a specific activity profile depending on the substituents introduced and the compound’s structure. Structural modifications of the BN backbone using click chemistry reactions (CuAAC) can target the positions C3, C28, and C30, as illustrated in Figure 2.

**Figure 3:**
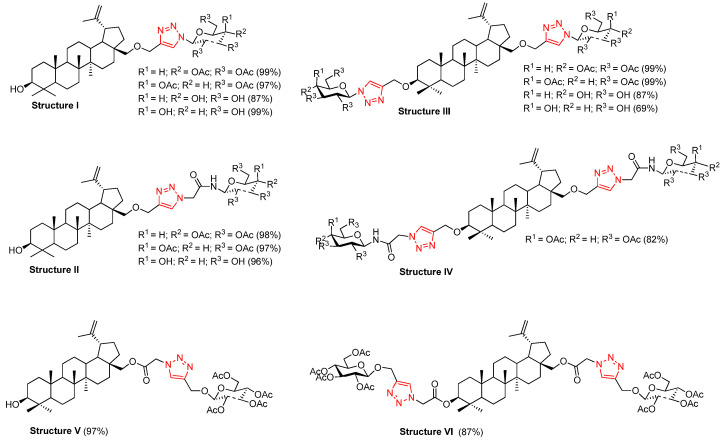
Betulin glycoconjugates with a 1,2,3-triazole linker at the C28 position or the C3 and C28 positions [28].

In our previous study, we performed modifications of the betulin backbone, involving the introduction of a sugar unit via a 1,2,3-triazole ring at C3 and/or C28 positions of BN (mono- or disubstituted glycoconjugates, Figure 3). Unfortunately, cytotoxicity assay results of these compounds turned out to be unsatisfactory. Furthermore, 3,28-*O*,*O*’-*bis*(2-(4-(hydroxymethyl-1H-1,2,3-triazol-1-yl)acetyl)betulin, modified only with a triazole system, demonstrated unexpectedly high in vitro activity against the MCF-7 cell line. However, it also showed toxicity against healthy cells, which is a serious limitation to its therapeutic potential. This observation confirmed our belief that the presence of the sugar moiety is necessary to improve selectivity [28].

**Figure 4:**
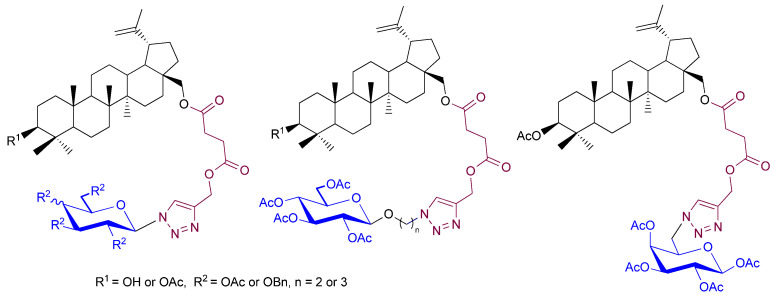
Structures of a new type of BN glycoconjugates.

In this work, we proposed new BN glycoconjugates (Figure 4), considering that an important element is the linker structure. Under standard physiological conditions, such a linker should exhibit sufficient stability and controlled lability, to allow the glycoconjugate to reach its target without premature degradation. Therefore, as a stable and biocompatible linker element between pharmacophores, we chose a triazole system, which presents hydrolysis resistance and adequate stability in biological systems (amide bond mimetism). In addition, the presence of a triazole ring may improve the solubility of glycoconjugates in water, which is important for their bioavailability and potential for in vivo administration.

The solution to the problem of structure stiffening could be the introduction of a fragment derived from succinic acid into the structure of betulin glycoconjugates. It should not only make the entire molecule more flexible, but also strengthen the chelating properties of metal ions by the designed glycoconjugates [33]. It is noteworthy that cancer cell growth and their metastasis are strongly related to copper ions uptake [34]. Therefore, it seems that metal chelating agents may be a promising strategy for controlling copper levels in the body, and consequently may be one of the components of anticancer therapy [35,36,37]. An additional advantage of such a solution is the combination of the betulin skeleton and the sugar ligand with the linker by an ester bond, which should undergo hydrolysis after reaching the target site, thus allowing the release of the drug in the cell.

## 2. Materials and Methods

### 2.1. General Experimental Informations

All compounds were obtained according to general principles of organic synthesis. The crude products were purified by column chromatography, and the structure and purity of the obtained compounds were confirmed by spectroscopic analysis using NMR (nuclear magnetic resonance), HRMS (high-resolution mass spectrometry), and determination of the values of optical rotations. All chemical reagents used in the synthesis were purchased from ACROS Organics (Geel, Belgium) or Avantor (Gliwice, Poland) and were not additionally purified or dried before use in the synthesis. Synthesis descriptions, spectra, and their analysis are included in the Supplementary Materials (Schemes S1–S3).

### 2.2. Biological Studies

For biological studies three different cell lines were used: the NHDF-Neo cell line (Lonza, Poland), the HCT-116 cell line (American Type Culture Collection, ATCC, Manassas, VA, USA), and the MCF-7 cell line (purchased by the Maria Sklodowska-Curie Memorial Cancer Center and Institute of Oncology, Gliwice, Poland and gifted for our research). Cells were cultured in 96-well plates (Sarstedt, Nümbrecht, Germany) using RPMI 1640 or DMEM/F12 media (100 µL per well) supplemented with fetal bovine serum (FBS, EURx, Gdańsk, Poland), and with Antibiotic–Antimycotic Solution (Avantor, Gliwice, Poland). Cells were seeded and incubated for 24 h prior to treatment: HCT-116 cells at a density of either 1 × 10^4^ or 7.5 × 10^3^ cells per well depending on incubation time, NHDF-Neo cells at 1 × 10^4^ per well, and MCF-7 at 5 × 10^3^ cells per well.

Stock solutions of the tested compounds were prepared in DMSO and stored under appropriate conditions. Immediately prior to the experiment, these solutions were diluted to the desired concentrations using a growth medium. In no dilution was the DMSO content higher than 1% *v*/*v*. The resulting solutions were then used to treat cells (100 µL per well). The studies were carried out in the concentration range from 25 µM to 200 µM of the tested compound, with incubation times varying from 24 to 72 h, depending on the specific cell type. In parallel, the influence of copper ions on cellular proliferation was investigated. In this case, the procedure was analogous to the previously described one, with the difference that glycoconjugates were added in the culture medium with the addition of CuCl_2_. The final concentration of CuCl_2_ was 20 µM in each well. Cytotoxicity studies were also carried out on an example of an anticancer drug, doxorubicin, against all tested cell lines. For determination of cell viability, the MTT assay (3-[4,5-dimethylthiazol-2-yl]-2,5-diphenyltetrazolium bromide; ACROS Organics) was applied following the manufacturer’s protocol. Absorbance of the formazan, formed during the test and dissolved in 100 µL of DMSO, was measured spectrophotometrically at 570 nm using a plate reader (Epoch, Bio-Tek, Winooski, VT, USA).

The measured absorbance was analyzed to assess proliferation, where tested samples were compared with the untreated controls (100%). The results from MTT were presented as the mean of three experiments (biological replicates). For each experimental condition within a replicate, four technical repetitions were performed. Technical repetitions were averaged to obtain a single value per replicate. Statistical analyses were conducted using means of the biological replicates. The percentage of viable cells and the standard deviation (SD) were calculated in Excel (Microsoft Office 365, A3 for faculty), using the STDEV.S formula, which determines the standard deviation for a sample set. The IC_50_ values were determined by generating dose-response curves using *GraphPad Prism software version 10.2.3*. The results are expressed as the average value ± standard deviation (SD). To evaluate statistical significance, two-way analysis of variance (ANOVA) with replication was performed to assess the effects of compound type and concentration on cell proliferation. When significant main effects were observed (*p* < 0.05), pairwise comparisons between betulin (BN) and each analogue at corresponding concentrations were conducted using two-tailed unpaired *t*-tests. Bonferroni correction was applied to account for multiple comparisons, and adjusted *p*-values < 0.05 were considered statistically significant. All statistical tests were performed in Excel.

### 2.3. In Silico Analysis

Molecular descriptors (logP, TPSA, molecular weight, hydrogen bond acceptors and hydrogen bond donors) of BN glycoconjugates were estimated using the *Molinspiration Cheminformatics 2025* platform (http://www.molinspiration.com, accessed on 8 February 2025). *SMILES (Simplified Molecular Input Line Entry System*) codes were generated using *ChemBioDraw Ultra version 12.0* (*Cambridge Software*) based on structural formulas.

## 3. Results and Discussion

**Scheme 1:**
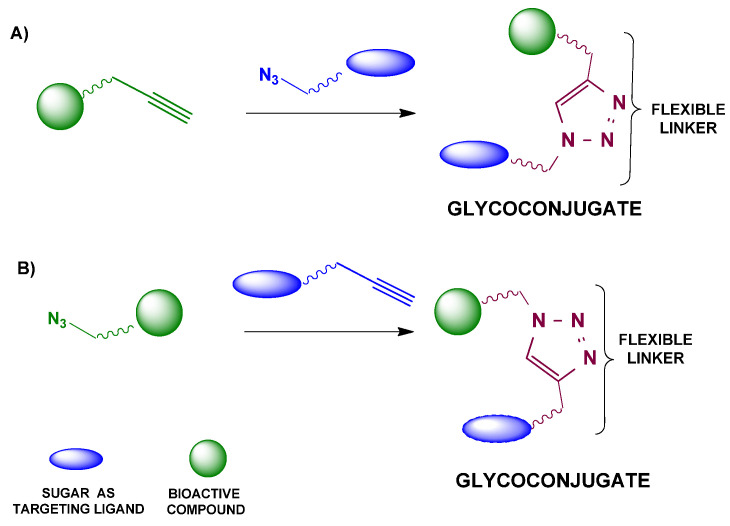
General scheme of two possible approaches to glycoconjugates synthesis with click chemistry concept. (**A**) Reaction of propargyl derivative of betulin with sugar azide, (**B**) Reaction of an azide derivative of betulin with a propargyl sugar derivative.

The concept of click chemistry involves using two building blocks: one containing a terminal alkyne group and the other featuring an azide functional group. Depending on the selection of these reagents results in obtaining conjugates with different spatial orientations of the 1,2,3-triazole ring in the linker structure (Scheme 1).

The results of our previous studies have indicated that carrying out the CuAAC reaction using a propargyl derivative of betulin and a sugar derivative containing an azide group is favorable, from the glycoconjugates cytotoxicity point of view [28]. In this work, the attention was focused on betulin glycoconjugates containing only one sugar residue attached at the C28 position of betulin. It was also decided to assess whether the presence of an acetyl group at the C3 position of the betulin skeleton affects the pharmacokinetic properties of the new glycoconjugates.

**Scheme 2:**
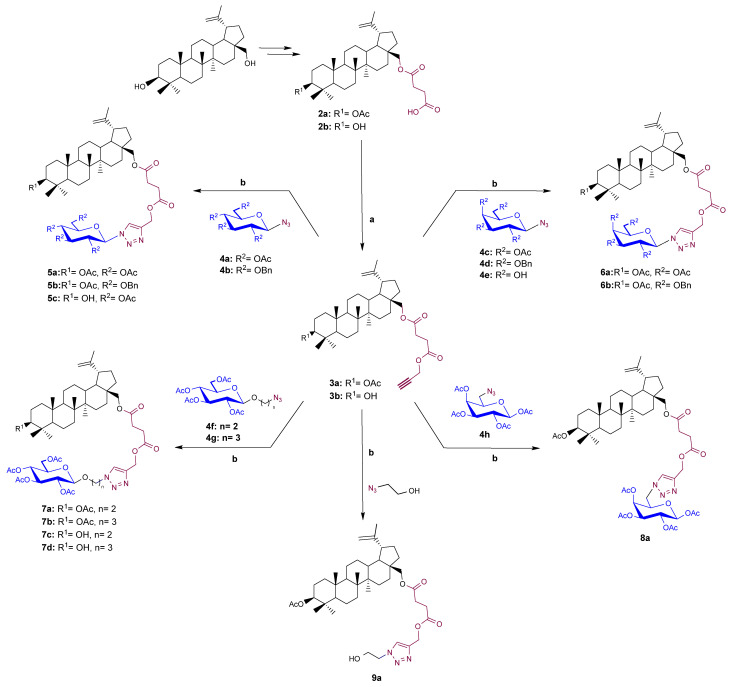
Synthesis of BN glycoconjugates (**5**–**8**) and potential metabolite (**9a**). Reagents and Conditions: (a) propargyl bromide, K_2_CO_3_, DMF, r.t., 5 days; (b) CuSO_4_·5H_2_O, NaAsc, i-PrOH/THF/H_2_O (1:1:1, *v*:*v*:*v*), r.t., 7 days.

For this reason, 3-*O*-acetyl-28-*O*’-(3-propynylcarbonyloxypropanoyl)betulin **3a** functionalized at the C28 position with succinic linker terminated by propargyl fragment, and its equivalent **3b** with a free hydroxyl group at the C3 position, were obtained (Scheme 2).

### 3.1. Betulin Analogues with Alkyne Moiety (***3***)

In the first step of the synthesis, according to the protocol described by Thibeault et al. [38] with some modifications, 3,28-*O*,*O*’-diacetylbetulin and 3-*O*-acetylbetulin were obtained. Then, following the published procedure [39], 3-*O*-acetylbetulin was esterified at 125 °C for 18 h, with an excess of succinic anhydride (SA) in dry pyridine (Py), and in the presence of the catalytic amount of 4-(dimethylamino)pyridine (DMAP). The crude product was purified with chloroform extraction, followed by washing with water and HCl solution, and further column chromatography. 3-*O*-Acetyl-28-*O*’-(3-carboxypropanoyl)betulin **2a** was obtained with a high yield (85%). The synthesis of 28-*O*-(3-carboxypropanoyl)betulin **2b** was performed similarly, but reagents were used in a molar ratio of BN:SA (1:1.5), and reaction time was reduced to 8 h. Because of the higher reactivity of the primary hydroxyl group at the C28 position of the parent BN backbone (C^28^-OH) compared to the reactivity of tertiary OH group at the C3 position (C^3^-OH), the reaction time needed to be carefully controlled. It was possible to obtain product **2b** with a high yield (67%).

The next step of our research involved the introduction of an alkyne moiety to the linker terminated by the carboxyl group ((O(CO)CH_2_CH_2_COOH). BN analogues (**2a**, **2b**), dissolved in dimethylformamide (DMF) were reacted with propargyl bromide in the presence of potassium carbonate, in a molar ratio (**2**/HC≡CCH_2_Br/K_2_CO_3_, 1:6.6:3.5). After 5 days of stirring at room temperature, the product was isolated by extraction with diethyl ether, with **3a**: 96%, **3b**: 75% yields, respectively.

### 3.2. Sugar Derivatives (***4***)

Synthesis of sugar units was performed according to the previously described procedures [40,41,42,43,44,45] (the details are provided in the Supplementary Materials). The ability of GLUT to bind and transport glycoconjugates may be influenced by several factors, such as the carbohydrate structure, the position of its substitution and the linker type [46,47,48]. Due to that, for betulin glycoconjugation, both D-glucose and D-galactose derivatives (**4a**–**4h**) were selected, in which the hydroxyl groups were protected by acetyl or benzyl groups. The choice of protecting groups was dictated primarily by their sensitivity to enzymatic hydrolysis. Acetyl groups are relatively susceptible to the action of hydrolytic enzymes in the body. In turn, the benzyl groups exhibit greater stability and resistance to enzymatic hydrolysis, compared to acetyl groups. This makes them appear to be particularly useful in the context of transport across cell membranes.

**Figure 5:**
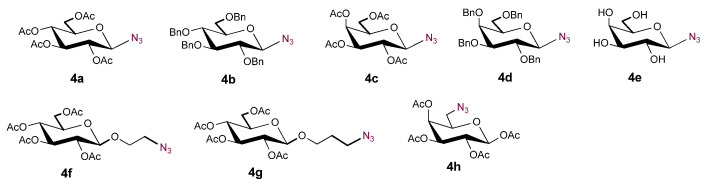
Structures of sugar derivatives used for glycoconjugation of betulin.

According to literature reports, highlighting the importance of the substituent orientation at the anomeric position of sugars for effective binding to GLUT1 transporters [49,50,51], we designed and synthesized sugar derivatives **4a**–**4g** with the β-configuration. The analysis of the crystal structure of human GLUT1 indicates that the hydroxyl group of D-glucose at the C6 position exhibits minimal involvement in the formation of hydrogen bonds with amino acid residues within this protein transporter [52]. Therefore, modification of the sugar unit at the 6-OH position should not affect receptor binding as strongly as modification at the anomeric position of the sugar. To maintain the affinity of the molecule to the GLUT1 transporter, it was decided to use a sugar **4h** functionalized in the 6-OH position for glycoconjugation and link it through this position to the betulin derivative, i.e., the fragment responsible for inducing the cytotoxic effect. The structures of all sugar derivatives used for glycoconjugation of betulin derivatives are presented in Figure 5.

### 3.3. Synthesis of Betulin Glycoconjugates via Click Chemistry (***5***–***8***)

In the course of our research, we designed a library of new BN glycoconjugates, not described in the scientific literature so far, and then developed a method for their synthesis, using the concept of click chemistry as shown in Scheme 2. The copper-catalyzed 1,3-dipolar azide-alkyne cycloaddition reaction (CuAAC) provides a simple and efficient method for the synthesis of molecular hybrids based on natural products, and its main advantage is the mild reaction conditions.

We have used building blocks, enabling the preparation of a whole range of glycoconjugates, containing in the structure a linker built from a succinyl fragment and a heteroaromatic 1,2,3-triazole ring, and in the case of compounds (**7a**–**7d**), an additional chain O(CH_2_)_n_. Such an elaborate linker was used to eliminate stiffening of the entire hybrid, which should ensure a better alignment of the molecule to sugar transporters.

The general procedure for glycoconjugation involves contacting of 3-*O*-acetyl-28-*O***’**-(propynylcarbonyloxypropanoyl)betulin **3a** or 28-*O*’-(propynylcarbonyloxypropanoyl) betulin 3b with the selected sugar unit (**4a**–**4h**) in the presence of copper(II) sulfate (CuSO_4_-5H_2_O) as a catalyst and sodium ascorbate (NaAsc) as a Cu(II) to Cu(I) reducing agent. The reactions were carried out in the solvent system water-isopropyl alcohol (*i*-PrOH)- tetrahydrofuran (THF) under an argon atmosphere at room temperature for 7 days. The reaction progress was monitored with TLC method. As a result, nine new glycoconjugates of BN (**5**–**7**) were isolated by column chromatography in very high yields, within the range of 53–73% (Table 1). Such a good yield was not achieved only in the case of glycoconjugate **8a** (12% yield), which was attributed to the lower purity of the sugar derivative **4h** used in the reaction. Unfortunately, when the unprotected galactose derivative **4e** was used for the reaction, it was not possible to obtain the desired glycoconjugate. This was probably due to too low concentration of the sugar substrate in the reaction mixture, which is related to its poor solubility in the solvent system applied.

### 3.4. Synthesis of Metabolite (***9a***)

The molecules of betulin glycoconjugates, synthesized by our team, are built from several basic building blocks: (i) the betulin backbone, having a succinic linker at the C28 position, (ii) the sugar unit, and (iii) the linker, containing an aromatic 1,2,3-triazole ring. Inside the cell, the glycoconjugates can be subjected to the action of hydrolytic enzymes, resulting in the release of a potential metabolite. Given the stability of the triazole system under typical physiological conditions, we set out to synthesize and then assay the cytotoxicity of a potential metabolite **9a** that could be formed under the action of hydrolytic enzymes.

In this case, we also carried out the CuAAC reaction, in which we used 3-*O*-acetyl-28-*O*’-(propynylcarbonyloxypropanoyl)betulin **3a** and 2-azidoethanol in the presence of copper(II) sulfate (CuSO_4_-5H_2_O) and sodium ascorbate (NaAsc), in a molar ratio (3a/HC≡CCH_2_N_3_/ CuSO_4_-5H_2_O/ NaAsc, 1:1:0.2:0.4), in THF/iPrOH/water solvent system. We isolated metabolite **9a** by column chromatography with 32% yield.

The structures of all synthesized compounds were confirmed by nuclear magnetic resonance (^1^H-, ^13^C-NMR) and high-resolution mass spectrometry (HRMS). NMR spectra of all newly synthesized products are presented in the Supplementary Materials.

### 3.5. Evaluation of Cytotoxicity

The series of structurally diverse betulin glycoconjugates underwent preliminary cytotoxicity screening using the MTT assay (3-(4,5-dimethylthiazol-2-yl)-2,5-diphenyltetrazolium) bromide) [53]. Two cancer cell lines were chosen for the study: HCT 116 (*colorectal carcinoma cell line*) and MCF-7 (*human breast adenocarcinoma cell line*). This selection was not random, as in both types of cancer cell lines overexpression of glucose and galactose transporters is observed [54,55,56]. To determine if glycoconjugation enhanced the selectivity of the betulin derivatives, cytotoxicity tests on *Normal Human Dermal Fibroblast-Neonatal cells* (NHDF-Neo) were also conducted. The selection of this healthy cell line, so atypical in relation to the cancer cells tested, was based on the desire to compare the selectivity index determined for the currently obtained glycoconjugates with the results obtained for the betulin derivatives we described earlier [28] and draw preliminary conclusions on structure-selectivity relationships. All studies were carried out in the concentration range from 25 to 200 µM. The incubation time for individual cell lines varied from 24 to 72 h, due to differences in the doubling time of these cell lines. In the case of HCT 116 and NHDF-Neo cell lines, a shorter incubation time (24 or 48 h) was used, since, according to various literature sources, the doubling time for these cells ranges from 18 to 24 h [57]. In contrast, a longer incubation time (72 h) was chosen for MCF-7 cells, as literature reports on this cell line indicate a much longer doubling time of 35 to 72 h, depending on the source [58,59].

In the first step, the cytotoxicity of the building blocks, i.e., sugar units and betulin was assessed against the tested cell lines. While the sugar derivatives used for glycoconjugation proved to be inactive, betulin inhibited the proliferation of both cancerous and healthy cells. In this case, the difference in the determined IC_50_ values was negligible (34.88 ± 4.26 µM for HCT-116, 17.89 ± 1.06 µM for MCF-7, and 34.90 ± 8.36 µM for NHDF-Neo), indicating the need for modifications aimed to enhance the selectivity of betulin towards cancer cells.

In designing the molecular hybridmade of betulin and sugar unit, we focused on the differences in properties between cancerous and normal cells. The incorporation of a sugar moiety in the structure of the new hybrid was aimed at targeting the cells that overexpress sugar transporters, which is associated with the previously mentioned Warburg effect. Additionally, we considered factors that could enhance the affinity of betulin glycoconjugates for the interphase space. The presence of the basic 1,2,3-triazole ring should increase the concentration of glycoconjugates in the interphase space of cancer cells, which is known to have a mildly acidic environment, resulting from the cells’ excretion of lactate produced by excessive glycolysis in tumors [60,61]. The insertion of a 1,2,3-triazole ring together with a succinic linker was also intended to enable the formation of complexes with copper ions, which should further inhibit the proliferation of cancer cells by targeting a critical factor in their growth. Although patients with various malignancies, including colorectal and breast cancer, typically exhibit elevated levels of copper in their serum or tissues, cancer cells cultured in vitro are characterized by low copper levels [62,63]. Consequently, it seemed justified to conduct additional experiments with the addition of Cu^2+^ ions. First, the effect of a 20 µM copper chloride solution on the proliferation of cancer and healthy cells was assessed. Once confirmed that such a concentration of Cu^2+^ ions did not adversely affect the viability of the cell lines examined, we proceeded with further experiments aimed at investigating the influence of metal ion complexation by glycoconjugates on cell proliferation.

**Figure 6:**
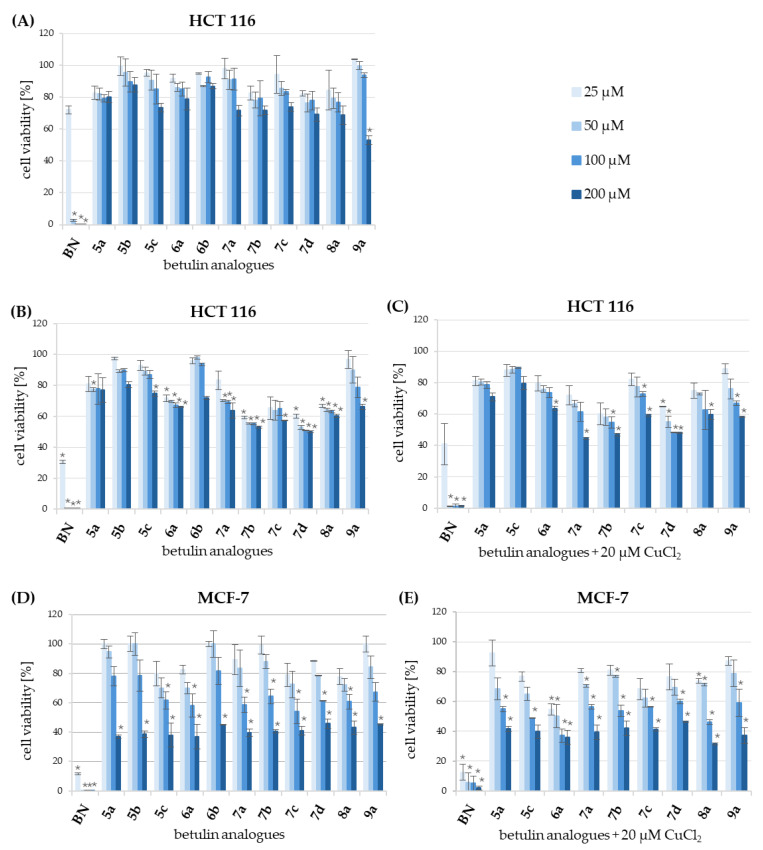
Dependence of HCT 116 and MCF-7 cells’ proliferation on concentration of BN glycoconjugates as estimated using MTT assay compared to BN alone: (**A**) proliferation of HCT 116 cells after 24 h incubation, (**B**) proliferation of HCT 116 cells after 48 h incubation, (**C**) proliferation of HCT 116 cells after 48 h incubation in presence of 20 µM copper ions (Cu^2+^), (**D**) proliferation of MCF-7 cells after 72 h incubation, (**E**) proliferation of MCF-7 cells after 72 h incubation in presence of 20 µM copper ions (Cu^2+^). Data are presented as mean ± standard deviation (*n* = 3). Statistical analysis was performed two-way ANOVA with replication followed by post hoc *t*-tests with Bonferroni correction. (*) *p*-value < 0.05, in comparison to untreated control.

**Figure 7:**
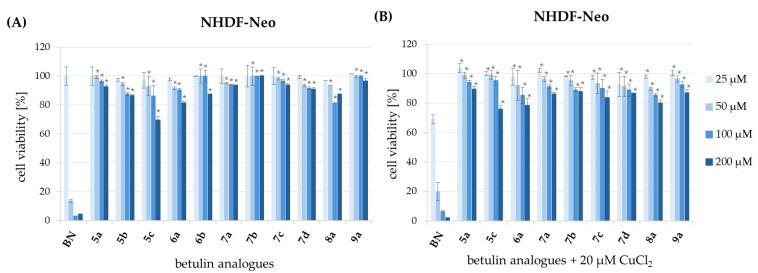
Dependence of NHDF-Neo cells’ proliferation on concentration of BN glycoconjugates, as estimated using MTT assay compared to BN alone, in absence (**A**) and presence (**B**) of 20 µM of Cu^2+^ after 24 h. Data are presented as mean ± standard deviation (*n* = 3). Statistical significance was calculated from two-way ANOVA with replication, followed by post hoc *t*-tests with Bonferroni correction, where *p* < 0.05 and is indicated by an asterisk (*).

Figure 6 and Figure 7 illustrate the relationship between cell proliferation and concentrations of BN, glycoconjugates (**5**–**8**) or metabolite **9a**. The IC_50_ values, defined as 50% cell growth inhibition compared to the untreated control, have been collected in Table 2.

The cytotoxicity of designed BN glycoconjugates against HCT 116 and MCF-7 is generally lower than that of native betulin. The tested glycoconjugates inhibited cell growth in a dose-dependent manner. After 24 h of treatment with the tested compounds, the HCT 116 cell line exhibited high survivability. After increasing the incubation time to 48 h, the HCT 116 cell line became more sensitive to the presence of the tested derivatives, particularly for glycoconjugates (**7a**–7**d**, Figure 6). For compound **7d**, the IC_50_ value was determined to be 131.16 ± 8.76 µM. The results obtained indicate that for the HCT 116 cell line, the strategy of elongating the linker between the sugar moiety and the BN backbone was effective in reducing the stiffness of such a large molecule. Additionally, the presence of an unprotected hydroxyl group at the C3 position of BN proved to be significant for the biological activity of the glycoconjugates. Notably, the compounds tested displayed minimal cytotoxicity towards healthy NHDF-Neo cells (Figure 7), with the IC_50_ values remaining indeterminable for all conjugates within the tested concentration range.

Conversely, the MCF-7 cell line demonstrated the highest sensitivity to both BN and its glycoconjugates. Although for glycoconjugates the determined IC_50_ values were considerably higher than those for BN alone, it was possible to define the IC_50_ values for all tested conjugates (Table 2). One of the possible explanations for this state of affairs may be the proven and described in the literature overexpression of GLUT transporters (especially GLUT1) in this type of cancer [64]. However, confirmation of this thesis requires further studies using equivalents of the described glycoconjugates with a deprotected sugar moiety.

The most active glycoconjugates were (**5c**, **6a**, and **7c**), with determined IC_50_ values of 128.07 ± 1.35 µM, 122.61 ± 6.84 µM, and 134.92 ± 7.92 µM, respectively. Analyzing the relationship between the structure and cytotoxicity of the tested compounds, it is evident that the presence of an unprotected hydroxyl group at the C3 position of BN (as in the case of compounds **5c** and **7c**) plays a significant role. Notably, substituting the D-glucose residue with D-galactose significantly increased the cytotoxicity of compound **6a** compared to the analogous derivative **5a** that contains a D-glucose residue (IC_50_ values of 122.61 ± 6.84 µM and 163.12 ± 7.65 µM, respectively). Conversely, in this context, the rigidity of the linker between the sugar moiety and the BN derivative appeared to be less significant. The most active compounds did not include the two- or three-carbon alkyl chain typical of structures (**7a**–**7d**).

Interesting results emerged from an additional experiment designed to demonstrate the potential mechanism of action of the synthesized glycoconjugates, which involves the chelation of copper ions, essential for cancer cells growth. The antiproliferative activity of BN derivatives were evaluated in the presence of Cu^2+^ ions. To assess the impact of these complexes with metal ions on cell proliferation, a copper chloride solution was added to the growth medium, and the rate of cell growth in the presence of BN conjugates was compared to that of untreated cells. In the case of the HCT 116 cell line, a notable effect was observed for compounds (**7a**–**7d**), which had previously been identified as the most effective against these cells. After introducing copper ions, the IC_50_ values for compounds **7a** and **7b** were determined to be 150.08 ± 8.01 µM and 112.48 ± 0.57 µM, respectively. Remarkably, compound **7d** exhibited the highest cytotoxic activity, with its IC_50_ value decreasing more than twofold under these conditions, compared to the value obtained in the absence of copper ions (60.40 ± 6.95 µM versus 131.16 ± 8.76 µM). For the NHDF-Neo cell line, only minor variations in cell viability were observed after treatment with the tested compounds alongside copper ions (Figure 7). This indicates that the viability of healthy cells was not as significantly affected by copper ion concentration as in the cancer cell line.

This observation highlights an increased selectivity of glycoconjugate **7d**, compared to BN, as evidenced by the selectivity index (SI), calculated as the ratio of the IC_50_ value for healthy cells (NHDF-Neo) to the IC_50_ value for cancer cells. For BN, in the presence of copper ions, the SI value against HCT 116 cells was 1.6, whereas for glycoconjugate **7d**, the SI value exceeded 3.3. In the case of the MCF-7 cell line, the introduction of copper ions also increased the cytotoxicity of the tested compounds, as indicated by the IC_50_ values. Notably, the most significant differences were observed for glycoconjugates **5c**, **6a**, and **8a**, which exhibited IC_50_ values of 70.71 ± 5.44 µM, 59.81 ± 1.85 µM, and 88.33 ± 3.31 µM, respectively. For the most active glycoconjugate, **6a**, the selectivity index (SI) improved to a value greater than 3.3, compared to a SI of 2.4 for BN. Interestingly, glycoconjugate **8a**, which features the D-galactose derivative linked to BN via the C6 position, was not more effective than analogous glycoconjugate **6a**, which is linked to BN through the C1 position (with IC_50_ values of 88.33 ± 3.31 µM and 59.81 ± 1.85 µM, respectively). This suggests that glycoconjugation through the C6 position in the sugar does not enhance the affinity of the final glycoconjugate for sugar transporters.

In the course of the studies, the cytotoxicity of potential metabolite **9a**, which may be generated through the hydrolytic action of glycosidases on glycoconjugate **7a**, was assessed. The relatively low cytotoxicity of this compound suggests that premature hydrolysis of glycoconjugates before reaching the target cancer cells is unlikely to pose a threat to healthy cells.

### 3.6. Molecular Descriptors

Determining the physicochemical properties of compounds is crucial in drug design, because these properties directly affect pharmacokinetics. One of the most widely known and used guidelines for assessing potential bioavailability is Lipinski’s Rule of Five (Ro5), proposed by Christopher Lipinski in 1997. This rule helps predict whether a compound can be effectively absorbed into the human body. According to Ro5, regarding orally administered drugs, a drug should meet the following criteria: molecular weight (MW) ≤ 500 Da, as larger molecules may have difficulty crossing cell membranes, thus affecting bioavailability. Additionally, the octanol–water partition coefficient (logP) should be ≤ 5; higher values indicate poor water solubility, which leads to lower bioavailability. The number of hydrogen bond donors should be ≤ 5, as groups like OH and NH can impact solubility, and an excessive number may hinder membrane permeability. The number of hydrogen bond acceptors should be ≤ 10, because its higher value can negatively affect drug absorption. Although not an absolute requirement, compounds that violate more than one of these criteria are less likely to be successful as oral drugs [65].

Another important descriptor to consider is the topological polar surface area (TPSA), which defines the molecule’s ability to penetrate biological membranes. Good oral absorption is typically associated with TPSA ≤ 140 Å^2^. However, certain compound classes do not fully follow these rules, but still demonstrate good pharmacokinetics. This is particularly true for antibiotics, biological drugs, and natural medical products [66]. It should be taken into account that the increasing diversity of drug compounds and therapeutic applications may require a more comprehensive strategy. Depending on the pharmacological class of the compound and its target profile, its bioavailability, solubility, and permeability may be affected [67].

Analyzing molecular descriptors plays a key role in the early stages of drug design. Using computational tools such as *Molinspiration* online software (http://www.molinspiration.com), scientists can predict the properties of a molecule and make structural modifications to improve bioavailability and efficacy, and also minimize potential problems related to toxicity or poor solubility. It is important to note, however, that there are exceptions to these rules.

Glycoconjugates (**5a**–**c**, **6a**–**b**, **7a**–**d**, and **8a**, Table 3) have relatively high molecular weights exceeding 500 g/mol, and their TPSA also exceeds the 140 Å^2^ threshold. Additionally, the high number of hydrogen bond acceptors suggests that these compounds may face difficulties in membrane transport. According to Ro5, the compounds we designed do not meet the criteria for oral drugs. However, they may be suitable for topical use or intravenous injection, especially when it turns out that they show the expected selectivity of action.

The only compound that partially meets the Ro5 criteria is the potential metabolite **9a**. However, our goal was to design selective betulin glycoconjugates capable of releasing biologically active metabolites specifically at the target site, near cancer cells. Therefore, oral administration of the metabolite itself is not a priority in our therapeutic approach.

## 4. Conclusions

Focusing on a common characteristic of cancer—namely, the increased demand for glucose and the corresponding overexpression of its transporters, as well as the fact that cancer cell growth and their metastasis are strongly related to copper ions uptake—a strategy has been proposed to enhance the efficacy and selectivity of compounds with anticancer activity.

Based on these assumptions, several betulin glycoconjugates were designed and synthesized, which structures and purity were confirmed with spectroscopic analysis. These compounds were subsequently evaluated for cytotoxicity. Even though the determined IC_50_ values suggest that the described betulin glycoconjugates may not be suitable for use as potential drugs, certain patterns in their structure, influencing the activity and selectivity, have been identified.

The obtained results revealed that the activity of the glycoconjugates is influenced by several factors. These include the type of sugar fragment added, the type of protective groups present in the sugar unit, the absence of protection on the hydroxyl group in the C3 position of betulin, and the length of the linker connecting the sugar components to the betulin derivative. The incorporation of a heteroaromatic 1,2,3-triazole ring, along with the succinyl linker, affects the capacity to chelate metal ions present in many types of cancer cells. The observed improvement in the selectivity of tested glycoconjugates may be because healthy cells are less sensitive to changes in copper ion concentration.

The results presented in this work encourage further optimization of the structure of betulin derivatives. The next logical step appears to be the creation of glycoconjugates that feature an unprotected sugar unit, and checking whether the presence of such a ligand allows for the utilization of their potential against cells that overexpress sugar transporters. Expanding the library of betulin glycoconjugates will facilitate the identification of specific structural elements responsible for eliciting biological activity.

## Figures and Tables

**Table 1:** Yields and HRMS date of obtained betulin glycoconjugates (**5a**–**5c**, **6a**, **6b**, **7a**–**7d**, **8a**) and potential metabolite **9a**.

BN Analogue	Sugar	Product				HRMS ([M+Na]^+^)		
		No.	R^1^	R^2^	Yield (%)	Summary Formula	Calc.	Found.
**3a**	**4a**	**5a**	OAc	OAc	72	C_53_H_75_N_3_O_15_	1018.5252	1018.5353
**3a**	**4b**	**5b**	OAc	OBn	60	C_73_H_93_N_3_O_11_	1210.6708	1210.7081
**3b**	**4a**	**5c**	OH	OAc	64	C_51_H_75_N_3_O_14_	976.5147	976.5180
**3a**	**4c**	**6a**	OAc	OAc	71	C_53_H_75_N_3_O_15_	1018.5252	1018.5360
**3a**	**4d**	**6b**	OAc	OBn	53	C_73_H_93_N_3_O_11_	1210.6708	1210.6998
**3a**	**4f**	**7a**	OAc	OAc	73	C_55_H_81_N_3_O_16_	1062.5515	1062.5526
**3a**	**4g**	**7b**	OAc	OAc	69	C_56_H_83_N_3_O_16_	1076.5671	1076.5715
**3b**	**4f**	**7c**	OH	OAc	59	C_53_H_79_N_3_O_15_	1020.5409	1020.5486
**3b**	**4g**	**7d**	OH	OAc	70	C_54_H_81_N_3_O_15_	1034.5565	1034.5626
**3a**	**4h**	**8a**	OAc	OAc	12	C_53_H_75_N_3_O_15_	1018.5252	1018.5356
**3a**	2-azidoethanol	**9a**	OAc	-	32	C_41_H_63_N_3_O_7_	732.4564	732.4573

**Table 2:** Cytotoxic activity of betulin (BN), betulin glycoconjugates (**5a**–**5c**, **6a**, **6b**, **7a**–**7d**, **8a**), and metabolite **9a** ^a^.

Compound	HCT 116			MCF-7		NHDF-Neo
	IC_50_ [µM] ^b^	IC_50_ [µM] ^c^	SI	IC_50_ [µM] ^d^	SI	IC_50_ [µM] ^b^
**BN**	34.88 ± 4.26	25.96 ± 5.40	1.3	17.89 ± 1.06	1.9	34.90 ± 8.36
**BN + Cu^2+ e^**	-	20.76 ± 2.71	1.6	13.91 ± 2.35	2.4	33.34 ± 5.97
**5a**	>200	>200	-	163.12 ± 7.65	>1.2	>200
**5a + Cu^2+ e^**	-	>200	-	108.51 ± 5.85	>1.8	>200
**5b**	>200	>200	-	167.61 ± 11.66	>1.2	>200
**5c**	>200	>200	-	128.07 ± 1.35	>1.6	>200
**5c + Cu^2+ e^**	-	>200	-	70.71 ± 5.44	>2.8	>200
**6a**	>200	>200	-	122.61 ± 6.84	>1.6	>200
**6a + Cu^2+ e^**	-	>200	-	59.81 ± 1.85	>3.3	>200
**6b**	>200	>200	-	184.13 ± 7.59	>1.1	>200
**7a**	>200	>200	--	144.12 ± 4.70	>1.4	>200
**7a + Cu^2+ e^**	-	150.08 ± 8.01	>1.3	135.01 ± 10.23	>1.5	>200
**7b**	>200	>200		152.23 ± 2.91	>1.3	>200
**7b + Cu^2+ e^**	-	112.48 ± 0.57	>1.8	124.29 ± 1.64	>1.6	>200
**7c**	>200	>200	-	134.92 ± 7.92	>1.5	>200
**7c + Cu^2+ e^**	-	>200	-	117.94 ± 1.42	>1.7	>200
**7d**	>200	131.16 ± 8.76	>1.5	165.02 ± 8.32	>1.2	>200
**7d + Cu^2+ e^**	-	60.40 ± 6.95	>3.3	147.02 ± 2.32	>1.4	>200
**8a**	>200	>200	-	152.63 ± 1.98	>1.3	>200
**8a + Cu^2+ e^**	-	>200	-	88.33 ± 3.31	>2.3	>200
**9a**	>200	>200		147.56 ± 8.71	>1.4	>200
**9a + Cu^2+ e^**	>200	>200		138.8 ± 6.21	>1,4	>200
**Doxorubicin**	13.17 ± 0.58	1.89 ± 0.19	>10.6	0.29 ± 0.04	>70	>20

^a^ Cytotoxicity was assessed by MTT assay; incubation time: ^b^ 24 h; ^c^ 48 h; ^d^ 72 h, ^e^ Addition of CuCl_2_ at 20 µM. SI: selectivity index; ratio of IC_50_ of healthy cells (NHDF-Neo) to IC_50_ of cancer cells.

**Table 3:** Molecular descriptors analysis of BN glycoconjugates using *Molinspiration 2025* online software tools (http://www.molinspiration.com; accessed on 8 February 2025).

Structure	pLogP	TPSA (Å^2^)	MW (g/mol)	Vol (Å^3^)	nON	nOHNH	nrotb
**5a**	7.96	224.09	996.21	930.36	18	0	22
**5b**	9.94	155.80	1188.56	1141.03	14	0	26
**5c**	7.25	218.01	954.17	893.85	17	1	20
**6a**	7.96	224.09	996.21	930.36	18	0	22
**6b**	9.94	155.80	1188.56	1141.03	14	0	26
**7a**	7.94	233.32	1040.26	972.95	19	0	25
**7b**	8.19	233.32	1054.29	989.75	19	0	26
**7c**	7.24	227.24	998.22	936.44	18	1	23
**7d**	7.51	227.24	1012.25	953.24	18	1	24
**8a**	7.96	224.09	996.21	930.36	18	0	22
**9a**	7.27	129.86	709.97	694.78	10	1	14

pLogP: predicted LogP; TPSA: topological polar surface area; MW: molecular weight; Vol: molecular volume; nON: number of hydrogen bond acceptors; nOHNH: number of hydrogen bond donors; nrotb: number of rotatable bonds.

## Data Availability

All compounds described in this article are derived from research for a doctoral thesis carried out at the Faculty of Chemistry, Silesian University of Technology. Spectroscopic data of all compounds are available from the authors and will be made available upon sending such a request to the e-mail address: miroslawa.grymel@polsl.pl.

## Supplementary Materials

The following supporting information can be downloaded at: https://www.mdpi.com/article/10.3390/cancers17111737/s1, Details of chemical synthesis and characterization data of compounds, Scheme S1: Synthesis of sugar derivatives **4a**–**4e**, Scheme S2: Synthesis of sugar derivatives **4f**–**4g**, Scheme S3: Synthesis of sugar derivative **4h**, NMR spectra of betulin glycoconjugates.
