# Supplementary material for: Synthesis and Preliminary Cytotoxicity Evaluation of 3-Lup-20(29)-Ene-3β,28-Diol Glycoconjugates Containing a Succinic Linker and a 1,2,3-Triazole Ring
Source: Cancers (Basel). 2025 May 22;17(11):1737. doi: 10.3390/cancers17111737 (PMC12153776; doi:10.3390/cancers17111737)
Supplement: Supplementary file 1 [file cancers-17-01737-s001.zip › cancers-3593928-supplementary.pdf]

Supplementary Materials

Synthesis and Preliminary Cytotoxicity Evaluation of 3-Lup-20(29)-ene-3β,28-diol Glycoconjugates Containing a Succinic Linker and a 1,2,3-Triazole Ring

Julia Szreder, Klaudia Woźniak, Karol Erfurt, Mirosława Grymel and Gabriela Pastuch-Gawołek

TABLE OF CONTENTS

1. General Experimental Information ……………………………………………………………………………S2
2. Details of chemical synthesis and characterization data of compounds ……………………………………S2
2.1. Procedure for the Synthesis of Betulin Analogs (**2** and **3**) ……………………………………………S2
2.1.1. Experimental Procedure for the Synthesis of 3-*O*-Acetyl-28-*O'*-(3-carboxypropanoyl)betulin (**2a**)..S2
2.1.2. Experimental Procedure for the Synthesis of 28-*O'*-(3-Carboxypropanoyl)betulin (**2b**) …………S2
2.1.3. Experimental Procedure for the Synthesis of Betulin Analogs (**3**) ……………………………………S3
2.2. Procedure for the Synthesis of Sugar Derivatives ………………………………………………………S4
2.2.1. Experimental Procedures for the Synthesis of Sugar **4a** and **4c** ………………………………………S4
2.2.2. Experimental Procedures for the Synthesis of Sugar 4b and **4d** ……………………………………… S5
2.2.3. Experimental Procedures for the Synthesis of Sugar **4f** and **4g** ……………………………………… S6
2.2.4. Experimental Procedures for the Synthesis of Sugar **4h** ……………………………………………… S7
2.3 General Procedure for the Synthesis of Betulin Glycoconjugates (**5** – **8**) …………………………S8
2.4. Procedure for the Synthesis of Betulin Metabolite (**9a**) ………………………………………………S12
3. NMR Spectra of Betulin Glycoconjugates (**5** – **8**) ………………………………………………………S13
4. References ……………………………………………………………………………………………………S24

1. General Experimental Informations

The ^{1}H and ^{13}C NMR spectra were recorded in $CDCl_3$ (ACROS Organics, Geel, Belgium), using tetramethylsilane (TMS) as internal shift standard (^{1}H NMR, zero ppm) or residual chloroform (^{13}C NMR, 77.16 ppm). Measurements were performed on an Varian spectrometer at operating frequencies of 400 MHz or 600 MHz. The NMR spectra were analyzed based on chemical shift values (δ) in parts per million (ppm), and coupling constants (*J*) in hertz (Hz). The observed multiplicity of signals are designated: singlet (s), doublet (d), double doublet (dd), triplet (t), overlapping double doublet that resembles a triplet (dd~t), multiplet (m), broad (br). High resolution mass spectrometry (HRMS) analyses were performed on a Waters Xevo G2 Q-TOF mass spectrometer (Waters Corporation) equipped with an electrospray ionization source (ESI) operating in positive-ion modes. The accurate mass and composition for the molecular ion adducts were calculated using the *MassLynx software (Waters)* incorporated with the instrument. Optical rotations were measured at room temperature using a JASCO P-2000 polarimeter equipped with a sodium lamp (589.3 nm). Melting points were determined with an OptiMelt (MPA 100) from Stanford Research Systems (Sunnyvale, CA, USA). Reactions were monitored by thin-layer chromatography using TLC Silica Gel 60 F254 (Merck Millipore, Burlington, MA, USA). The TLC plates were examined by charring after spraying with a 10% sulfuric acid solution in ethanol or under UV light (λ = 254 nm). Purification of crude compounds was carried out by column chromatography using Silica Gel (Merck, 0.040–0.063 mm). All chemical reagents used in the synthesis were purchased from ACROS Organics (Geel, Belgium) or Avantor (Gliwice, Poland) and used without further purification.

2. Details of chemical synthesis and characterization data of compounds

3-*O*-Acetylbetulin **1a** [1], 3-*O*-acetyl-28-*O*′-(3-carboxypropanoyl)betulin **2a** [2], 28-*O*′-(3-carboxypropanoyl)betulin **2b** [2], 1-azido-2,3,4,6-tetra-*O*-acetyl-β-D-glucopyranose **4a** [3], 1-azido-2,3,4,6-tetra-*O*-acetyl-β-D-galactopyranose **4c** [4], 1-azido-2,3,4,6-tetra-*O*-benzyl-β-D-glucopyranose **4b**, 1-azido-2,3,4,6-tetra-*O*-benzyl-β-D-galactopyranose **4d** [5], 1-azido-β-D-galactopyranose **4e** [6], 1-*O*-(2-azidoethyl)-2,3,4,6-tetra-O-acetyl-β-D-glucopyranose **4f** and 1-*O*-(3-azidopropyl)-2,3,4,6-tetra-*O*-acetyl-β-D-glucopyranose **4g** [7], 6-azido-1,2,3,4-tetra-*O*-acetyl-β-D-galactopyranose **4h** [8] were prepared according to the respective published procedures.

2.1. Procedure for the Synthesis of Betulin Analogs (2 and 3)

2.1.1. Experimental Procedure for the Synthesis of 3-*O*-Acetyl-28-*O*′-(3-carboxypropanoyl)betulin (2a)

3-*O*-Acetylbetulin (**1a**, 2.21 g, 4.6 mmol), succinic anhydride (1.38 g, 13.8 mmol), DMAP (1.53 g, 12.5 mmol) and pyridine (36 mL) were mixed in a 100 mL round-bottom flask and heated at 125°C for 18 hours with reflux. After cooling, 10% HCl (34 mL) and distilled water (60 mL) were added. The solution was extracted with chloroform (5 × 100 mL), monitored by thin-layer chromatography (TLC, DCM/MeOH, 10:1, v/v). The organic layer was washed with water (100 mL), 5% hydrochloric acid solution (160 mL), brine (10 mL), and water again. The organic layer was dried over anhydrous $MgSO_4$, the solvent evaporated under vacuum. Crude product **2a** was was purified by column chromatography (DCM/MeOH, gradient 100:1 to 50:1, v/v).

3-O-Acetyl-28-O′-(3-carboxypropanoyl)betulin **2a** was obtained as a resin, 85% yield; ^{1}H NMR (600 MHz, $CDCl_3$) δ_H: 4.68 (s, br, 1H, H-29a), 4.59 (s, br, 1H, H-29b), 4.47 (dd, 1H, J_1 = 4.8 Hz, J_2 = 9.6 Hz, H-3), 4.26 (d, 1H, *J* = 10.8 Hz, H-28a), 3.88 (d, 1H, *J* = 10.8 Hz, H-28b), 2.67-2.57 (m, 4H, $O(CO)CH_2CH_2$), 2,43 (m, 1H, H-19), 2.04 (s, 3H, CH_3CO), 1.68 (s, 3H, CH_3-30), 1.98 - 0.78 (m, 24H, CH, CH_2, BN scaffold), 1.04, 0.96, 0.85, 0.84 (all s, 3H each, CH_3-23 – CH_3-27) ppm. ^{13}C NMR (150 MHz, $CDCl_3$) δ_C: 176.9 (CO), 172.4 (CO), 171.1 (CO), 150.1 (C-20), 109.9 (C-29), 80.9 (C-3), 63.2 (C-28), 55.4 (C-5), 50.3 (C-9), 48.8 (C-18), 47.7 (C-19), 46.4 (C-17), 42.7 (C-14), 40.9 (C-8), 38.4 (C-1), 37.8 (C-4), 37.6 (C-13), 37.1 (C-10), 34.5 (C-22), 34.1 (C-7), 29.7 (C-21), 29.5 (C-16), 29.1, 28.8 ($O(CO)\underline{C}H_2\underline{C}H_2$), 27.9 (C-23), 27.0 (C-15), 25.1 (C-12), 23.7 (C-2), 21.3 (C-11), 20.8 ($\underline{C}H_3CO$), 19.1 (C-30), 18.2 (C-6), 16.5 (C-25), 16.2 (C-26), 16.0 (C-24), 14.7 (C-27) ppm.

2.1.2. Experimental Procedure for the Synthesis of 28-O′-(3-Carboxypropanoyl)betulin (2b)

Betulin (**1b**, 1.11 g, 2.5 mmol), succinic anhydride (0.38 g, 3.75 mmol), DMAP (0.46 g, 3.75 mmol), and pyridine (20 mL) were mixed in a 100 mL round-bottom flask and heated at 125°C for 8 hours. After cooling, 10% HCl (40 mL) and $CHCl_3$ (50 mL) were added, then the mixture was extracted with $CHCl_3$ (4 × 70 mL), monitored by TLC (DCM/MeOH, 20:1, v/v). The organic layer was treated with water, 5% HCl, NaCl solution,

and water, then dried over anhydrous $MgSO_4$. Concentrated under reduced pressure. The crude product **2b** was purified by column chromatography (DCM/MeOH, gradient 100:1 to 20:1, v/v).

28-O′-(3-Carboxypropanoyl)betulin ***2b*** was obtained was obtained as a resin, 67% yield; ^{1}H NMR (600 MHz, $CDCl_3$) δ_H: 4.69 (s, br, 1H, H-29a), 4.59 (s, br, 1H, H-29b), 4.29 (d, 1H, *J* = 12.6 Hz, H-28a), 3.89 (d, 1H, *J* = 10.8 Hz, H-28b), 3.23 - 3.58 (s, br, 1H, OH), 3.17 (dd, 1H, J_1 = 5.4 Hz, J_2 = 11.4 Hz, H-3), 2.56-2.66 (m, 4H, $O(CO)CH_2CH_2$), 2.43 (td, 1H, J_1 = 5.8 Hz, J_2 = 11.1 Hz, H-19), 1.68 (s, 3H, CH_3-30), 0.67 - 2.08 (m, 24H, CH, CH_2, BN scaffold), 1.03, 0.97, 0.96, 0.82, 0.75 (all s, 15H, CH_3-23 –CH_3-27) ppm. ^{13}C NMR (150 MHz, $CDCl_3$) δ_c: 173.08 (CO), 172.32 (CO), 150.17 (C-20), 109.84 (C-29), 78.90 (C-3), 63.14 (C-28), 55.35 (C-5), 50.40 (C-9), 48.85 (C-18), 47.74 (C-19), 46.46 (C-17), 42.72 (C-14), 40.90 (C-8), 38.84 (C-1), 38.76 (C-4), 37.64 (C-13), 37.16 (C-10), 34.51 (C-22), 34.21 (C-7), 30.94 (C-21), 29.74 (C-16), 29.59, 29.38 ($O(CO)\underline{C}H_2\underline{C}H_2$), 27.93 (C-23), 27.09 (C-15), 27.06 (C-12), 25.24 (C-2), 20.80 (C-30, C-11), 19.13 (C-6), 18.31(C-26), 15.99 (C-25), 15.37 (C-24), 14.77 (C-27) ppm.

2.1.3. Experimental Procedure for the Synthesis of Betulin Analogs (3)

In a 10 mL round-bottom flask, 3-*O*-acetyl-28-*O′*-(3-carboxypropanoyl)betulin **2a** (292.4 mg, 0.5 mmol) or 28-*O′*-(3-carboxypropanoyl)betulin **2b** (271.4 mg, 0.5 mmol), potassium carbonate (450 mg, 3.28 mmol), and DMF (1.5 mL) were placed. Then, an 80% solution of propargyl bromide in toluene (0.15 mL, 188.8 mg, 1.75 mmol) was added under an argon atmosphere, in 0-5 ℃ and the reaction was allowed to proceed for 5 days at room temperature. Water (9 mL) was added to the reaction mixture. The solution was extracted with diethyl ether (**3a**: 4 × 5 mL; **3b**: 5x3 mL). The mixture was dried over anhydrous $MgSO_4$, the drying agent was filtered, and the filtrate was concentrated under diminished pressure.

3-O-Acetyl-28-O′-(3-propynylcarbonyloxypropanoyl)betulin (***3a***) was obtained as a resin, 96% yield; HRMS (ESI$^+$): calcd for $C_{39}H_{58}O_6$ ([M+Na]$^+$): *m*/*z* 645.4131 found: *m*/*z* 645.4139; ^{1}H NMR (600 MHz, $CDCl_3$) δ_H: 4.70 (d, 2H, *J* = 2.4 Hz, OCH_2C), 4.68 (s, br, 1H, H-29a), 4.59 (s, br, 1H, H-29b), 4.47 (dd, 1H, J_1 = 5.4 Hz, J_2 = 10.8 Hz, H-3), 4.29 (d, 1H, *J* = 10.8 Hz, H-28a), 3.88 (d, 1H, *J* = 10.8 Hz, H-28b), 2.71-2.66 (m, 4H, $O(CO)CH_2CH_2$), 2.48 (m, 1H, ≡CH), 2.42 (m, 1H, H-19), 2.04 (s, 3H, CH_3CO), 1.68 (s, 3H, CH_3-30), 1.85 - 0.8 (m, 24H, CH, CH_2, BN scaffold), 1.02, 0.97, 0.95, 0.85, 0.84 (all s, 15H, CH_3-23 - CH_3-27) ppm. ^{13}C NMR (150 MHz, $CDCl_3$) δ_c: 172.44 (CO), 171.61 (CO), 171.11 (CO), 150.19 (C-20), 110.02 (C-29), 81.04 (C-3), 77.59 ($OCH_2\underline{C}$≡), 75.13 (≡CH), 63.27 (C-28), 55.50 (C-5), 52.33 ($O\underline{C}H_2C$º), 50.40 (C-9), 48.92 (C-18), 47.84 (C-19), 46.54 (C-17), 42.82 (C-14), 41.02 (C-8), 38.51 (C-1), 37.92 (C-4), 37.71 (C-13), 37.19 (C-10), 34.64 (C-22), 34.24 (C-7), 29.84 (C-21), 29.69 (C-16), 29.24, 29.11 ($O(CO)\underline{C}H_2\underline{C}H_2$), 28.07 (C-23), 27.16 (C-15), 25.28 (C-12), 23.82 (C-2), 21.44 (C-11), 20.92 ($\underline{C}H_3CO$), 19.25 (C-30), 18.29 (C-6), 16.62 (C-25), 16.28 (C-26), 16.16 (C-24), 14.85 (C-27) ppm.

28-O′-(3-Propynylcarbonyloxypropanoyl)betulin (***3b***) was obtained as a resin, 75% yield; HRMS (ESI$^+$): calcd for $C_{37}H_{56}O_5$ ([M+Na]$^+$): *m*/*z* 60.4025; found: *m*/*z* 603.4012; ^{1}H NMR (600 MHz, $CDCl_3$) δ_H: 4.70 (d, 2H, *J* = 2.4 Hz, OCH_2C), 4.68 (s, br, 1H, H-29a), 4.58 (s, br, 1H, H-29b), 4.29 (d, 1H, *J* = 10.8 Hz, H-28a), 3.88 (d, 1H, *J* = 11.4 Hz, H-28b), 3.17 (dd, 1H, J_1 = 4.8 Hz, J_2 = 11.4 Hz, H-3), 2.70-2.66 (m, 4H, $O(CO)CH_2CH_2$), 2.50 (m, 1H, ≡CH), 2.43 (m, 1H, H-19), 1.68 (s, 3H, CH_3-30), 1.99 - 0.70 (m, 24H, CH, CH_2, BN scaffold), 1.02, 0.97, 0.96, 0.82, 0.76 (all s, 15H, CH_3-23 -CH_3-27) ppm. ^{13}C NMR (150 MHz, $CDCl_3$) δ_c: 172.33 (CO), 171.49 (CO), 150.08 (C-20), 109.87 (C-29), 78.85 (C-3), 76.89 ($OCH_2\underline{C}$≡), 75.07 (≡CH), 63.14 (C-28), 55.34 (C-5), 52.21 ($O\underline{C}H_2C$º), 50.39 (C-9), 48.84 (C-18), 47.71(C-19), 46.44 (C-17), 42.72 (C-14), 40.90 (C-8), 38.89 (C-1), 37.62 (C-4), 37.17 (C-13), 36.49 (C-10), 34.53 (C-22), 34.22 (C-7), 31.45 (C-21), 29.76 (C-16), 29.61, 29.14 ($O(CO)\underline{C}H_2\underline{C}H_2$), 29.00 (C-23), 28.03 (C-15), 27.41 (C-12), 27.07 (C-2), 25.23 (C-11), 20.72 (C-30), 18.35 (C-6), 16.12 (C-25), 16.05 (C-26), 15.43 (C-24), 14.80 (C-27) ppm.

2.2. Procedure for the Synthesis of Sugar Derivatives

2.2.1. Experimental Procedures for the Synthesis of Sugar 4a and 4c

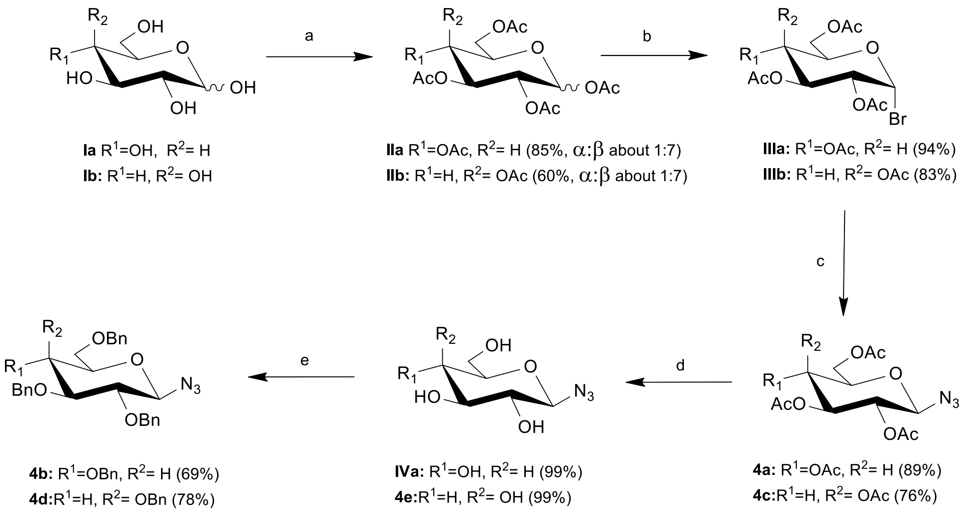

Scheme S1. Synthesis of sugar derivatives (**4a**-**4e**). Reagents and conditions: (**a**): AcONa, Ac_2O, b.p., 30 min; (**b**): 33% HBr/AcOH, AcOH, r.t. 24 h; (**c**): NaN_3, TBAHS, saturated solution $NaHCO_3$, $CHCl_3$, r.t., 18 h; (**d**): 1 M NaOMe in MeOH, MeOH, r.t. overnight; (**e**): BnBr, 60% NaH in mineral oil, DMF, 0 °C for 30 min and 20 °C overnight.

Anhydrous sodium acetate (5.74 g, 72.64 mmol) and acetic anhydride (56 mL, 598.77 mmol) placed in a round-bottomed flask fitted with a condenser was heated to boiling. Then glucose **Ia** or galactose **Ib** (10 g, 55.56 mmol) were added in small portions, maintaining the reaction mixture at boiling all the time (TLC, toluene/AcOEt, 1:1, v/v). After 30 minutes, the reaction mixture was poured into a beaker with an ice-water (600 mL) and the whole was stirred until the product had precipitated. The precipitate was filtered off under reduced pressure and dried giving 1,2,3,4,6-penta-*O*-acetyl-D-glucopyranose **IIa** (85% yield) or 1,2,3,4,6-penta-*O*-acetyl-D-galactopyranose **IIb** (60% yield). Compounds **IIa** and **IIb** were used for the next step without additional purification.

Per-*O*-acetyl-D-glucopyranose **IIa** or per-*O*-acetyl-D-glucopyranose **IIb** (3 g, 7.75 mmol) was dissolved in DCM (15 mL) and 33% solution of HBr in AcOH (6 mL) was added. The reaction was carried out at room temperature in argon environment without light for 24 hours (TLC, toluene/AcOEt, 1:1, v/v). Then the reaction mixture was diluted with DCM and washed with water (20 mL), brine (20 mL), saturated $NaHCO_3$ (25 mL), again brine (20 mL), and finally dried over anhydrous $MgSO_4$. After filtering off the drying agent, the filtrate was concentrated under reduced pressure. The crude product **IIIa** or **IIIb** was purified by column chromatography (toluene/AcOEt, gradient: 10:1 to 7:1, v/v).

*2,3,4,6-Tetra-O-acetyl-β-D-glucopyranosyl bromide **IIIa*** was obtained as a white powder, 94% yield. $[\alpha]_D^{20}$ = 181.4 (c = 1, $CHCl_3$), m.p. = 85-87 °C; ^{1}H NMR (400 MHz, $CDCl_3$) δ_H: 6.61 (d, 1H, J_1 = 4.0 Hz, H-1), 5.55 (dd~t, 1H, J_1 = 10.0 Hz, J_2 = 9.6 Hz, H-3), 5.19 (dd~t, 1H, J_1 = 10.0 Hz, J_2 = 9.6 Hz, H-4), 4.84 (dd, 1H, J_1 = 10.0 Hz, J_2 = 4.0 Hz, H-2), 4.35-4.30 (m, 2H, H-5, H-6b), 4.13 (m, 1H, H-6a), 2.10, 2.09, 2.05, 2.03 (4s, 12H, CH_3CO) ppm. ^{13}C NMR (100 MHz, $CDCl_3$) δ_C: 170.46, 169.81, 169.75, 169.42 (CO), 86.54 (C-1), 72.12, 70.59, 70.15, 67.17 (C-5, C-4, C-3, C-2), 60.94 (C-6), 20.64, 20.62, 20.59, 20.53 ($\underline{C}H_3CO$) ppm.

*2,3,4,6-Tetra-O-acetyl-β-D-glucopyranosyl bromide **IIIb*** was obtained as a white powder, 83% yield. $[\alpha]_D^{20}$ = 212.0 (c = 1, $CHCl_3$), m.p. = 84-86 °C; ^{1}H NMR (400 MHz, $CDCl_3$) δ_H: 6.70 (d, 1H, J_1 = 4.0 Hz, H-1), 5.52 (dd, 1H, J_1 =3.2 Hz, J_2 = 1.6 Hz, H-4), 5.40 (dd, J_1 = 10.8 Hz, J_2 = 3.2 Hz, 1H, H-3), 5.05 (dd, 1H, J_1 = 10.8 Hz, J_2 = 4.0 Hz, H-2), 4.48 (m, 1H, H-5), 4.21-4.08 (m, 2H, H-6a, H-6b), 2.15, 2.11, 2.06, 2.01 (4s, 12H, CH_3CO) ppm. ^{13}C NMR (100 MHz, $CDCl_3$) δ_C: 170.27, 170.02, 169.85, 169.71 (CO), 88.09 (C-1), 71.04, 67.97, 67.75, 66.96 (C-5, C-4, C-3, C-2), 60.80 (C-6), 20.71, 20.61, 20.55, 20.52 ($\underline{C}H_3CO$) ppm.

A 100 mL flask containing per-*O*-acetyl-D-glucopyranosyl bromide **IIIa** or per-*O*-acetyl-D-galactopyranosyl bromide **IIIb** (1.93 g, 4.69 mmol) was charged with chloroform (20 mL), followed by the addition of sodium azide (1.51g, 23.3 mmol), tetra-*n*-butylammonium hydrogen sulfate (1.58 g, 4.66 mmol), and saturated $NaHCO_3$ solution (20 mL). The mixture was stirred at room temperature for 18 h. The phases of reaction mixtures were separated and the aqueous phase was extracted with 30 mL of chloroform. The organic

phases were combined and washed with water (30 mL), saturated $NaHCO_3$ (30 mL), and brine (30 mL), and finally dried over anhydrous $MgSO_4$ and concentrated under reduced pressure. The crude product was purified by column chromatography (toluene/AcOEt, 10:1, v/v).

1-Azido-2,3,4,6-tetra-O-acetyl-β-D-glucopyranose ***4a*** was obtained as a white powder, 89% yield. $[\alpha]_D^{20}$ = -30.1 (c = 1, $CHCl_3$), m.p. = 125-127 °C; ^1H NMR (400 MHz, $CDCl_3$) δ_H: 5.21 (dd~t, 1H, J_1 = 9.4 Hz, J_2 = 9.8 Hz, H-3); 5.11 (dd~t, 1H, J_1 = 9.4 Hz, J_2 = 9.8 Hz, H-4), 4.96 (dd~t, 1H, J_1 = 9.0 Hz, J_2 = 9.8 Hz, H-2), 4.64 (d, 1H, J_1 = 9.0 Hz, H-1), 4.28 (dd, 1H, J_1 = 4.7 Hz, J_2 = 12.5 Hz, H-6b), 4.17 (dd, 1H, J_1 = 2.4 Hz, J_2 = 12.5 Hz, H-6a), 3.80 (ddd, 1H, J_1 = 2.4 Hz, J_2 = 4.7 Hz, J_3 = 9.8 Hz, H5), 2.11, 2.08, 2.04, 2.01 (4s, 12H, CH_3CO) ppm. ^{13}C NMR (100 MHz, $CDCl_3$) δ_C: 170.59, 170.10, 169.29, 169,19 (CO), 87.92 (C1β), 74.05, 72.62, 70.66, 67.90 (C2, C3, C4, C5), 61.67 (C6), 20.70, 20.56, 20.54, 20.53 ($\underline{C}H_3CO$) ppm.

1-Azido-2,3,4,6-tetra-O-acetyl-β-D-galactopyranose ***4c*** was obtained as a white powder, 76% yield. $[\alpha]_D^{20}$= -15 (c = 1, $CHCl_3$), m.p. = 96-98 °C; ^1H NMR (400 MHz, $CDCl_3$) δ_H: 5.43 (dd, 1H, J_1 = 1.2 Hz, J_2 = 3.5 Hz, H-4), 5.17 (dd, 1H, J_1 = 8.8 Hz, J_2 = 10.6 Hz, H-2), 5.04 (dd, 1H, J_1 = 3.5 Hz, J_2 = 10.6 Hz, H-3), 4.60 (d, 1H, J_1 = 8.8 Hz, H-1), 4.18-4.15 (m, 2H, H-6a, H-6b), 4.02 (m, 1H, H-5), 2.17, 2.09, 2.06, 1.99 (4s, 12H, CH_3CO). ^{13}C NMR (100 MHz, $CDCl_3$) δ_C: 170.35, 170.09, 169.97, 169.34 (CO), 88.29 (C-1), 72.86, 70.71, 68.04, 66.83 (C-2, C-3, C-4, C-5), 61.20 (C-6), 20.65, 20.63, 20.59, 20.50 ($\underline{C}H_3CO$) ppm.

2.2.2. Experimental Procedures for the Synthesis of Sugar 4b and 4d

1-Azido-2,3,4,6-tetra-*O*-acetyl-β-D-glucopyranose **4a** or 1-azido-2,3,4,6-tetra-*O*-acetyl-β-D-galactopyranose **4c** (2 g, 5.36 mmol) was dissolved in methanol (20 mL) and 1 M NaOMe in MeOH (5.36 mL, 5.36 mmol) was added. Reaction was stirred at r.t. overnight, neutralized to pH = 5 with *Amberlyst-15*, filtered through plug of *Celite* and condensed to give **IVa** or **4e** as white solid which was pure and was used for the next step without further purification.

1-Azido-β-D-glucopyranose ***IVa*** was obtained as a white powder, 99% yield. $[\alpha]_D^{20}$= -20.1 (c = 1, MeOH), m.p. = 86-89 °C; ^1H NMR (400 MHz, CD_3OD) δ_H: 4.49 (d, 1H, J_1 = 8.6 Hz, H-1), 3.88 (dd, 1H, J_1 = 2.1 Hz, J_2 = 12.1 Hz, H-6b), 3.68 (dd, 1H, J_1 = 5.5 Hz, J_2 = 12.1 Hz, H-6a), 3.37 (m, 1H, H-5), 3.36 (dd~t, 1H, J_1 = 8.5 Hz, J_2 = 8.8 Hz, H-3), 3.30 (dd~t, 1H, J_1 = 8.5 Hz, J_2 = 9.8 Hz, H-4), 3.14 (dd~t, 1H, J_1 = 8.6 Hz, J_2 = 8.8 Hz, H-2) ppm. ^{13}C NMR (100 MHz, CD_3OD) δ_C: 88.29 (C-1), 80.16, 78.10, 74.77, 71.13 (C-2, C-3, C-4, C-5), 62.55 (C-6) ppm.

1-Azido-β-D-galactopyranose ***4e*** was obtained as a white powder, 99% yield. $[\alpha]_D^{20}$= -6.1 (c = 1, MeOH), m.p.= 149-150 °C; ^1H NMR (400 MHz, CD_3OD) δ_H: 4.42 (d, 1H, J_1 = 8.4 Hz, H-1), 3.86 (dd, 1H, J_1 = 1.1 Hz, J_2 = 2.9 Hz, H-4), 3.78 (dd, 1H, J_1 = 6.9 Hz, J_2 = 11.4 Hz, H-6b), 3.72 (dd, 1H, J_1 = 5.1 Hz, J_2 = 12.1 Hz, H-6a), 3.61 (ddd, 1H, J_1 = 1.1 Hz, J_2 = 5.1 Hz, J_3 = 6.9 Hz, H-5), 3.46-3.52 (m, 2H, H-2, H-3) ppm. ^{13}C NMR (100 MHz, CD_3OD) δ_C: 92.63 (C-1), 78.95, 75.01, 72.04, 70.27 (C-2, C-3, C-4, C-5), 62.50 (C-6) ppm.

To β-D-galactopyranosyl azide **IVa** or β-D-galactopyranosyl azide **4e** (1.1 g, 5.36 mmol) in DMF (20 mL) benzyl bromide (2.61 mL, 21.98 mmol) was added drop wise at 0 °C. NaH 60% in mineral oil (0.97 g, 24.12 mmol) was added portion wise. Reaction mixture was stirred at 0 °C for 30 minutes and then at 20°C overnight. then the mixture was extracted by ethyl acetate (100 mL) and water (100 mL). Organic layer was washed with water (2 × 80 mL), dried over anhydrous $MgSO_4$, concentrated and purified by column chromatography (toluene/AcOEt, gradient 160:1 to 50:1 v/v).

1-Azido-2,3,4,6-tetra-O-benzyl-β-D-glucopyranose ***4b*** was obtained as a colourless oil, 69% yield. $[\alpha]_D^{20}$ = -0.1 (c = 1.4, $CHCl_3$); ^1H NMR (600 MHz, $CDCl_3$) δ_H: 7.37-7.12 (m, 20H, Ph), 4.89, 4.82 (qAB, 2H, J_1 = 11.1 Hz, OCH_2), 4.88, 4.75 (qAB, 1H, J_1 = 10.9 Hz, OCH_2), 4.62, 4.55 (qAB, 1H, J_1 = 12.3 Hz, OCH_2), 4.62 (d, 1H, J_1 = 8.6 Hz, H-1), 4.80, 4.55 (qAB, 2H, J_1 = 10.8 Hz, OCH_2), 3.75 (dd, 1H, J_1 = 2.0 Hz, J_2 = 11.0 Hz, H-6b), 3.71 (dd, 1H, J_1 = 4.3 Hz, J_2 = 11.0 Hz, H-6a), 3.68- 3.62 (m, 2H, H-2, H-4), 3.53 (ddd, 1H, J_1 = 2.1 Hz, J_2 = 4.3 Hz, J_3 = 9.5 Hz, H-5), 3.37 (dd~t, 1H, J_1 = 9.8 Hz, J_2 = 9.9 Hz, H-3) ppm. ^{13}C NMR (100 MHz, $CDCl_3$) δ_C: 138.33, 137.95, 137.89, 137.76 (C-Ph), 128.46, 128.42, 128.41, 128.18, 127.94, 127.93, 127.84, 127.82, 127.74, 127.70 (CH-Ph), 90.21 (C-1), 84.93, 81.74, 77.38, 77.13 (C-3, C-4, C-2, C-5), 75.72, 75.19, 75.11, 73.58 ($O\underline{C}H_2$-Ph), 68.44 (C-6) ppm.

1-Azido-2,3,4,6-tetra-O-benzyl-β-D-galactopyranoset ***4d*** was obtained as a colourless oil, 78% yield. $[\alpha]_D^{20}$ = -0.1 (c = 1.4, $CHCl_3$); ^1H NMR (600 MHz, $CDCl_3$) δ_H: 7.36-7.22 (m, 20H, Ph), 4.84, 4.80 (qAB, 2H, J_1 = 10.7 Hz, OCH_2), 4.70 (s, 2H, OCH_2), 4.92, 4.59 (qAB, 1H, J_1 = 11.6 Hz, OCH_2), 4.58 (d, 1H, J_1 = 8.5 Hz, H-1), 4.46, 4.41 (qAB, 1H, J_1 = 11.8 Hz, OCH_2), 3.75 (dd, 1H, J_1 = 2.0 Hz, J_2 = 11.0 Hz, H-6b), 3.92 (dd, 1H, J_1 = 0.5 Hz, J_2 = 2.7 Hz, H-4), 3.75 (dd, 1H, J_1 = 8.5 Hz, J_2 = 9.6 Hz, H-2), 3.62 (ddd, 1H, J_1 = 0.5 Hz, J_2 = 4.8 Hz, J_3 = 5.4 Hz, H-5), 3.59 (dd, 1H, J_1 = 5.4 Hz, J_2 = 13.4 Hz, H-6b),), 3.58 (dd, 1H, J_1 = 4.8 Hz, J_2 = 13.4 Hz, H-6a), 3.53 (dd, 1H, J_1 = 2.7 Hz, J_2 = 9.6 Hz, H-3) ppm. ^{13}C NMR (100 MHz, $CDCl_3$) δ_C: 137.92, 137.60, 137.54, 137.21 (C-Ph), 127.95, 127.93, 127.87,

127.74, 127.72, 127.58, 127.43, 127.36, 127.30, 127.22, 127.12, 127.04 (CH-Ph), 90.06 (C-1), 82.01, 78.25, 75.06, 74.99 (C-3, C-4, C-2, C-5), 74.10, 73.12, 72.75, 72.35 (O<u>C</u>H2-Ph), 67.96 (C-6) ppm.

2.2.3. Experimental Procedures for the Synthesis of Sugar 4f and 4g

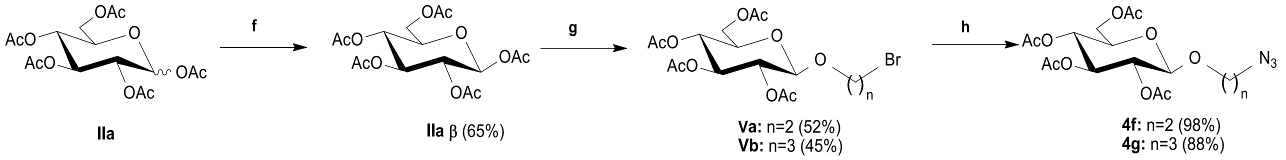

Scheme S2. Synthesis of sugar derivatives **4f-4g**. Reagents and conditions: (**f**): MeOH, b.p.; (**g**): 2-bromoetanol or 2-bromopropanol), $BF_3{\cdot}Et_2O$, DCM, 0°C for 30 min, r.t. 20 h; (**h**): NaN_3 , DMF, r.t. 24 h.

2-Bromoethanol (2.82 mmol) or 2-bromopropanol (2.82 mmol) was added to per-*O*-acetyl-β-D-glucopyranose **IIa** (1 g, 2.57 mmol) dissolved in DCM (7 mL). The reaction vessel was placed on a magnetic stirrer in an ice water bath and $BF_3{\cdot}Et_2O$ (322 μL, 2.56 mmol) was added. After 20 minutes, the flask was removed from the water bath and left at r.t. overnight. The reaction progress was monitored by TLC (toluene/AcOEt, 1:1, v/v). Then the reaction mixture was diluted with DCM (30 mL) and washed with brine (10 mL), saturated $NaHCO_3$ (10 mL), again brine (20 mL), and finally dried over anhydrous $MgSO_4$. After filtering off the drying agent, the filtrate was concentrated under reduced pressure. The crude product **Va** or **Vb** was purified by column chromatography (toluene/AcOEt, gradient 10:1 to 4:1, v/v).

*2-Bromoethyl 2,3,4,6-tetra-O-acetyl-β-D-glucopyranoside **Va*** was obtained as a white solid, 52% yield. $[\alpha]_D^{24}$ = 1.6 (c = 1, $CHCl_3$); m.p. = 103-110 °C; ^1H NMR (400 MHz, $CDCl_3$) δ_H: 5.22 (dd~t, 1 H, J_1 = 9.4 Hz, J_2 = 9.8 Hz, H-3), 5.09 (dd~t, 1H, J_1 = 9.8 Hz, J_2 = 10.0 Hz, H-4), 5.02 (dd, 1H, J_1 = 7.8 Hz, J_2 = 9.4 Hz, H-2), 4.58 (d, 1H, J_1 = 7.8 Hz, H-1), 4.26 (dd, 1H, J_1 = 4.7 Hz, J_2 = 12.2 Hz, H-6b), 4.16 (m, 1H, CH_2O), 4.15 (dd, 1H, J_1 = 2.3 Hz, J_2 = 12.2 Hz, H-6a), 3.82 (m, 1H, CH_2O), 3.71 (ddd, 1H, J_1 = 2.3 Hz, J=4.7 Hz, J_2 = 10.0 Hz, H-5), 3.50-3.42, (m, 2H, CH_2Br), 2.01, 2.03, 2.07, 2.09 (4s, 12H, CH_3CO) ppm. ^{13}C NMR (100 MHz, $CDCl_3$) δ_C: 170.60, 170.21, 169.36 (CH3<u>C</u>O), 101.01 (C-1), 72.61, 71.95, 71.04, 69.76 (C-5, C-3, C-2, C-4), 68.34 (CH_2O), 61.85 (C-6), 29.81 (CH_2Br), 20.71, 20.58, 20.56 (<u>C</u>H3CO) ppm.

*2-Bromopropyl 2,3,4,6-tetra-O-acetyl-β-D-glucopyranoside **Vb*** was obtained as a glassy solid, 45% yield. $[\alpha]_D^{20}$ = -3.9 (c = 1.5, $CHCl_3$); ^1H NMR (400 MHz, $CDCl_3$) δ_H: 5.22 (dd~t, 1 H, J_1 = 9.4 Hz, J_2 = 9.8 Hz, H-3), 5.08 (dd~t, 1H, J_1 = 9.8 Hz, J_2 = 10.0 Hz, H-4), 4.99 (dd, 1H, J_1 = 7.8 Hz, J_2 = 9.4 Hz, H-2), 4.51 (d, 1H, J_1 = 7.8 Hz, H-1), 4.26 (dd, 1H, J_1 = 4.7 Hz, J_2 = 12.1 Hz, H-6b), 4.14 (dd, 1H, J_1 = 2.3 Hz, J_2 = 12.1 Hz, H-6a), 3.98 (m, 1H, CH_2O), 3.74-3.64 (m, 2H, H-5, CH_2O), 3.56-3.41 (m, 2H, CH_2Br), 2.21-2.11 (m, 2H, CH_2), 2.01, 2.03, 2.07, 2.09 (4s, 12H, CH_3CO) ppm. ^{13}C NMR (100 MHz, $CDCl_3$) δ_C: 170.65, 170.24, 169.39 (CH3<u>C</u>O), 101.08 (C-1), 72.76, 71.85, 71.29, 68.42 (C-5, C-3, C-2, C-4), 67.35 (CH_2O), 61.94 (C-6), 32.28 (CH_2), 30.07 (CH_2Br), 20.75, 20.70, 20.61, 20.60 (<u>C</u>H3CO) ppm.

A 50 mL round-bottom flask was charged with 2-bromoethyl 2,3,4,6-tetra-*O*-acetyl-β-D-glucopyranoside **Va** or 2-bromopropyl 2,3,4,6-tetra-*O*-acetyl-β-D-glucopyranoside **Vb** (1.35 mmol) in DMF (9 mL). Sodium azide (537 mg, 8.10 mmol) was then added to the flask, then stirred overnight at room temperature. The reaction progress was monitored by TLC (toluene/AcOEt, 1:1, v/v). DMF was evaporated under reduced pressure. Water (50 mL) and AcOEt (50 mL) were added to the residue, separated phases, and organic phase was washed with 0.5 M aqueous HCl solution (40 mL), and brine (40 mL). The organic phase was dried over anhydrous $MgSO_4$ and concentrated under reduced pressure and purified by column chromatography (toluene/AcOEt, gradient 10:1 to 8:1, v/v).

*1-O-(2-Azidoethyl)-2,3,4,6-tetra-O-acetyl-β-D-glucopyranose **4f*** was obtained as a white solid, 98% yield. $[\alpha]_D^{24}$ = -23.5 (c = 1, $CHCl_3$); m.p. = 103-105 °C; ^1H NMR (400 MHz, $CDCl_3$) δ_H: 5.22 (dd~t, 1H, J_1 = 9.4 Hz, J_2 = 9.4 Hz, H-3), 5.11 (dd~t, 1H, J_1 = 9.4 Hz, J_2 = 9.8 Hz, H-4), 5.03 (dd~t, 1H, J_1 = 8.0 Hz, J_2 = 9.4 Hz, H-2), 4.60 (d, 1H, J_1 = 8.0 Hz, H-1), 4.26 (dd, 1H, J_1 = 3.7 Hz, J_2 = 12.3 Hz, H-6b), 4.17 (dd, 1H, J_1 = 2.5 Hz, J_2 = 12.3 Hz, H-6a), 4.04 (m, 1H, CH_2O), 3.74-3.66 (m, 2H, CH_2O, H-5), 3.50 (m, 1H, CH_2N), 3.30 (m, 1H, CH_2N), 2.09, 2.06, 2.03, 2.01 (4s, 12H, CH_3CO) ppm. ^{13}C NMR (100 MHz, $CDCl_3$) δ_C: 170.73, 170.36, 169.48 (CH_3CO), 100.78 (C-1), 72.90, 72.06, 71.18, 68.64 (C-5, C-3, C-2, C-4), 68.02, 61.95 (C-6, CH_2O), 50.64 (CH_2N_3), 20.71, 20.70, 20.67, 20.65 (CH_3CO) ppm.

*1-O-(3-Azidoethylpropyl)-2,3,4,6-tetra-O-acetyl-β-D-glucopyranose **4g*** was obtained as a white solid, 88% yield. $[\alpha]_D^{24}$ = - 3.1 (c = 1, $CHCl_3$), m.p = 112 – 113 °C; ^1H NMR (400 MHz, $CDCl_3$) δ_H: 5.21 (dd~t, 1H, J_1 = 9.4 Hz, J_2 = 9.8 Hz, H-3), 5.08 (dd~t, 1H, J_1 = 9.4 Hz, J_2 = 9.8 Hz, H-4), 5.03 (dd~t, 1H, J_1 = 8.2 Hz, J_2 = 9.8 Hz, H-2), 4.51 (d, 1H, J_1 = 8.2 Hz, H-1), 4.26 (dd, 1H, J_1 = 4.7 Hz, J_2 = 12.3 Hz, H-6b), 4.17 (dd, 1H, J_1 = 2.4 Hz, J_2 = 12.3 Hz, H-6a), 3.95 (m, 1H, CH_2O), 3.70 (ddd, 1H, J_1 = 2.4 Hz, J_2 = 4.7 Hz, J_3 = 9.8 Hz, H-5), 3.61 (m, 1H, CH_2O), 3.38 (m, 2H,

CH_2N), 2.09, 2.05, 2.03, 2.01 (4s, 12H, CH_3CO), 1.85 (m, 2H, C<u>H</u>$_2$CH$_2$N) ppm. ^{13}C NMR (100 MHz, $CDCl_3$) δ_C: 170.65, 170.26, 169.40, 169.31 (CH_3CO), 100.84 (C-1), 72.81, 71.88, 71.30, 70.67, 68.43 (C-5, C-3, C-2, C-4), 66.48, 61.94 (C-6, CH_2O), 47.95 (CH_2N_3), 28.99 (OCH$_2$<u>C</u>H$_2$CH$_2$), 20.74, 20.66, 20.61. 20.60 (CH_3CO) ppm.

2.2.4. Experimental Procedures for the Synthesis of Sugar 4h

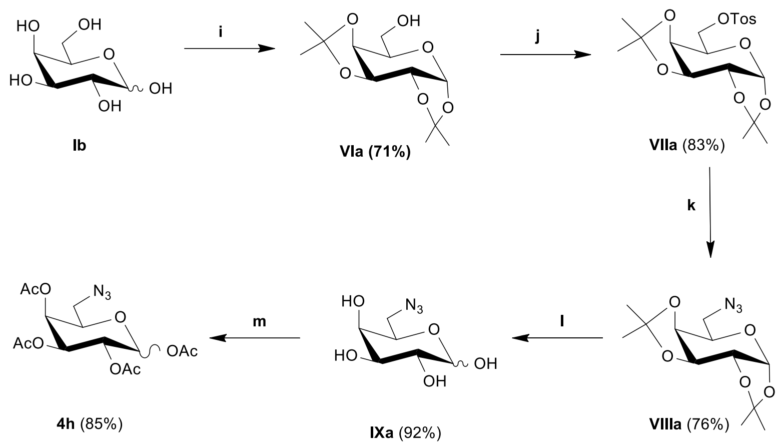

Scheme S3. Synthesis of sugar derivative **4h**. Reagents and conditions: (**i**): I_2, r.t., acetone, 20 h; (**j**): DMAP, TosCl, pyridine, r.t. 24 h; (**k**): NaN_3, DMF, 100°C. 48 h; (**l**): Amberlyst-15, pH~2, MeOH:H_2O (3:2, v/v), 70 ^{0}C, 24 h; (**m**): AcONa, Ac_2O, b.p., 3 h.

To a solution of D-galactose **Ib** (2 g, 11.09 mmol) in dry acetone (100 mL), iodine (0.590 g, 2.33 mmol) was added. The reaction mixture was stirred at room temperature for 20 h. The reaction progress was monitored by TLC (toluene/AcOEt, 1:1, v/v). After the completion reaction, 10% $Na_2S_2O_3$ aqueous solution (15 mL) was added to the reaction mixture and then the solvent was evaporated under reduced pressure. The residue was diluted with dichloromethane (100 mL) and washed twice with brine (40 mL). The organic layer was dried over anhydrous $MgSO_4$, and after filtering off the drying agent, the filtrate was concentrated under reduced pressure and purified by column chromatography (toluene/AcOEt, gradient 40:1 to 2:1, v/v).

1,2:3,4-Di-O-isopropylidene-α-D-galactopyranose ***VIa*** was obtained as a yellow oil, 71% yield. $[\alpha]_D^{24}$ = -50.4 (c = 1, $CHCl_3$); ^{1}H NMR (400 MHz, $CDCl_3$) δ_H: 5.57 (d, 1H, J_1 = 5.0 Hz, H-1), 4.62 (dd, 1H, J_1 = 2.4 Hz, $_2J$ = 7.9 Hz, H-3), 4.34 (dd, 1H, J_1 = 2.4 Hz, J_2 = 5.0 Hz, H-2), 4.28 (dd, 1H, J_1 = 1.7 Hz, $_2J$ = 7.9 Hz, H-4), 3.91–3.83 (m, 2H, H-6a, H-6b), 3.75 (m, 1H, H-5), 1.34, 1.34, 1.46, 1.54 (4s, 12H, CH_3) ppm. ^{13}C NMR (100 MHz, $CDCl_3$) δ_C: 109.62, 108.82 ((CH_3)$_2$<u>C</u>), 96.44 (C-1), 71.76, 70.91, 70.73, 68.23 (C2, C3, C4, C5), 62.49 (C6), 26.18, 26.07, 25.08, 24.45 (CH_3) ppm.

To a solution of 1,2:3,4-di-*O*-isopropylidene-*α*-D-galactopyranose **VIa** (1 g, 3.84 mmol) in pyridine (10 mL), *p*-toluenosulfonyl chloride (1.80 g, 9.42 mmol) and DMAP (0.09 g, 0.741 mmol) were added. The reaction mixture was stirred at room temperature for 24 h. After completion, the reaction was diluted with water (60 mL) and washed with chloroform (3 × 80 mL), the organic layer was dried over anhydrous $MgSO_4$, filtered off, and the solvent was concentrated under vacuum. The crude product was purified by column chromatography (toluene/AcOEt, gradient 100:1 to 5:1, v/v).

1,2:3,4-Di-O-isopropylidene-6-O-p-toluenesulfonyl-α-D-galactopyranose ***VIIa*** was obtained as a yellow oil, 83% yield. $[\alpha]_D^{24}$ = -24.1 (c = 1, $CHCl_3$); ^{1}H NMR (400 MHz, $CDCl_3$) δ_H: 7.81 (d, 2H, J_1 = 8.2 Hz, Ph), 7.33 (d, 2H, J_1 = 8.2 Hz, Ph), 5.46 (d, 1H, J_1 = 5.0 Hz, H-1), 4.59 (dd, 1H, J_1 = 2.5 Hz, $_2J$ = 7.9 Hz, H-3), 4.29 (dd, 1H, J_1 = 2.5 Hz, J_2 = 5.0 Hz, H-2), 4.23–4.17 (m, 2H, H-4, H-5), 4.13–4.02 (m, 2H, H-6a, H-6b), 2.44 (s, 3H, C<u>H</u>$_3$Ph), 1.50, 1.35, 1.32, 1.28 (4s, 12H, CH_3) ppm. ^{13}C NMR (100 MHz, $CDCl_3$) δ_C: 144.75, 132.84, 129.74, 128.12 (C-Ph), 109.59, 108.95 ((CH_3)$_2$<u>C</u>), 96.13 (C-1), 70.53, 70.42, 70.38, 68.20 (C-2, C-3, C-4, C-5), 65.88 (C-6), 25.99, 25.82, 24.92, 24.36 (CH_3), 21.63 (<u>C</u>H$_3$-Ph) ppm.

To a solution of 1,2,3,4-di-O-isopropylidene-6-*O*-p-toluenesulfonyl-*α*-D-galactopyranose **VIIa** (1.32 g, 3.18 mmol) in dry DMF (10 mL), sodium azide (0.84 g, 12.88 mmol) was added. The reaction mixture was stirred at 100 ^{0}C for 48 h. The reaction progress was monitored by TLC (toluene/AcOEt, 1:1, v/v). After the completion reaction, the reaction mixture was diluted with chloroform (80 mL) and water (50 mL). The aqueous phase was washed with chloroform (2 × 50 mL), and the combined organic phases were washed with brine (2 × 50 mL), the organic layer was dried over anhydrous $MgSO_4$, filtered off, and the solvent was concentrated under vacuum. The crude product was purified by column chromatography (toluene/ethyl acetate, gradient 30:1 to 10:1, v/v).

1,2:3,4-Di-O-isopropylidene-6-azido-6-deoxy-α-D-galactopyranose ***VIIIa*** was obtained as coloreless oil, 76% yield. $[\alpha]_D^{24}$ = -104.1 (c = 1, $CHCl_3$); ^{1}H NMR (400 MHz, $CDCl_3$) δ_H: 5.55 (d, 1H, J_1 = 5.0 Hz, H-1), 4.63 (dd, 1H, J_1 = 2.5 Hz, $_2J$ = 7.9 Hz, H-3), 4.34 (dd, 1H, J_1 = 2.5 Hz, J_2 = 5.0 Hz, H-2), 4.20 (dd, 1H, J_1 = 2.0 Hz, J_2 = 7.9 Hz, H-4), 3.92 (ddd, 1H, J_1 = 2.0 Hz, J_2 = 5.4 Hz, J_3 = 7.8 Hz, H-5), 3.52 (dd, 1H, J_1 = 7.8 Hz, J_2 = 12.7 Hz, H6b), 3.37 (dd, 1H, J_1 = 5.4 Hz, J_2 = 12.7 Hz, H6a), 1.55, 1.46, 1.35, 1.34 (4s, 12H, CH_3) ppm. ^{13}C NMR (100 MHz, $CDCl_3$) δ_C: 109.63, 108.81 ((CH_3)$_2$C), 96.35 (C-1), 71.17, 70.81, 70.40, 67.00 (C-2, C-3, C-4, C-5), 50.68 (C-6), 26.03, 25.95, 24.89, 24.44 (CH_3), 21.63 (CH_3-Ph) ppm.

To a solution of 1,2:3,4-di-O-isopropylidene-6-azido-6-deoxy-α-D-galactopyranose **VIIIa** (0.69 g, 2.41 mmol) in MeOH/H_2O (3:2, v/v) solution (15 mL) *Amberlyst-15* was added until pH = 2. The resulting mixture was heated at 70 °C for 24 h (TLC, $CHCl_3$/MeOH, 5:1, v/v). Then the cation resin was filtered off and washed with MeOH (3 × 10 mL). The combined filtrates were neutralized with 1 M MeONa in MeOH and concentrated under reduced pressure. The crude product was purified by column chromatography ($CHCl_3$/MeOH, gradient 40:1 to 11:1, v/v).

6-Azido-6-deoxy-D-galactopyranose ***IXa*** was obtained as a white solid, 92% yield, ratio of anomers (α:β = 6.3:1). ^{1}H NMR (400 MHz, DMSO-d6) δ_H: 6.62 (d, 1H, J_1 = 6.9 Hz, H-1$_\beta$), 6.29 (d, 1H, J_1 = 4.8 Hz, H-1$_\alpha$), 4.95 (t, 1H, J_1 = 4.1 Hz, OH$_\alpha$), 4.75 (d, 1H, J_1 = 4.3 Hz, OH$_\beta$), 4.71 (d, 1H, J_1 = 5.2 Hz, OH$_\beta$), 4.58 (d, 1H, J_1 = 4.5 Hz, OH$_\beta$), 4.54 (d, 1H, J_1 = 6.7 Hz, OH$_\alpha$), 4.53 (d, 1H, J_1 = 5.2 Hz, OH$_\alpha$), 4.33 (d, 1H, J_1 = 6.6 Hz, OHα), 4.26 (t, 1H, J_1 = 7.1 Hz, OH$_\beta$), 4.09 (dd, 1H, J_1 = 5.2 Hz, J_2 = 10.5 Hz, H-4$_\beta$), 3.95 (dd, 1H, J_1 = 4.2 Hz, J_2 = 8.6 Hz, H-4$_\alpha$), 3.68 (m, 1H, H-5$_\beta$), 3.61 (m, 1H, H-5$_\alpha$), 3.59–3.48 (m, 4H, H-2$_\alpha$, H-2$_\beta$, H-3$_\alpha$, H-3$_\beta$), 3.47–3.37 (m, 2H, H-6b$_\alpha$, H-6b$_\beta$), 3.28 (dd, 1H, J_1 = 4.3 Hz, J_2 = 12.7 Hz, H-6a$_\alpha$), 3.17 (m, 1H, H-6a$_\beta$) ppm. ^{13}C NMR (100 MHz, DMSO-d6) δ_C: 97.42(C-1$_\beta$), 92.71 (C-1$_\alpha$), 73.08, 72.97, 71.74, 71.72 (C-$_{2\beta}$, C-3$_\beta$, C-4$_\beta$, C-5$_\beta$), 69.60, 68.97, 68.83, 68.40(C-2$_\alpha$, C-3$_\alpha$, C-4$_\alpha$, C-5$_\alpha$), 51.46, 51.36 (C-6$_\alpha$, C-6$_\beta$) ppm.

In a 25 mL round-bottom flask**,** 6-azido-6-deoxy-D-galactopyranose **IXa (**0.29 g, 1.42 mmol) was dissolved in 8 mL of Ac_2O. Then, AcONa (0.09 g, 1.10 mmol) was added, and the mixture was heated to reflux under a condenser for 3 hours with continuous stirring, monitored by TLC analysis: toluene/AcOEt (5:1, v/v); $CHCl_3$/MeOH (5:1, v/v). The reaction mixture was poured into ice water and extracted with DCM (3 × 30 mL), dried over anhydrous $MgSO_4$ and concentrated under reduced pressure.

Product ***4h*** was obtained as a yellow oil, 85% yield, ratio of anomers (α:β = 1:3.5). ^{1}H NMR (600 MHz, $CHCl_3$) δ_H: 6.33 (d, 1H, J_1 = 4.8 Hz, H-1$_\alpha$), 5.71 (d, 1H, J_1 = 8.4 Hz, H-1$_\beta$), 5.49 (m, 1H, H-4$_\alpha$), 5.42 (dd, 1H, J_1 = 1.0 Hz, J_2 = 3.6 Hz, H-4$_\beta$), 5.37-5.33 (m, 3H, H3$_\alpha$, H-2$_\alpha$, H-2$_\beta$), 5.08 (dd, 1H, J_1 = 3.6 Hz, J_2 = 10.8 Hz, H-3$_\beta$), 4.21 (m, 1H, H-5$_\alpha$), 3.94 (m, 1H, H-5$_\beta$), 3.55-3.50 (m, 2H, H-6a$_\alpha$, H-6a$_\alpha$), 3.45 (dd, 1H, J_1 = 7.4 Hz, J_2 = 12.8 Hz, H-6b$_\alpha$), 3.22 (dd, 1H, J_1 = 5.5 Hz, J_2 = 12.8 Hz, H-6b$_\beta$), 2.18, 2.17, 2.03, 2.01 (4s, 12H, CH_3CO_α), 2.23, 2.19, 2.05, 2.00 (4s, 12H, CH_3CO_β) ppm. ^{13}C NMR (150 MHz, $CHCl_3$) δ_C: 170.05, 169.78, 169.25, 168.84 (CO$_\alpha$), 170.04, 169.87, 169.37, 168.86 (CO$_\beta$), 92.10 (C-1$_\beta$), 89.60 (C-1$_\alpha$), 73.34, 70.10, 67.34, 66.32 (C-2$_\alpha$, C-3$_\alpha$, C-4$_\alpha$, C-5$_\alpha$), 73.18, 70.80, 67.65, 67.46 (C-2$_\beta$, C-3$_\beta$, C-4$_\beta$, C-5$_\beta$), 50.28 (C-N$_{3\alpha}$), 50.05 (C-N$_{3\beta}$), 21.01, 20.85, 20.70, 20.42 (CH_3CO_α), 20.73, 20.61, 20.60, 20.49 (CH_3CO_β) ppm.

2.3. General Procedure for the Synthesis of Betulin Glycoconjugates (5 – 8)

In a 25 mL round-bottom flask, 3-*O*-acetyl-28-*O'*-(propynylcarbonyloxypropanoyl) betulin **3a** or 28-*O'*-(propynylcarbonyloxypropanoyl) betulin **3b** (0.11 mmol) was combined with the selected sugar derivative **4a – h** (0.11 mmol), isopropyl alcohol (9 mL), and tetrahydrofuran (9 mL). The mixture was stirred at room temperature under an argon atmosphere, and then solutions of $CuSO_4$·$5H_2O$ (16 mg; 0.0641 mmol) in 4.5 mL of water and sodium ascorbate (24 mg; 0.136 mmol) in 4.5 mL of water were added. The reaction was allowed to proceed under these conditions for 7 days. monitored by thin-layer chromatography (DCM/MeOH 50:1, v/v). Then the reaction mixture was extracted with DCM (2×30 mL), organic layers were combined and dried over anhydrous $MgSO_4$, the drying agent was filtered off, and the solvent was evaporated under diminished pressure. The crude residue was purified using column chromatography (**5a, 5c, 6a, 6b**: DCM/MeOH, gradient: 50:1; **5b, 7d**: PhMe/AcOEt 10:1; **7a, 7c:** PhMe/AcOEt, gradient: 10:1 to 0:1; **7b**: PhMe/AcOEt, gradient: 5:1 to 0:1; **8a**: DCM/MeOH gradient 50:1 to 20:1). The product content in the fractions was monitored by TLC (DCM/MeOH 50:1 or 20:1; PhMe/AcOEt 2:1 v/v).

Product ***5a*** *(3-O-Ac-28-O'-SAOCH$_2$TriGlu(OAc)BN)* was obtained as a white solid, 84% yield; $[\alpha]^{28}_D$ = -56.7 (c=1, $CHCl_3$); HRMS (ESI$^+$): calcd for $C_{53}H_{77}N_3O_{15}$ ([M+Na]$^+$): *m/z* 1018.5252, found: *m/z* 1018.5353; ^{1}H NMR (600 MHz, $CDCl_3$) δ_H: 7.84 (s, br, 1H, H-5$_{triaz}$), 5.87 (m, 1H, H-1$_{glu}$), 5.44-5.38 (m, 2H, H-3$_{glu}$, H-2$_{glu}$), 5.26-5.21 (m, 3H, H-4$_{glu}$, OCH_2), 4.68 (s, br, 1H, H-29a), 4.59 (s, br, 1H, H-29b), 4.47 (dd, 1H, J_1 = 10.8 Hz, J_2 = 5.4 Hz, H-3), 4.31 (dd, 1H, J_1 = 4.9 Hz, J_2 = 12.7 Hz, H-6a$_{glu}$), 4.29 (d, 1H, J = 10.8 Hz, H-28a), 4.15 (dd, 1H, J_1 = 2.1 Hz, J_2 = 12.7 Hz,

H-6b$_{glu}$), 4.00 (ddd, 1H, J_1 = 2.1 Hz, J_2 = 4.9 Hz, J_3 = 10.1 Hz, H-5$_{glu}$), 3.88 (d, 1H, J = 11.4 Hz, H-28b), 2.70-2.63 (m, 4H, O(CO)CH_2CH_2), 2.43 (m, 1H, H-19), 2.09, 2.07, 2.04, 2.03, 1.88 (all s, 3H each, 5 x CH_3CO), 1.68 (s, 3H, CH_3-30), 2.00 - 0.72 (m, 24H, CH, CH_2, BN scaffold), 1.03, 0.97, 0.85, 0.84, 0.83 (all s, 15H, CH_3-23 - CH_3-27) ppm. ^{13}C NMR (150 MHz, $CDCl_3$) δ_C: 172.54 (CO), 172.13 (CO), 171.09 (CO), 170.55 (CO), 169.97 (CO), 169.41 (CO), 168.94 (CO), 150.19 (C-20), 143.66 (C$_{triaz}$), 122.22 (CH$_{triaz}$), 110.02 (C-29), 85.92 (C-1$_{glu}$), 81.05 (C-3), 75.37, 72.71, 70.49, 67.82 (C-5$_{glu}$, C-2$_{glu}$, C-3$_{glu}$, C-4$_{glu}$), 63.24 (C-28), 61.63 (C-6$_{glu}$), 57.78 (OCH_2), 55.52 (C-5), 50.42 (C-9), 48.94 (C-18), 47.85 (C-19), 46.56 (C-17), 42.83 (C-14), 41.03 (C-8), 38.52 (C-1), 37.93 (C-4), 37.73 (C-13), 37.20 (C-10), 34.64 (C-22), 34.26 (C-7), 29.86 (C-21), 29.71 (C-16), 29.29, 29.21 (O(CO)$\underline{C}H_2\underline{C}H_2$), 28.07 (C-23), 27.18 (C-15), 25.30 (C-12), 23.83 (C-2), 21.56 (C-11), 20.93, 20.79, 20.63, 20.25 ($\underline{C}H_3CO$), 19.25 (C-30), 18.30 (C-6), 16.61 (C-25), 16.28 (C-26), 16.17 (C-24), 14.86 (C-27) ppm.

Product ***5b*** *(3-O-Ac-28-O′-SAOCH$_2$TriGlu(OBn)BN)* was obtained as a resin, 60% yield; $[\alpha]^{27}_D$ = -0.6 (c=0.5, $CHCl_3$); HRMS (ESI$^+$): calcd for $C_{73}H_{93}N_3O_{11}$ ([M+Na]$^+$): *m/z* 1210.6708; found: *m/z* 1210.7081; ^{1}H NMR (600 MHz, $CDCl_3$) δ_H: 7.68 (s, br, 1H, H-5$_{triaz}$), 7.32-6.91 (m, 20H, Ph), 5.56 (d, 1H, J = 9.1 Hz, H-1$_{glu}$), 5.22 (qAB, 2H, J = 12.8 Hz, OCH_2), 4.91 (qAB, 2H, J = 11.1 Hz, OCH_2), 4.85 and 4.59 (qAB, 2H, J = 10.7 Hz, OCH_2), 4.68 (s, br, 1H, H-29a), 4.58 (s, br, 1H, H-29b), 4.55 and 4.49 (qAB, 2H, J = 12.1 Hz, OCH_2), 4.50 and 4.09 (qAB, 2H, J = 10.7 Hz, OCH_2), 4.49 (dd, 1H, J_1 = 4.8 Hz, J_2 = 10.2 Hz, H-3), 4.29 (d, 1H, J = 11.4 Hz, H-28a), 4.03 (dd~t, 1H, J_1 = 8.7 Hz, J_2 = 8.9 Hz, H-3$_{glu}$), 3.87 (d, 1H, J = 11.4 Hz, H-28b), 3.83 (dd~t, 1H, J_1 = 8.7 Hz, J_2 = 9.1 Hz, H-2$_{glu}$), 3.80 (dd~t, 1H, J_1 = 8.9 Hz, J_2 = 9.2 Hz, H-4$_{glu}$), 3.75-3.70 (m, 2H, H-6a$_{glu}$, H-6b$_{glu}$), 3.69 (ddd, 1H, J_1 = 2.1 Hz, J_2 = 4.0 Hz, J_3 = 9.2 Hz, H-5$_{glu}$), 2.64-2.58 (m, 4H, O(CO)CH_2CH_2), 2.42 (m, 1H, H-19), 1.68 (s, 3H, CH_3-30), 1.97 - 0.77 (m, 24H, CH, CH_2, BN scaffold), 1.02, 0.97, 0.96, 0.84, 0.83 (all s, 15H, CH_3-23 - CH_3-27) ppm. ^{13}C NMR (150 MHz, $CDCl_3$) δ_C: 172.48 (CO), 172.14 (CO), 171.06 (CO), 150.15 (C-20), 142.93 (C$_{triaz}$), 138.20 (C-Ph), 137.83 (C-Ph), 137.78 (C-Ph), 137.06 (C-Ph), 128.57(CH-Ph), 128.51(CH-Ph), 128.45(CH-Ph), 128.31(CH-Ph), 128.28 (CH-Ph), 128.07 (CH-Ph), 128.04 (CH-Ph), 127.93 (CH-Ph), 127.90(CH-Ph), 127.87 (CH-Ph), 127.78 (CH-Ph),123.30 (CH$_{triaz}$), 110.00 (C-29), 87.65 (C-1$_{glu}$), 85.56, 81.01, 80.76, 78.20, 75.85, 75.29, 74.99, 73.64 (C-5$_{glu}$, C-2$_{glu}$, C-3$_{glu}$, C-4$_{glu}$, OCH_2-Ph), 68.46 (C-6$_{glu}$), 63.18 (C-28), 57.91 (OCH_2), 55.48 (C-5), 50.38 (C-9), 48.90 (C-18), 47.80 (C-19), 46.53 (C-17), 42.79 (C-14), 40.99 (C-8), 38.49 (C-1), 37.89 (C-4), 37.69 (C-13), 37.16 (C-10), 34.62 (C-22), 34.22 (C-7), 29.83 (C-21), 29.68 (C-16), 29.23, 29.16 (O(CO)$\underline{C}H_2\underline{C}H_2$), 28.05 (C-23), 27.15 (C-15), 25.27 (C-12), 23.80 (C-2), 21.40 (C-11), 20.90 ($\underline{C}H_3CO$), 19.24 (C-30), 18.27 (C-6), 16.59 (C-25), 16.26 (C-26), 16.15 (C-24), 14.84 (C-27) ppm.

Product ***5c*** *(3-OH-28-O′-SAOCH$_2$TriGlu(OAc)BN)* was obtained as a white solid, 53% yield. $[\alpha]^{27}_D$ = -9.9 (c=1, $CHCl_3$); HRMS (ESI$^+$): calcd for $C_{51}H_{75}N_3O_{14}$ ([M+Na]$^+$): *m/z* 976.5147; found: *m/z* 976.5180; ^{1}H NMR (600 MHz, $CDCl_3$) δ_H: 7.85 (s, br, 1H, H-5$_{triaz}$), 5.89 (m, 1H, H-1$_{glu}$), 5.46-5.39 (m, 2H, H-3$_{glu}$, H-2$_{glu}$), 5.24 (dd~t, J_1 = 9.2 Hz, J_2 = 10.1 Hz, 1H, H-4$_{glu}$), 5.24 (qAB, 2H, J = 12.9 Hz, OCH_2), 4.69 (s, br, 1H, H-29a), 4.59 (s, br, 1H, H-29b), 4.31 (dd, 1H, J_1 = 4.9 Hz, J_2 = 12.7 Hz, H-6a$_{glu}$), 4.29 (d, 1H, J = 11.4 Hz, H-28a), 4.16 (dd, 1H, J_1 = 2.1 Hz, J_2 = 12.7 Hz, H-6b$_{glu}$), 4.02 (ddd, 1H, J_1 = 2.1 Hz, J_2 = 4.9 Hz, J_3 = 10.1 Hz, H-5$_{glu}$), 3.89 (d, 1H, J = 11.4 Hz, H-28b), 3.18 (dd, 1H, J_1 = 4.8 Hz, J_2 = 11.4 Hz, H-3), 2.69-2.65 (m, 4H, O(CO)CH_2CH_2), 2.43 (m, 1H, H-19), 2.09, 2.07, 2.03, 1.88 (all s, 3H each, 4 x CH_3CO), 1.68 (s, 3H, CH_3-30), 2.00 - 0.68 (m, 24H, CH, CH_2, BN scaffold), 1.03, 0.98, 0.97, 0.82, 0.76 (all s, 15H, CH_3-23 - CH_3-27) ppm;^{13}C NMR (150 MHz, $CDCl_3$) δ_C: 172.49 (CO), 172.09 (CO), 170.52 (CO), 169.94 (CO), 169.38 (CO), 168.90 (CO), 150.15 (C-20), 143.59 (C$_{triaz}$), 122.22 (CH$_{triaz}$), 109.94 (C-29), 85.84 (C-1$_{glu}$), 78.99 (C-3), 75.29, 72.67, 70.45, 67.77 (C-5$_{glu}$, C-2$_{glu}$, C-3$_{glu}$, C-4$_{glu}$), 63.18 (C-28), 61.60 (C-6$_{glu}$), 57.73 (OCH_2), 55.39 (C-5), 50.46 (C-9), 48.90 (C-18), 47.77 (C-19), 46.52 (C-17), 42.79 (C-14), 40.97 (C-8), 38.95 (C-4), 38.81 (C-1), 37.69 (C-13), 37.24 (C-10), 34.59 (C-22), 34.28 (C-7), 29.83 (C-21), 29.67 (C-16), 29.25, 29.16 (O(CO)$\underline{C}H_2\underline{C}H_2$), 28.08 (C-23), 27.48 (C-15), 27.14 (C-12), 25.30 (C-2), 21.84 (C-11), 20.87, 20.75, 20.57, 20.20 ($\underline{C}H_3CO$), 19.22 (C-30), 18.37 (C-6), 16.17 (C-25), 16.12 (C-26), 15.46 (C-24), 14.86 (C-27) ppm.

Product ***6a*** *(3-O-Ac-28-O′-SAOCH$_2$TriGal(OAc)BN)* was obtained as a white solid, 69% yield. $[\alpha]^{26}_D$ = 4.6 (c=1, $CHCl_3$); HRMS (ESI$^+$): calcd for $C_{53}H_{75}N_3O_{15}$ ([M+Na]$^+$): *m/z* 1018.5252; found: *m/z* 1018.5360; ^{1}H NMR (600 MHz, $CDCl_3$) δ_H: 7.91 (s, br, 1H, H-5$_{triaz}$), 5.85 (d, 1H, J = 9.3 Hz, H-1$_{gal}$), 5.56 (dd, 1H, J_1 = 1.0 Hz, J_2 = 3.4 Hz, H-4$_{gal}$), 5.53 (dd~t, 1H, J_1 = 9.4 Hz, J_2 = 10.2 Hz, H-2$_{gal}$), 5.26 (dd, 1H, J_1 = 3.4 Hz, J_2 = 10.2 Hz, H-3$_{gal}$), 5.22-5.28 (m, 2H, OCH_2), 4.68 (s, br, 1H, H-29a), 4.58 (s, br, 1H, H-29b), 4.48 (dd, 1H, J_1 = 5.4 Hz, J_2 = 10.8 Hz, H-3), 4.30 (d, 1H, J = 10.8 Hz, H-28a), 4.25 (ddd, 1H, J_1 = 1.0 Hz, J_2 = 5.9 Hz, J_3 = 6.8 Hz, H-5$_{gal}$), 4.21 (dd, 1H, J_1 = 5.9 Hz, J_2 = 11.4 Hz, H-6a$_{gal}$), 4.15 (dd, 1H, J_1 = 5.8 Hz, J_2 = 11.4 Hz, H-6b$_{gal}$), 3.89 (d, 1H, J = 10.8 Hz, H-28b), 2.70-2.65 (m, 4H, O(CO)CH_2CH_2), 2.45-2.41 (m, 1H, H-19), 2.24, 2.05, 2.04, 2.01, 1.89 (all s, 3H each, 5 x CH_3CO), 1.68 (s, 3H, CH_3-30), 1.82 - 0.78 (m, 24H, CH, CH_2, BN scaffold), 1.03, 0.97, 0.85, 0.84, 0.83 (all s, 15H, CH_3-23 - CH_3-27) ppm. ^{13}C NMR (150 MHz, $CDCl_3$) δ_C: 172.50 (CO), 172.14 (CO), 171.05 (CO), 170.35 (CO), 170.03 (CO), 169.82 (CO), 169.07 (CO), 150.12 (C-20), 143.48 (C$_{triaz}$), 122.35 (CH$_{triaz}$), 109.98 (C-29), 86.37 (C-1$_{gal}$), 80.97 (C-3), 74.19, 70.78, 68.01, 66.92 (C-5$_{gal}$, C-2$_{gal}$, C-3$_{gal}$, C-4$_{gal}$), 63.18 (C-28), 61.26, 57.72 (C-6$_{gal}$,OCH_2), 55.44 (C-5), 50.34 (C-9), 48.86

(C-18), 47.79 (C-19), 46.50 (C-17), 42.76 (C-14), 40.96 (C-8), 38.45 (C-1), 37.86 (C-4), 37.66 (C-13), 37.13 (C-10), 34.57 (C-22), 34.18 (C-7), 29.79 (C-21), 29.63 (C-16), 29.24, 29.17 (O(CO)$\underline{C}H_2\underline{C}H_2$), 28.01 (C-23), 27.11(C-15), 25.22 (C-12), 23.76 (C-2), 21.38 (C-11) , 20.86, 20.75, 20.71, 20.55, 20.30 ($\underline{C}H_3$CO), 19.19 (C-30), 18.24 (C-6), 16.56 (C-25), 16.23 (C-26), 16.10 (C-24), 14.80 (C-27) ppm.

Product ***6b*** *(3-O-Ac-28-O'-SAOCH$_2$TriGal(OBn)BN)* was obtained as a resin, 53% yield. $[\alpha]^{27}_{D}$ = -4.4 (c=1, $CHCl_3$); HRMS (ESI$^+$): calcd for $C_{73}H_{93}N_3O_{11}$ ([M+Na]$^+$): *m/z* 1210.6708; found: *m/z* 1210.6998; ^{1}H NMR (600 MHz, $CDCl_3$) δ_H: 7.64 (s, br, 1H, H-5$_{triaz}$), 7.37-6.94 (m, 20H, Ph), 5.58 (d, 1H, *J* = 9.0 Hz, H-1$_{gal}$), 5.16 i 5.19 (qAB, 2H, *J* = 12.8 Hz, OCH_2), 4.98 i 4.64 (qAB, 2H, *J* = 11.4 Hz, OCH_2), 4.77 and 4.72 (qAB, 2H, *J* = 11.8 Hz, OCH_2), 4.68 (s, br, 1H, H-29a), 4.58 (s, br, 1H, H-29b), 4.58 i 4.16 (qAB, 2H, *J* = 10.8 Hz, OCH_2), 4.48 (dd, 1H, J_1 = 5.4 Hz, J_2 = 11.4 Hz, H-3), 4.45 and 4.39 (qAB, 2H, *J* = 11.9 Hz, OCH_2), 4.30 (dd~t, 1H, J_1 = 9.0 Hz, J_2 = 9.5 Hz, H-2$_{gal}$), 4.29 (d, 1H, *J* = 10.0 Hz, H-28a), 4.04 (d, 1H, *J* = 2.7 Hz, H-4$_{gal}$), 3.86 (d, 1H, *J* = 10.8 Hz, H-28b), 3.80 (dd~t, 1H, J_1 = 5.8 Hz, J_2 = 6.8 Hz, H-5$_{gal}$), 3.74 (dd, 1H, J_1 = 2.7 Hz, J_2 = 9.5 Hz, H-3$_{gal}$), 3.59 (dd, 1H, J_1 = 6.8 Hz, J_2 = 9.2 Hz, H-6a$_{gal}$), 3.57 (dd, 1H, J_1=5.8 Hz, J_2=9.2 Hz, H-6b$_{gal}$), 2.63-2.58 (m, 4H, O(CO)CH_2CH_2), 2.41 (m, 1H, H-19), 2.03 (s, 3H, CH_3CO), 1.68 (s, 3H, CH_3-30), 1.97 - 0.77 (m, 24H, CH, CH_2, BN scaffold), 1.02, 0.98, 0.84, 0.83 (all s, 15H, CH_3-23 - CH_3-27) ppm. ^{13}C NMR (150 MHz, $CDCl_3$) δ_C: 172.49 (CO), 172.12 (CO), 171.08 (CO), 150.18 (C-20), 142.86 (C$_{triaz}$), 138.46 (C-Ph), 137.94 (C-Ph), 137.62 (C-Ph), 137.29 (C-Ph), 128.62 (CH-Ph), 128.56 (CH-Ph), 128.44 (CH-Ph), 128.38 (CH-Ph), 128.17 (CH-Ph), 128.05 (CH-Ph), 128.03 (CH-Ph), 127.97 (CH-Ph), 127.91(CH-Ph), 127.85(CH-Ph), 127.67(CH-Ph), 122.60 (CH$_{triaz}$), 109.98 (C-29), 88.04 (C-1$_{gal}$), 83.17, 81.02, 76.50, 75.18, 74.89, 73.69, 73.33, 72.87 (C-5$_{glu}$, C-2$_{glu}$, C-3$_{glu}$, C-4$_{glu}$, O$\underline{C}H_2$-Ph), 68.25 (C-6$_{gal}$), 63.16 (C-28), 57.92 (OCH_2), 55.48 (C-5), 50.37 (C-9), 48.89 (C-18), 47.80 (C-19), 46.51 (C-17), 42.79 (C-14), 40.99 (C-8), 38.49 (C-1), 37.90 (C-4), 37.68 (C-13), 37.16 (C-10), 34.61 (C-22), 34.21 (C-7), 29.81 (C-21), 29.67 (C-16), 29.24, 29.16 (O(CO)$\underline{C}H_2\underline{C}H_2$), 28.05 (C-23), 27.13 (C-15), 25.26 (C-12), 23.80 (C-2), 21.42 (C-11), 20.89 ($\underline{C}H_3$CO), 19.24 (C-30), 18.27 (C-6), 16.60 (C-25), 16.26 (C-26), 16.14 (C-24), 14.83 (C-27) ppm.

Product ***7a*** *(3-O-Ac-28-O'-SAOCH$_2$Tri(CH$_2$)$_2$OGlu(OAc)BN)* was obtained as a white solid, 56% yield. $[\alpha]^{27}_{D}$ = 0.9 (c=1, $CHCl_3$); HRMS (ESI$^+$): calcd for $C_{55}H_{81}N_3O_{16}$ ([M+Na]$^+$): *m/z* 1062.5515; found: *m/z* 1062.5526; ^{1}H NMR (600 MHz, $CDCl_3$) δ_H: 7.65 (s, br, 1H, H-5$_{triaz}$), 5.22 (qAB, 2H, *J* = 12.8 Hz, OCH_2), 5.17 (dd~t, 1H, J_1 = 9.5 Hz, J_2 = 9.6 Hz, H-3$_{glu}$), 5.06 (dd~t, 1H, J_1 = 9.5 Hz, J_2 = 10.0 Hz, H-4$_{glu}$), 4.98 (dd~t, 1H, J_1 = 8.0 Hz, J_2 = 9.6 Hz, H-2$_{glu}$), 4.67 (s, br, 1H, H-29a), 4.60 (m, 1H, CH), 4.58 (s, br, 1H, H-29b), 4.50 (m, 1H, CH), 4.47 (d, 1H, *J* = 8.0 Hz, H-1$_{glu}$), 4.46 (dd, 1H, J_1 = 10.2 Hz, J_2 = 4.8 Hz, H-3), 4.28 (d, 1H, *J* = 11.4 Hz, H-28a), 4.24 (dd, 1H, J_1 = 4.7 Hz, J_2 = 12.3 Hz, H-6a$_{glu}$), 4.21 (m, 1H, CH), 4.13 (dd, 1H, J_1 = 2.3 Hz, J_2 = 12.3 Hz, H-6b$_{glu}$), 3.93 (m, 1H, CH), 3.87 (d, 1H, *J* = 10.8 Hz, H-28b), 3.68 (ddd, 1H, J_1 = 2.3 Hz, J_2 = 4.7 Hz, J_3 = 10.0 Hz, H-5$_{glu}$), 2.67 (s, br, 4H, O(CO)CH_2CH_2), 2.44 (m, 1H, H-19), 2.08, 2.03, 2.01, 1.99, 1.95 (all s, 3H each 5 x CH_3CO), 1.68 (s, 3H, CH_3-30), 1.83 - 0.67 (m, 24H, CH, CH_2, BN scaffold), 1.02, 0.97, 0.84, 0.83 (all s, 15H, CH_3-23 - CH_3-27) ppm; ^{13}C NMR (150 MHz, $CDCl_3$) δ_C: 172.54 (CO), 172.14 (CO), 171.05 (CO), 170.60 (CO), 170.15 (CO), 169.43 (CO), 169.28 (CO), 150.17 (C-20), 142.69 (C$_{triaz}$), 124.99 (CH$_{triaz}$), 109.98 (C-29), 100.65 (C-1$_{glu}$), 81.02 (C-3), 72.58, 72.14, 71.05, 68.35 (C-5$_{glu}$, C-2$_{glu}$, C-3$_{glu}$, C-4$_{glu}$), 67.71 (O$\underline{C}H_2CH_2$), 63.17(C-28), 61.84 (C-6$_{glu}$), 57.95 (OCH_2), 55.49 (C-5), 50.39 (C-9), 50.17 ($OCH_2\underline{C}H_2$), 48.92 (C-18), 47.82 (C-19), 46.54 (C-17), 42.80 (C-14), 41.01 (C-8), 38.50 (C-1), 37.90 (C-4), 37.70 (C-13), 37.18 (C-10), 34.60 (C-22), 34.23 (C-7), 29.83 (C-21), 29.69 (C-16), 29.26, 29.16 (O(CO)$\underline{C}H_2\underline{C}H_2$), 28.05 (C-23), 27.15 (C-15), 25.28 (C-12), 23.80 (C-2), 21.38 (C-11), 20.90, 20.80, 20.63 ($\underline{C}H_3$CO), 19.23 (C-30), 18.28 (C-6), 16.58 (C-25), 16.25 (C-26), 16.14 (C-24), 14.83 (C-27) ppm.

Product ***7b*** (*3-O-Ac-28-O'-SAOCH$_2$Tri(CH$_2$)$_3$OGlu(OAc)BN)* was obtained as a white solid, 69% yield. $[\alpha]^{28}_{D}$ = 5.8 (c=1, $CHCl_3$); HRMS (ESI$^+$): calcd for $C_{56}H_{83}N_3O_{16}$ ([M+Na]$^+$): *m/z* 1076.5671.; found: *m/z* 1076.5715; ^{1}H NMR (600 MHz, $CDCl_3$) δ_H: 7.62 (s, br, 1H, H-5$_{triaz}$), 5.23 (qAB, 2H, *J* = 11.6 Hz, OCH_2), 5.22 (dd~t, 1H, J_1 = 11.6 Hz, J_2 = 5.6 Hz, H-3$_{glu}$), 5.09 (dd~t, 1H, J_1 = 9.7 Hz, J_2 = 8.0 Hz, H-4$_{glu}$), 5.01 (dd, 1H, J_1=9.7 Hz, J_2=8.0 Hz, H-2$_{glu}$), 4.68 (s, br, 1H, H-29a), 4.59 (s, br, 1H, H-29b), 4.51 (d, 1H, *J* = 8.0 Hz, H-1$_{glu}$), 4.46 (m, 1H, CH), 4.39 (m, 1H, CH), 4.29 (d, 1H, *J* = 3.8 Hz, H-28a), 4.27 (dd, 1H, J_1 = 8.1 Hz, J_2 = 4.3 Hz, H-6a$_{glu}$), 4.15 (dd, 1H, J_1 = 2.4 Hz, J_2 = 12.4 Hz, H-6b$_{glu}$), 3.88 (d, 1H, *J* = 10.5 Hz, H-28b), 3.87 (m, 1H, CH), 3.70 (ddd, 1H, J_1 = 2.4 Hz, J_2 = 4.7 Hz, J_3 = 10.0 Hz, H-5$_{glu}$), 3.50 (m, 1H, CH), 2.66 (s, br, 4H, O(CO)CH_2CH_2), 2.43 (m, 1H, H-19), 2.35 (m, 1H, CH), 2.18 (m, 1H, CH), 2.083, 2.080, 2.037, 2.029, 2.013 (all s, 3H each 5 x CH_3CO), 1.68 (s, 3H, CH_3-30), 2.06 - 0.73 (m, 24H, CH, CH_2, BN scaffold), 1.02, 0.97, 0.84, 0.835, 0.79 (all s, 15H, CH_3-23 - CH_3-27) ppm. ^{13}C NMR (150 MHz, $CDCl_3$) δ_C: 172.50 (CO), 172.15 (CO), 171.01 (CO), 170.58 (CO), 170.20 (CO), 169.44 (CO), 150.11 (C-20), 142.73 (C$_{triaz}$), 124.13 (CH$_{triaz}$), 109.97 (C-29), 100.84 (C-1$_{glu}$), 80.98 (C-3), 72.80, 72.02, 71.36, 68.43 (C-5$_{glu}$, C-2$_{glu}$, C-3$_{glu}$, C-4$_{glu}$), 65.88 (O$\underline{C}H_2CH_2CH_2$), 63.14 (C-28), 61.88 (C-6$_{glu}$), 57.98 (OCH_2), 55.46 (C-5), 50.36 (C-9), 48.88 (C-18), 47.80 (C-19), 46.79 ($OCH_2CH_2\underline{C}H_2$), 46.51 (C-17), 42.77 (C-14,) 40.98 (C-8), 38.47 (C-1), 37.87 (C-4), 37.68 (C-13), 37.15 (C-10), 34.57 (C-22), 34.20 (C-7), 30.25 (C-21), 29.80 (C-16), 29.65 ($OCH_2\underline{C}H_2CH_2$), 29.24, 29.18

$(O(CO)\underline{C}H_2\underline{C}H_2)$, 28.02 (C-23), 27.12 (C-15), 25.25 (C-12), 23.77 (C-2), 21.50 (C-11), 21.35, 20.87, 20.77, 20.63 $(\underline{C}H_3CO)$, 19.19 (C-30), 18.25 (C-6), 16.56 (C-25), 16.22 (C-26), 16.11 (C-24), 14.81 (C-27) ppm.

Product ***7c*** *(28-O'-SAOCH$_2$Tri(CH$_2$)$_2$OGlu(OAc)BN)* was obtained as a white solid, 63% yield. $[\alpha]^{28}_D$ = -4.0 (c=1, $CHCl_3$); HRMS (ESI$^+$): calcd for $C_{53}H_{79}N_3O_{15}$ ($[M+Na]^+$): *m/z* 1020.5409; found: *m/z* 1020.5486; ^{1}H NMR (600 MHz, $CDCl_3$) δ_H: 7.66 (s, br, 1H, H-5$_{triaz}$), 5.23 (qAB, 2H, *J* = 12.8 Hz, OCH_2), 5.17 (dd~t, 1H, J_1 = 9.5 Hz, J_2 = 9.6 Hz, H-3$_{glu}$), 5.07 (dd~t, 1H, J_1 = 9.5 Hz, J_2 = 10.0 Hz, H-4$_{glu}$), 4.98 (dd~t, 1H, J_1 = 8.0 Hz, J_2 = 9.6 Hz, H-2$_{glu}$), 4.68 (s, br, 1H, H-29a), 4.60 (m, 1H, CH), 4.59 (s, br, 1H, H-29b), 4.53 (m, 1H, CH), 4.48 (d, 1H, *J* = 8.0 Hz, H-1$_{glu}$), 4.28 (d, 1H, *J* = 11.4 Hz, H-28a), 4.25 (dd, 1H, J_1 = 4.7 Hz, J_2 = 12.4 Hz, H-6a$_{glu}$), 4.23 (m, 1H, CH), 4.04 (dd, 1H, J_1 = 2.3 Hz, J_2 = 12.4 Hz, H-6b$_{glu}$), 3.95 (m, 1H, CH), 3.88 (d, 1H, *J* = 11.4 Hz, H-28b), 3.70 (ddd, 1H, J_1 = 2.3 Hz, J_2 = 4.7 Hz, J_3 = 10.0 Hz, H-5$_{glu}$), 3.18 (dd, 1H, J_1 = 4.8 Hz, J_2 = 11.4 Hz, H-3), 2.67 (s, br, 4H, $O(CO)CH_2CH_2$), 2.44 (m, 1H, H-19), 2.09, 2.02, 2.00, 1.96 (all s, 3H each, 4 x CH_3CO), 1.68 (s, 3H, CH_3-30), 1.83 - 0.67 (m, 24H, CH, CH_2, BN scaffold), 1.02, 0.97, 0.966, 0.82, 0.76 (all s, 15H, CH_3-23 - CH_3-27) ppm. ^{13}C NMR (150 MHz, $CDCl_3$) δ_C: 172.58 (CO), 172.18 (CO), 170.66 (CO), 170.20 (CO), 169.48 (CO), 169.33 (CO), 150.22 (C-20), 142.70 (C$_{triaz}$), 125.04 (CH$_{triaz}$), 109.97 (C-29), 100.67 (C-1$_{glu}$), 79.07 (C-3), 72.59, 72.15, 71.05, 68.35 (C-5$_{glu}$, C-2$_{glu}$, C-3$_{glu}$, C-4$_{glu}$), 67.73 $(O\underline{C}H_2CH_2)$, 63.19 (C-28), 61.85 (C-6$_{glu}$), 57.96 (OCH_2), 55.43 (C-5), 50.50 (C-9), 50.20 $(OCH_2\underline{C}H_2)$, 48.94 (C-18), 47.82 (C-19), 46.56 (C-17), 42.83 (C-14), 41.01 (C-8), 38.99 (C-1), 38.84 (C-4) 37.73 (C-13), 37.28 (C-10), 34.60 (C-22), 34.23 (C-7), 29.83 (C-21), 29.71 (C-16), 29.27, 29.18 $(O(CO)\underline{C}H_2\underline{C}H_2)$, 28.05 (C-23), 27.17 (C-15), 25.34 (C-12), 23.80 (C-2), 21.89 (C-11), 20.91, 20.84, 20.67 $(\underline{C}H_3CO)$, 19.27 (C-30), 18.41 (C-6), 16.58 (C-25), 16.25 (C-26), 16.16 (C-24), 14.89 (C-27) ppm.

Product ***7d*** *(28-O'-SAOCH$_2$Tri(CH$_2$)$_3$OGlu(OAc)BN)* was obtained as a white solid, 70% yield. $[\alpha]^{28}_D$ = -1.0 (c=1, $CHCl_3$); HRMS (ESI$^+$): calcd for $C_{54}H_{81}N_3O_{15}$ ($[M+Na]^+$): *m/z* 1034.5565; found: *m/z* 1034.5626; ^{1}H NMR (600 MHz, $CDCl_3$) δ_H: 7.62 (s, br, 1H, H-5$_{triaz}$), 5.23 (qAB, 2H, *J* = 11.7 Hz, OCH_2), 5.22 (dd~t, 1H, J_1 = 9.4 Hz, J_2 = 9.6 Hz, H-3$_{glu}$), 5.09 (dd~t, 1H, J_1 = 9.4 Hz, J_2 = 10.0 Hz, H-4$_{glu}$), 5.01 (dd, 1H, J_1 = 8.0 Hz, J_2 = 9.7 Hz, H-2$_{glu}$), 4.68 (s, br, 1H, H-29a), 4.58 (s, br, 1H, H-29b), 4.51 (d, 1H, *J* = 8.0 Hz, H-1$_{glu}$), 4.47 (m, 1H, CH), 4.39 (m, 1H, CH), 4.28 (d, 1H, *J* = 11.4 Hz, H-28a), 4.27 (dd, 1H, J_1 = 4.7 Hz, J_2 = 12.4 Hz, H-6a$_{glu}$), 4.15 (dd, 1H, J_1 = 2.4 Hz, J_2 = 12.4 Hz, H-6b$_{glu}$), 3.88 (d, 1H, *J* = 10.8 Hz, H-28b), 3.87 (m, 1H, CH), 3.70 (ddd, 1H, J_1 = 2.4 Hz, J_2 = 4.7 Hz, J_3 = 10.0 Hz, H-5$_{glu}$), 3.49 (m, 1H, CH), 3.18 (dd, 1H, J_1 = 11.4 Hz, J_2 = 4.2 Hz, H-3), 2.66 (s, br, 4H, $O(CO)CH_2CH_2$), 2.43 (m, 1H, H-19), 2.22 (m, 1H, CH), 2.15 (m, 1H, CH), 2.084, 2.081, 2.03, 2.01 (all s, 3H each, 4 x CH_3CO), 1.68 (s, 3H, CH_3-30), 2.26 - 0.67 (m, 24H, CH, CH_2, BN scaffold), 1.02, 0.97, 0.965, 0.82, 0.76 (all s, 15H, CH_3-23 - CH_3-27) ppm. ^{13}C NMR (150 MHz, $CDCl_3$) δ_C: 172.56 (CO), 172.22 (CO), 170.67 (CO), 170.28 (CO), 169.51 (CO), 150.19 (C-20), 142.78 (C$_{triaz}$), 124.18 (CH$_{triaz}$), 109.98 (C-29), 100.89 (C-1$_{glu}$), 79.05 (C-3), 72.84, 72.06, 71.39, 68.46 (C-5$_{glu}$, C-2$_{glu}$, C-3$_{glu}$, C-4$_{glu}$), 65.93 $(O\underline{C}H_2CH_2CH_2)$, 63.20 (C-28), 61.92 (C-6$_{glu}$), 58.02 (OCH_2), 55.42 (C-5), 50.49 (C-9), 48.93 (C-18), 47.82 (C-19), 46.86 $(OCH_2CH_2\underline{C}H_2)$, 46.56 (C-17), 42.82 (C-14), 41.00 (C-8), 38.98 (C-1), 38.84 (C-4), 37.73 (C-13), 37.27 (C-10), 34.62 (C-22), 34.31 (C-7), 30.29 (C-21), 29.86 (C-16), 29.51 $(OCH_2\underline{C}H_2CH_2)$, 29.28, 29.22 $(O(CO)\underline{C}H_2\underline{C}H_2)$, 28.11 (C-23), 27.52 (C-15), 27.17 (C-12), 25.33 (C-2), 21.88 (C-11), 20.90, 20.85, 20.83, 20.70 $(\underline{C}H_3CO)$, 19.25 (C-30), 18.41 (C-6), 16.21 (C-25), 16.15 (C-26), 15.49 (C-24), 14.89 (C-27) ppm.

Product ***8a*** *(28-O'-SAOCH$_2$TriGal(OAc)BN C-6)* was obtained as a white solid with a 12% yield. $[\alpha]^{28}_D$ = 11.3 (c= 0.5, $CHCl_3$); ^{1}H NMR (600 MHz, $CDCl_3$) δ_H: 7.64 (s, br, 1H, H-5$_{triaz}$), 6,34 (d, 1H, *J* = 4.7 Hz, H-1$_{gal}$), 5.60 (dd, 1H, J_1 = 6.3 Hz, J_2 = 7.6 Hz, H-4$_{gal}$), 5.50 (ddd, 1H, J_1 = 3.5 Hz, J_2 = 5.5 Hz, J_3 = 7.1 Hz, H-5$_{gal}$), 5.42 (dd, 1H, J_1 = 11.3 Hz, J_2 = 5.5 Hz, H-3$_{gal}$), 5.30 (dd~t, 1H, J_1 = 4.6 Hz, J_2 = 7.6 Hz, H-2$_{gal}$), 5.17-5.27 (m, 2H, OCH_2), 4.68 (s, br, 1H, H-29a), 4.59 (s, br, 1H, H-29b), 4.47 (dd, 1H, J_1 = 10.8 Hz, J_2 = 5.5 Hz, H, 3.87 (d, 1H, *J* = 11.0 Hz, H-28b), 2.68-2.64 (m, 4H, $O(CO)CH_2CH_2$), 2.46-2.39 (m, 1H, H-19), 2.14, 2.13, 2.10, 2.09, 2.04 (all s, 3H each, 5 x CH_3CO), 1.68 (s, 3H, CH_3-30), 1.88 - 0.73 (m, 24H, CH, CH_2, BN scaffold), 1.02, 0.97, 0.86, 0.84, 0.83 (all s, 15H, CH_3-23 - CH_3-27) ppm. ^{13}C NMR (150 MHz, $CDCl_3$) δ_C: 172.58 (CO), 172.28 (CO), 171.14 (CO), 170.58 (CO), 169.87 (CO), 169.45 (CO), 169.29 (CO), 150.22 (C-20), 143.15 (C$_{triaz}$), 110.05 (C-29), 99.18 (CH$_{triaz}$), 92.97 (C-1$_{gal}$), 81.07 (C-3), 75.38, 73.09, 70.62, 69.79 (C-5$_{gal}$, C-2$_{gal}$, C-3$_{gal}$, C-4$_{gal}$), 63.27 (C-28), 57.95 (C-6$_{gal}$), 55.54 (C-5), 50.43 (C-9), 49.68 (OCH_2) 48.95 (C-18), 47.87 (C-19), 46.58 (C-17), 42.85 (C-14), 41.05 (C-8), 38.55 (C-1), 37.95 (C-4), 37.75 (C-13), 37.22 (C-10), 34.65 (C-22), 34.28 (C-7), 29.87 (C-21), 29.72 (C-16), 29.30, 29.25 $(O(CO)\underline{C}H_2\underline{C}H_2)$, 28.10 (C-23), 27.19 (C-15), 25.32 (C-12), 23.85 (C-2), 21.12 (C-11), 21.00, 20.95, 20.76, 20.58 $(\underline{C}H_3CO)$, 19.27 (C-30), 18.32 (C-6), 16.64 (C-25), 16.31 (C-26), 16.19 (C-24), 14.88 (C-27) ppm.

2.4. Procedure for the Synthesis of Betulin Metabolite (9a)

In a 25 mL round-bottom flask, 3-*O*-acetyl-28-*O′*-(propynylcarbonyloxypropanoyl) betulin **3a** (80 mg, 0.129 mmol) was combined with 2-azidoethanol (11.2 mg, 0.129 mmol), isopropyl alcohol (1 mL), and tetrahydrofuran (1 mL). The mixture was stirred at room temperature under an argon atmosphere, and then solutions of $CuSO_4·5H_2O$ (6.5 mg; 0.026 mmol) in 0.5 mL of water and sodium ascorbate (11 mg; 0.051 mmol) in 0.5 mL of water were added. The reaction was carried out for 10 days, monitored by thin-layer chromatography (TLC, $CHCl_3$/MeOH 20:1, v/v). Then the mixture was extracted with DCM (2×10 mL), organic layers were combined and dried over anhydrous $MgSO_4$, the drying agent was filtered off, and the solvent was evaporated under diminished pressure. The crude residue was purified using column chromatography ($CHCl_3$/MeOH, gradient: 100:1 to 80:1, v/v).

Product ***9a*** *(3-O-Ac-28-O′-SAOCH$_2$Tri(CH$_2$)$_2$OHBN)* was obtained as a white solid, 32% yield. $[\alpha]^{25}_D$ = 15.4 (c= 1, $CHCl_3$), m.p. = 96–98°C; HRMS (ESI$^+$): calcd for $C_{41}H_{63}N_3O_7$ ([M+Na]$^+$): *m/z* 732.4564; found: *m/z* 732.4573; ^{1}H NMR (400 MHz, $CDCl_3$) δ_H: 7.71 (s, br, 1H, H-5$_{triaz}$), 5.20-5.27 (m, 2H, OCH_2), 4.68 (s, br, 1H, H-29a), 4.59 (s, br, 1H, H-29b), 4.28 (d, 1H, *J* = 9.6 Hz, H-28b), 4.50-4.40 (m, 2H, CH_2), 3.90-3.75 (m, 2H, CH_2), 2.67-2.63 (m, 4H, $O(CO)CH_2CH_2$), 2.42 (m, 1H, H-19), 2.04 (s, 3H, CH_3CO), 1.68 (s, 3H, CH_3-30), 1.92 - 0.73 (m, 24H, CH, CH_2, BN scaffold), 1.02, 0.97, 0.84, 0.83 (all s, 15H, CH_3-23 - CH_3-27) ppm. ^{13}C NMR (100 MHz, $CDCl_3$) δ_C: 172.45 (CO), 172.10 (CO), 170.96 (CO), 150.22 (C-20), 142.72 (C$_{triaz}$), 109.87 (C-29), 101.24 (CH$_{triaz}$), 80.87 (C-3), 69.34 (CH_2OH), 63.05 (C-28), 57.87 (OCH_2), 55.33 (C-5), 50.22 (C-9), 49.96 ($\underline{C}H_2CH_2OH$), 48.74 (C-18), 47.68 (C-19), 46.39 (C-17), 42.65 (C-14), 40.85 (C-8), 38.34 (C-1), 37.75 (C-4), 37.55 (C-13), 37.02 (C-10), 34.45 (C-22), 34.07 (C-7), 29.52 (C-16), 29.08, 29.04 ($O(CO)\underline{C}H_2\underline{C}H_2$), 27.90 (C-23), 26.99 (C-15), 25.11 (C-12), 23.65 (C-2), 21.27 (C-11), 20.75 ($\underline{C}H_3CO$), 19.08 (C-30), 18.13 (C-6), 16.45 (C-25), 16.12 (C-26), 16.00 (C-24), 14.89 (C-27) ppm.

3. NMR Spectra of Betulin Glycoconjugates

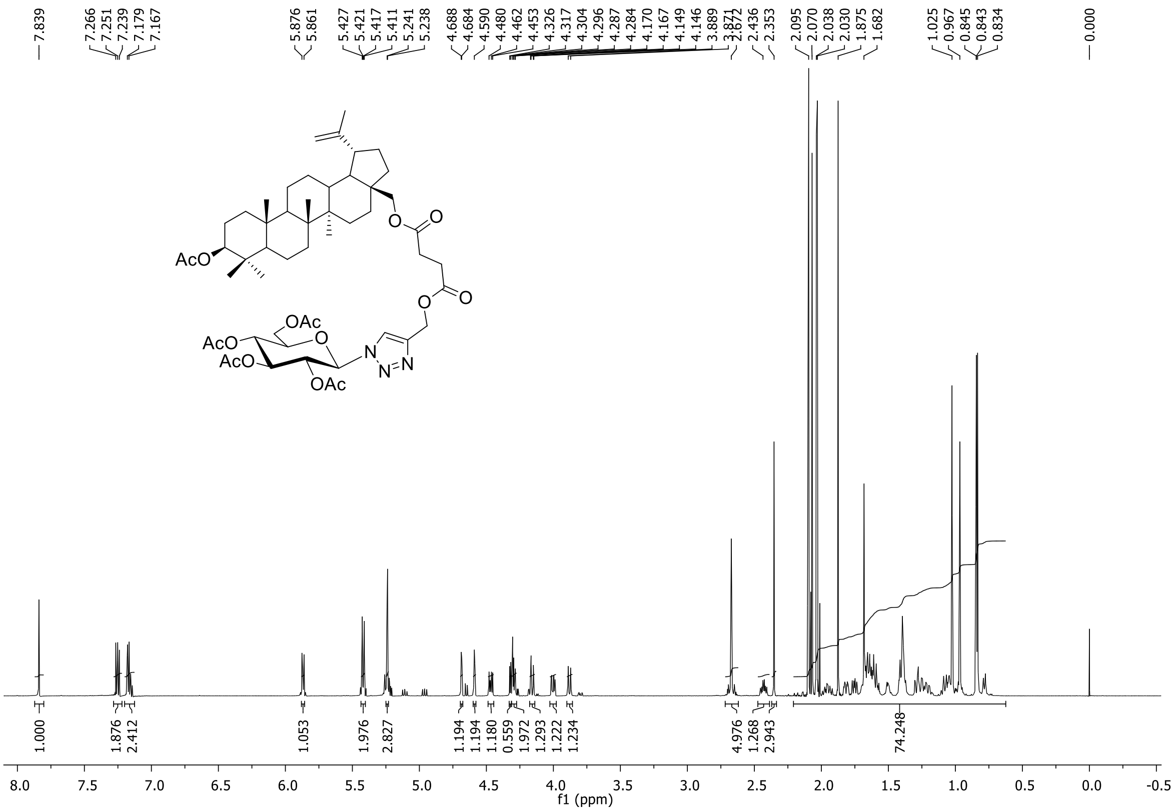

^{1}H-NMR spectrum of (**5a**); 600 MHz/$CDCl_3$/TMS; δ (ppm).

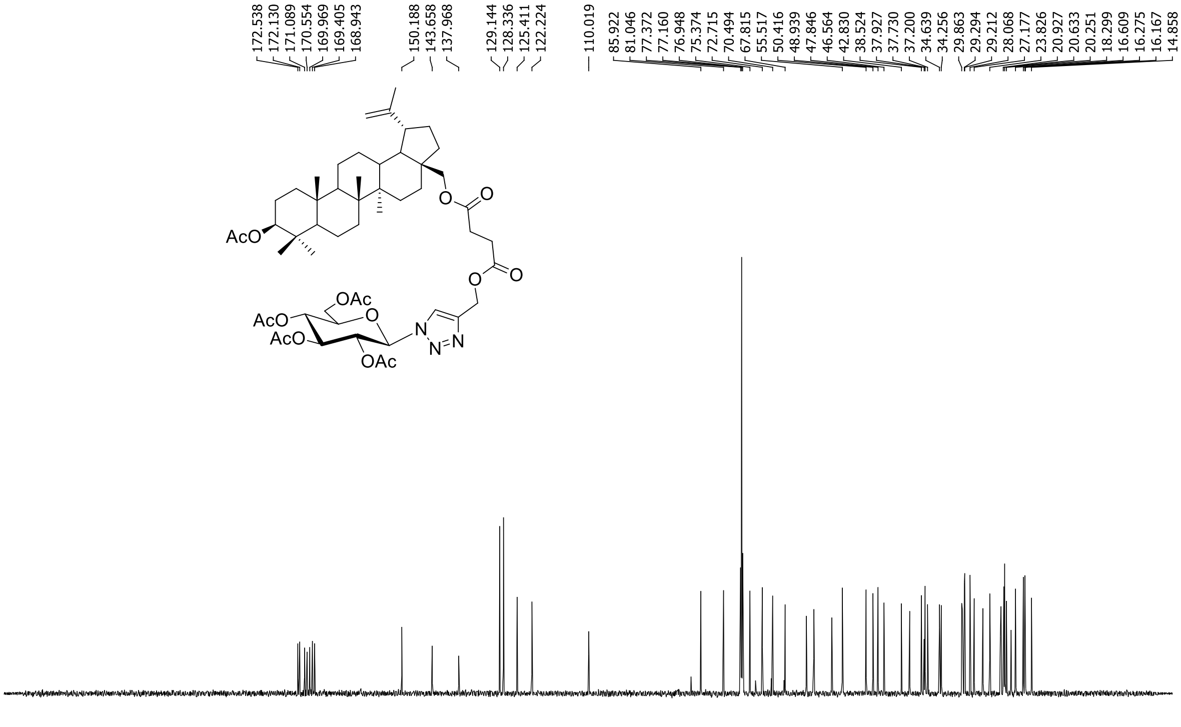

^{13}C-NMR spectrum of (**5a**); 150 MHz/$CDCl_3$/TMS; δ (ppm).

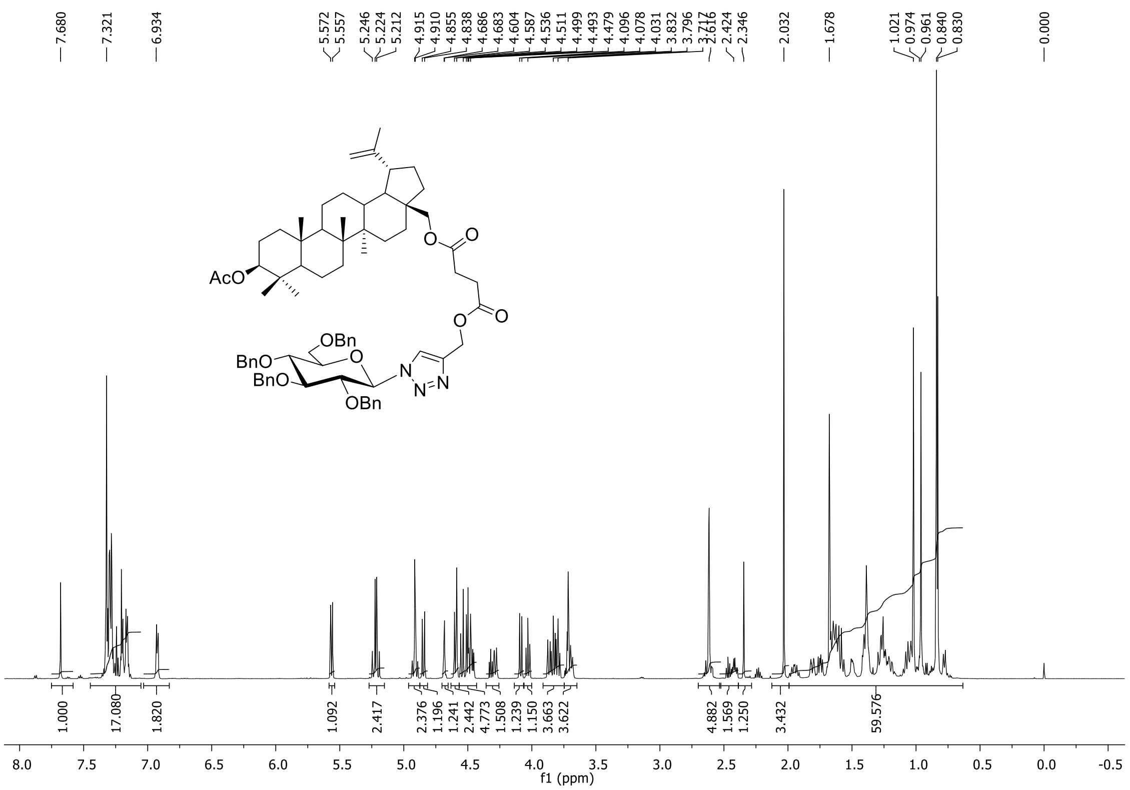

^{1}H-NMR spectrum of (**5b**); 600 MHz/CDCl3/TMS; δ (ppm).

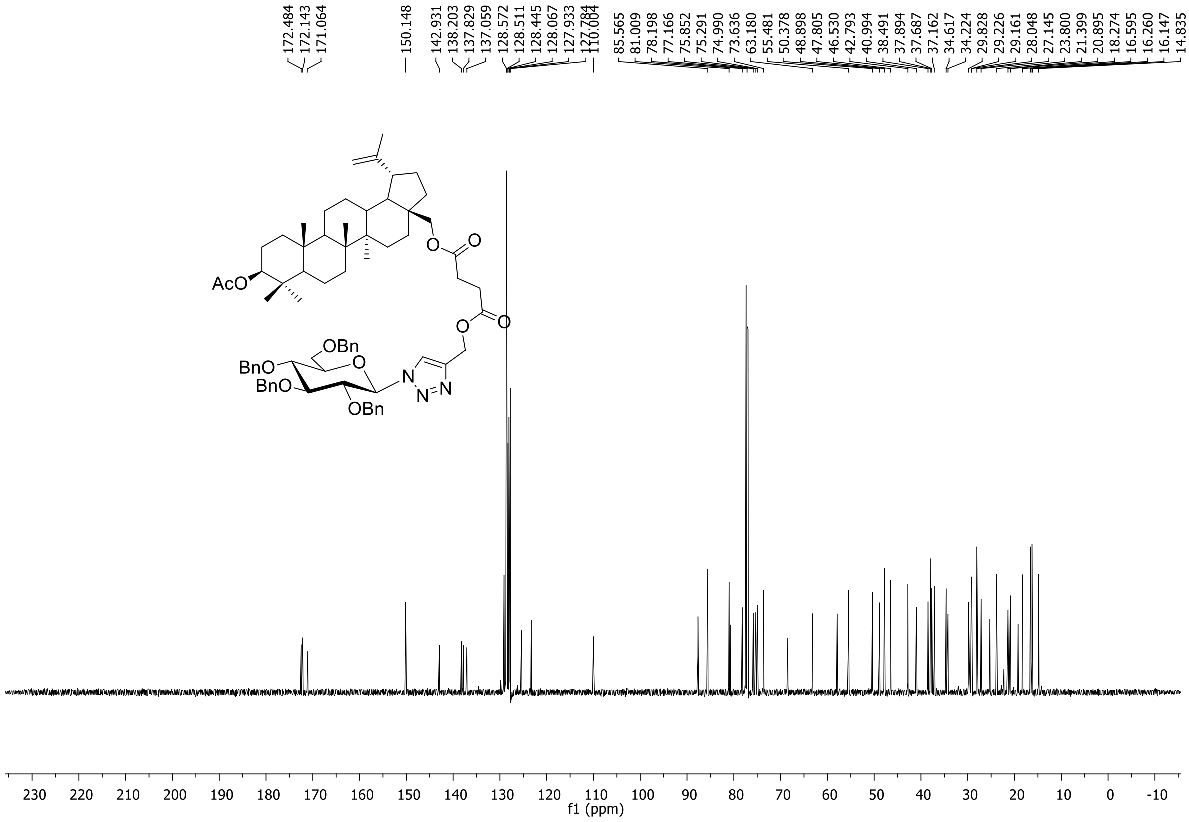

^{13}C-NMR spectrum of (**5b**); 150 MHz/CDCl3/TMS; δ (ppm).

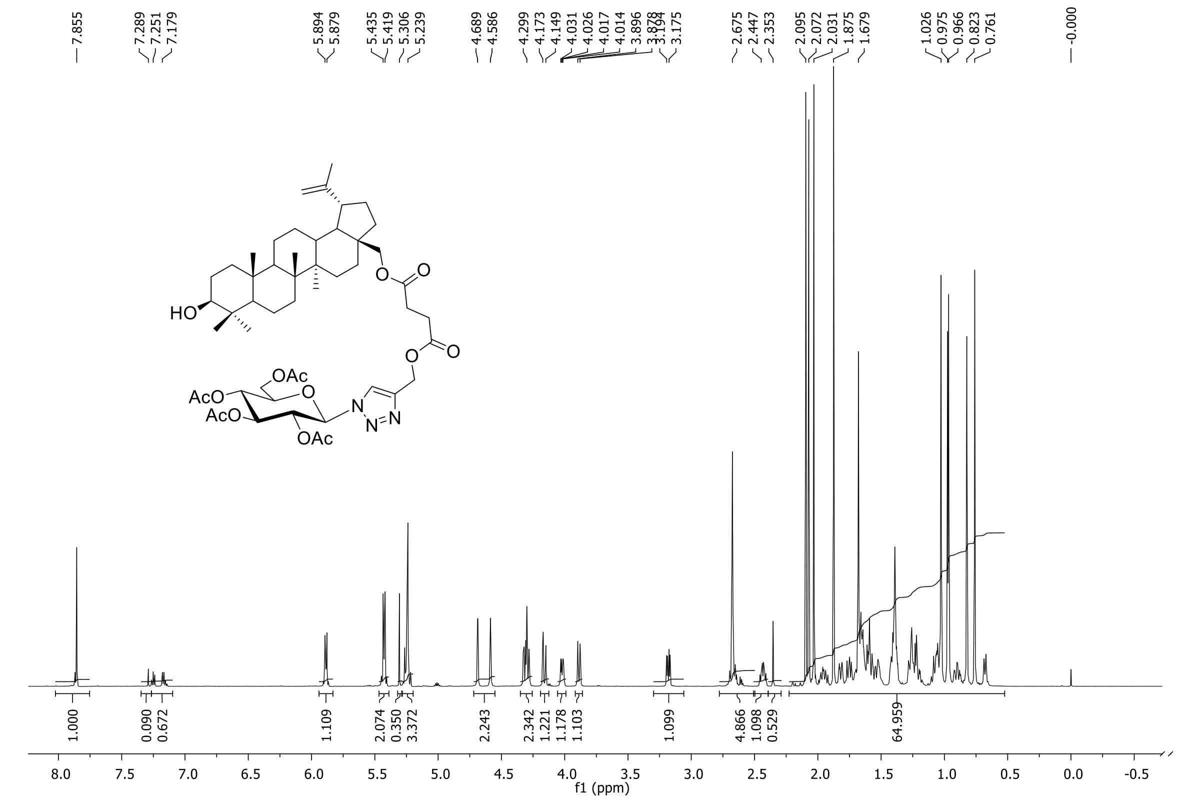

^{1}H-NMR spectrum of (**5c**); 600 MHz/CDCl$_3$/TMS; δ (ppm).

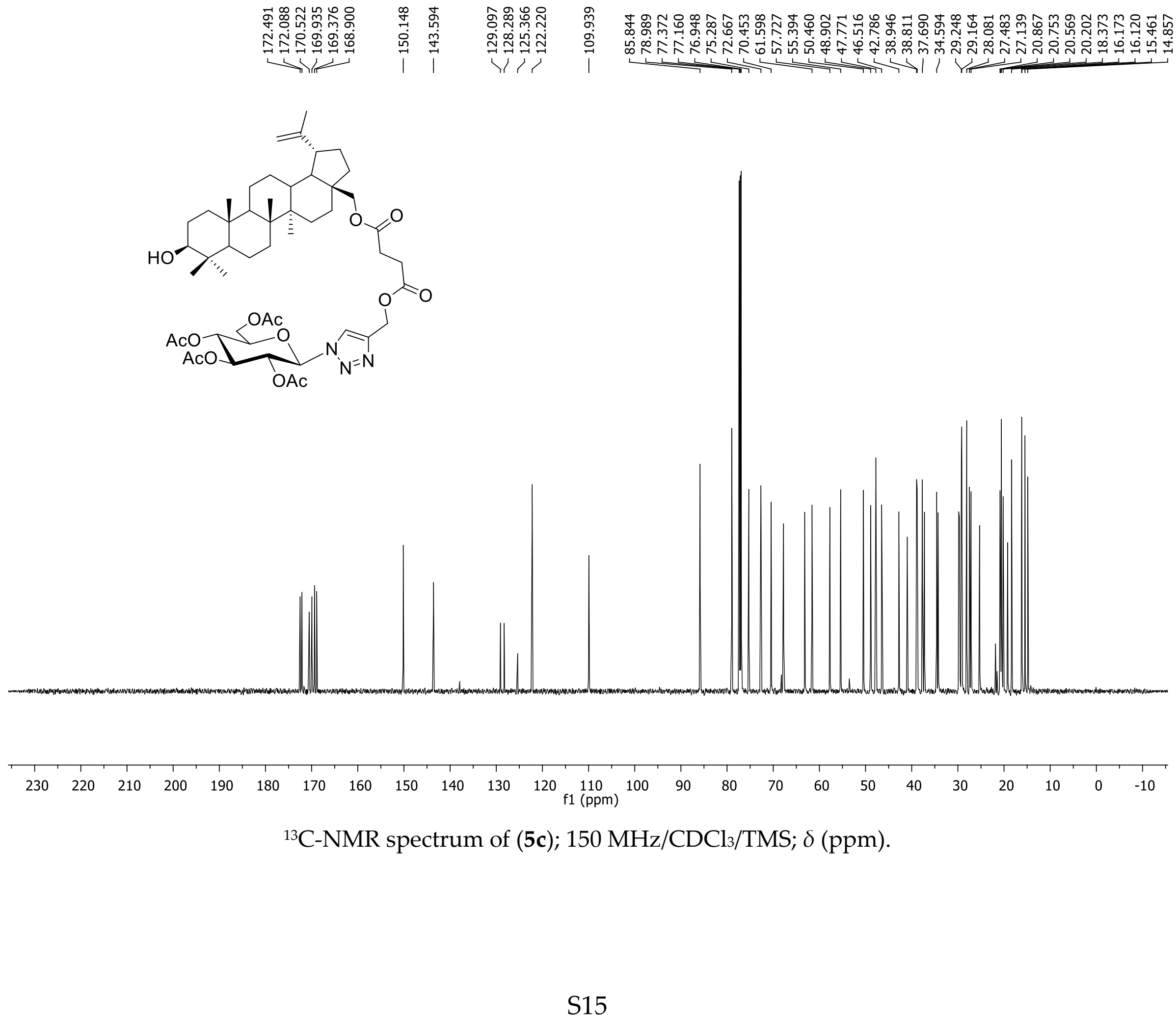

^{13}C-NMR spectrum of (**5c**); 150 MHz/CDCl$_3$/TMS; δ (ppm).

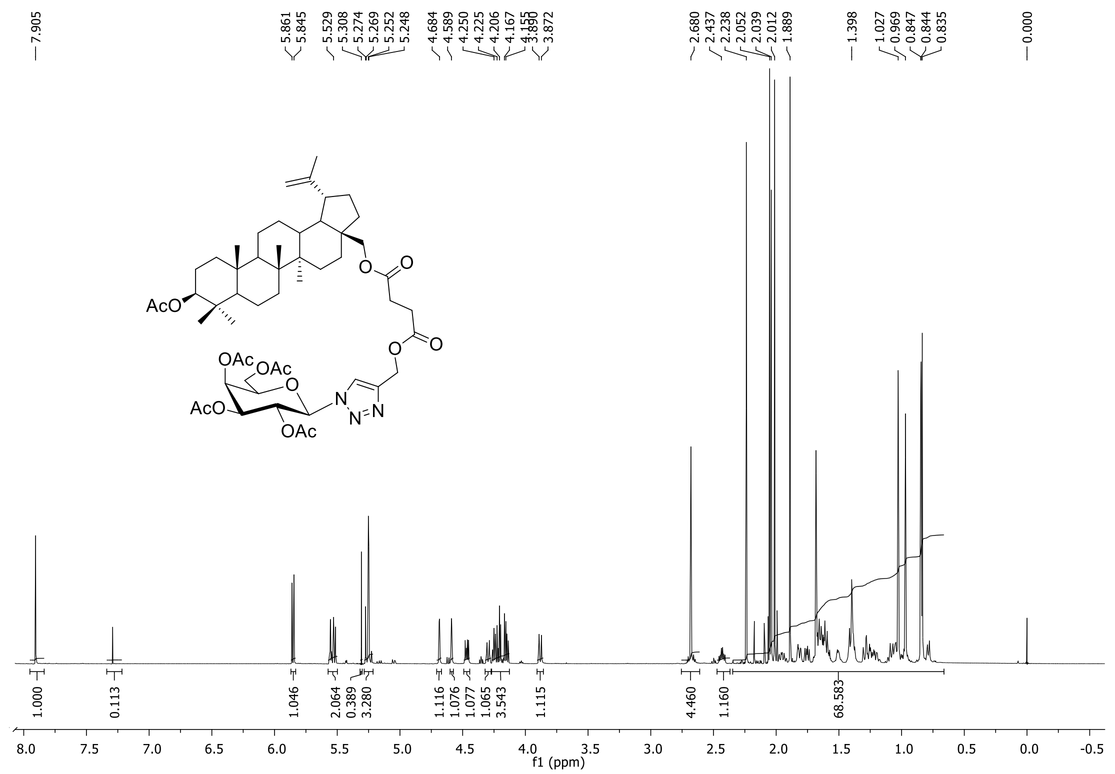

^{1}H-NMR spectrum of (**6a**); 600 MHz/$CDCl_3$/TMS; δ (ppm).

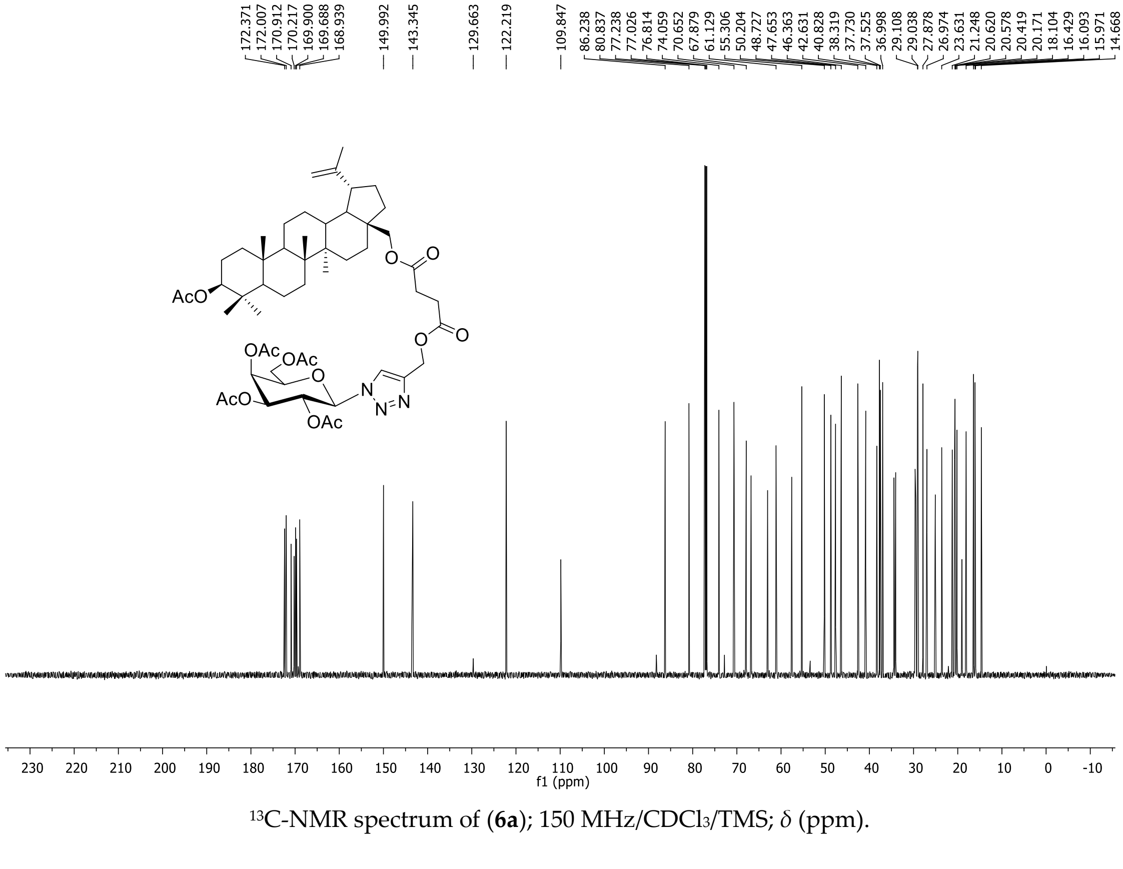

^{13}C-NMR spectrum of (**6a**); 150 MHz/$CDCl_3$/TMS; δ (ppm).

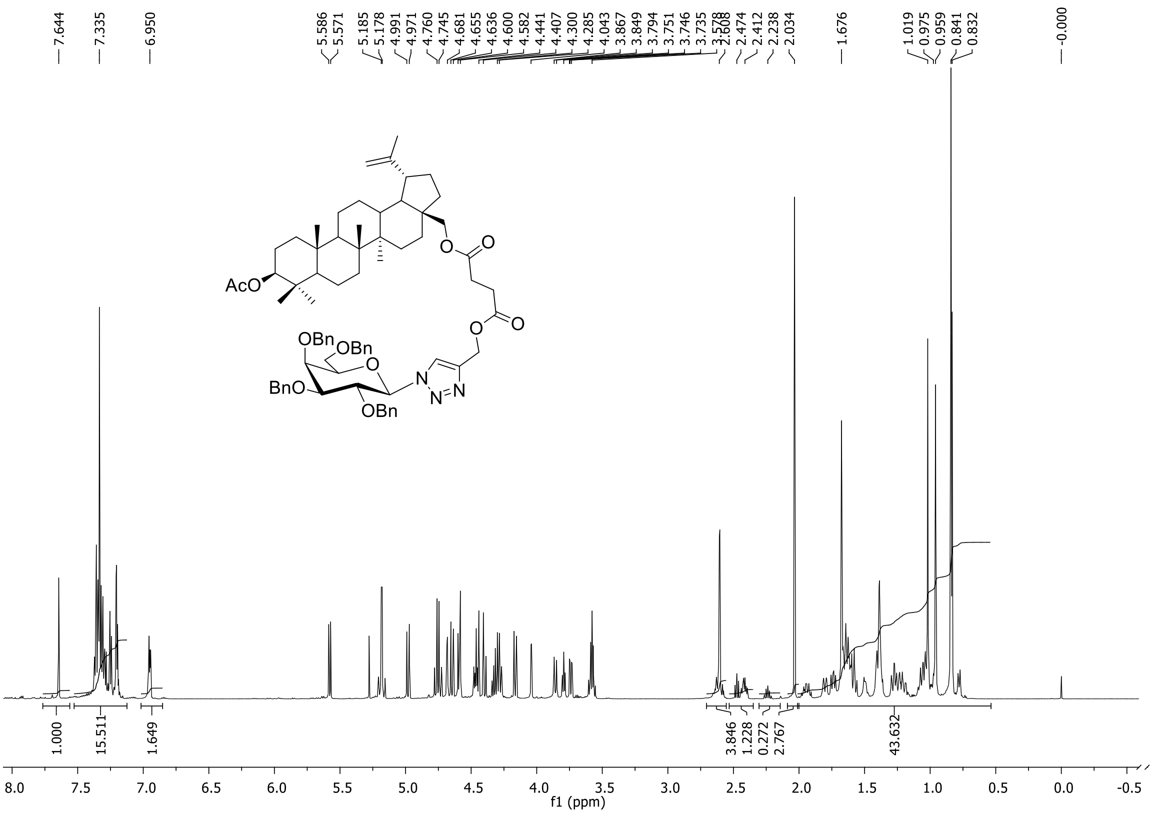

^{1}H-NMR spectrum of (**6b**); 600 MHz/CDCl3/TMS; *δ* (ppm).

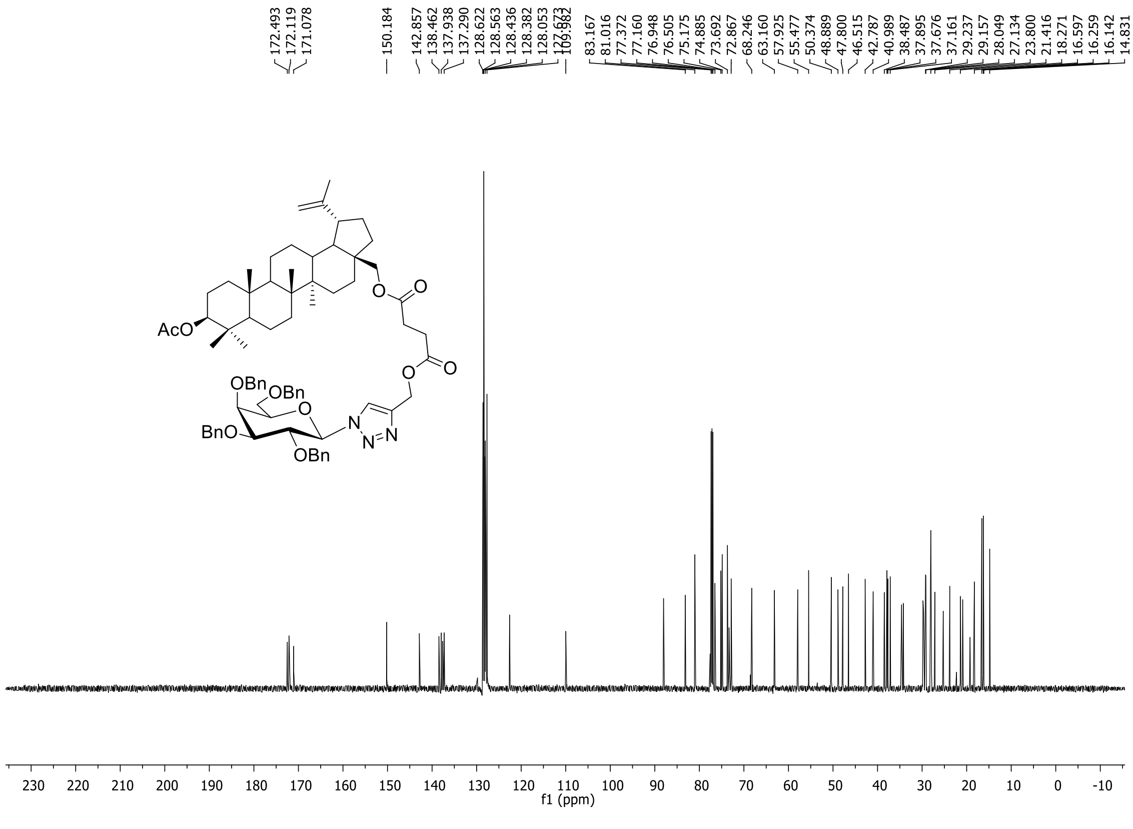

^{13}C-NMR spectrum of (**6b**); 150 MHz/CDCl3/TMS; *δ* (ppm).

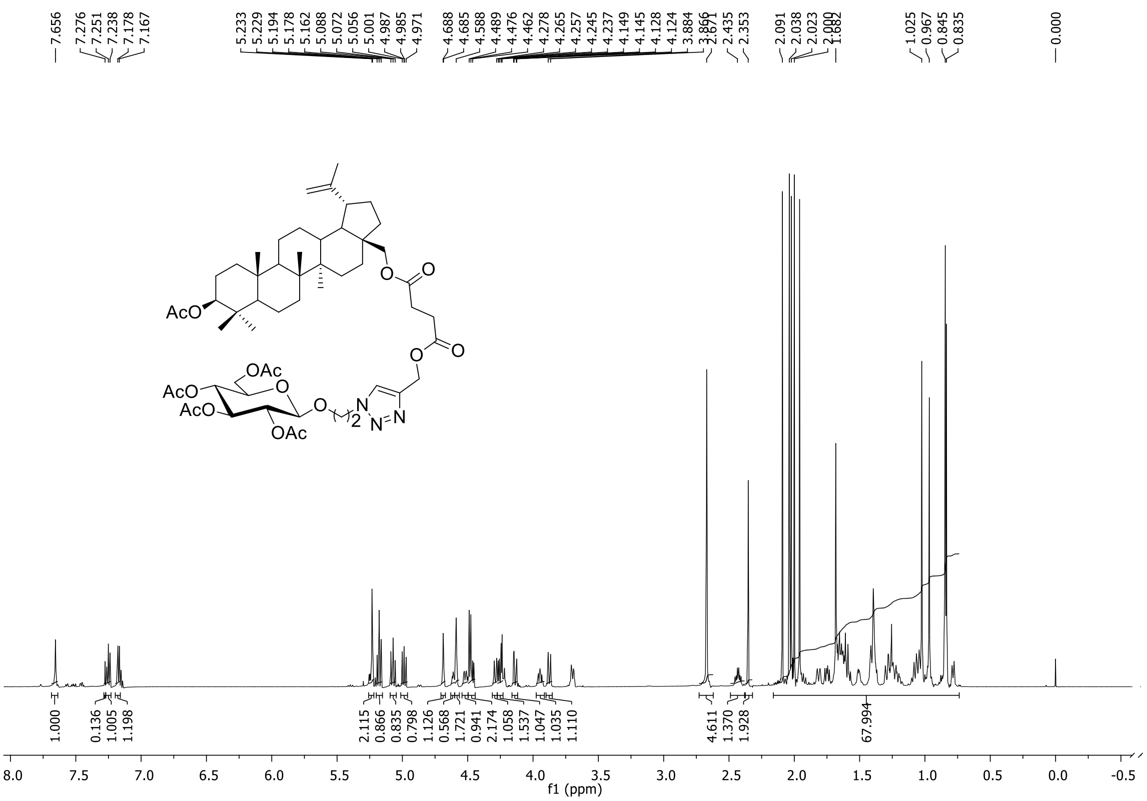

^{1}H-NMR spectrum of (**7a**); 600 MHz/$CDCl_3$/TMS; *δ* (ppm).

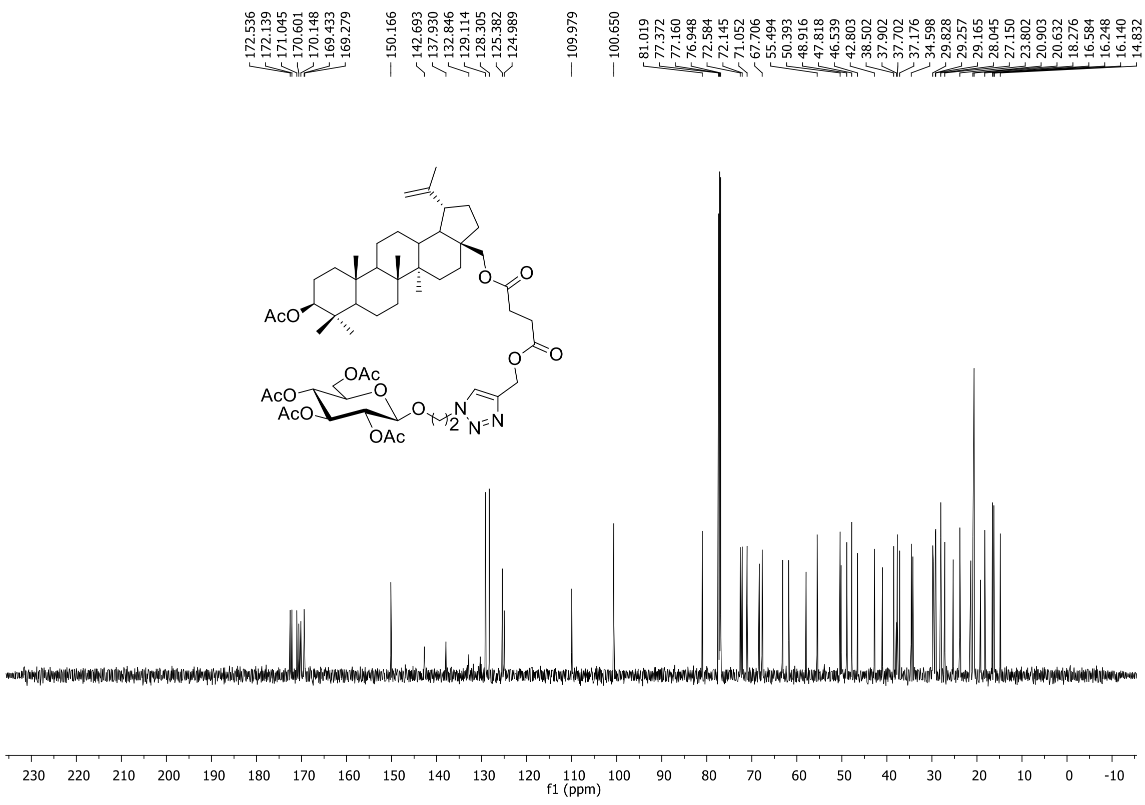

^{13}C-NMR spectrum of (**7a**); 150 MHz/$CDCl_3$/TMS; *δ* (ppm).

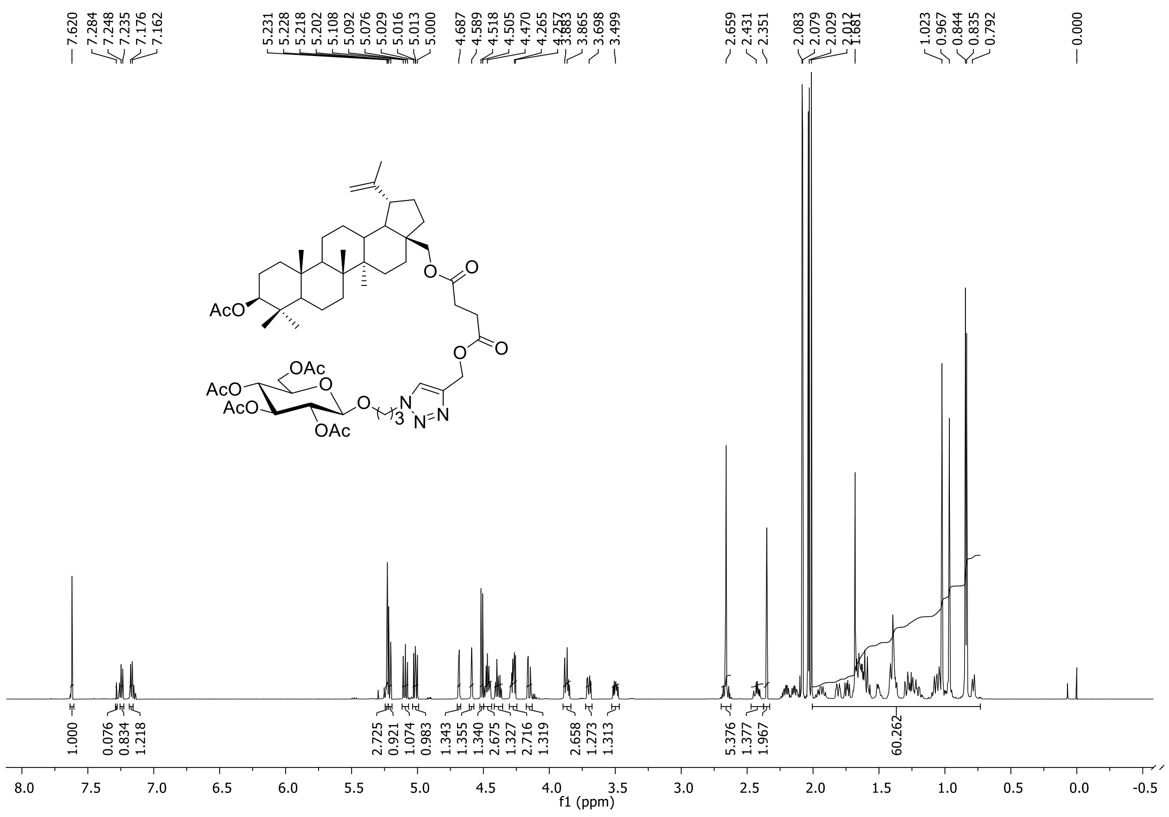

^{1}H-NMR spectrum of (**7b**); 600 MHz/$CDCl_3$/TMS; δ (ppm).

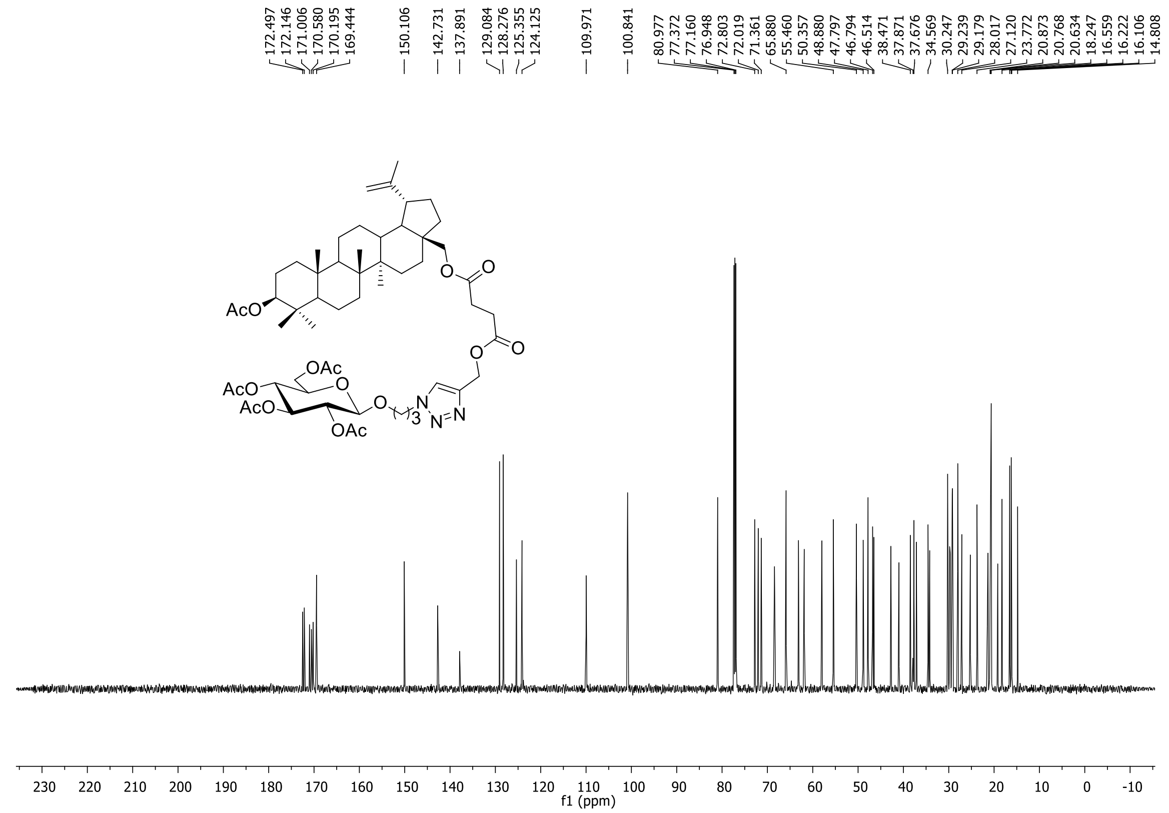

^{13}C-NMR spectrum of (**7b**); 150 MHz/$CDCl_3$/TMS; δ (ppm).

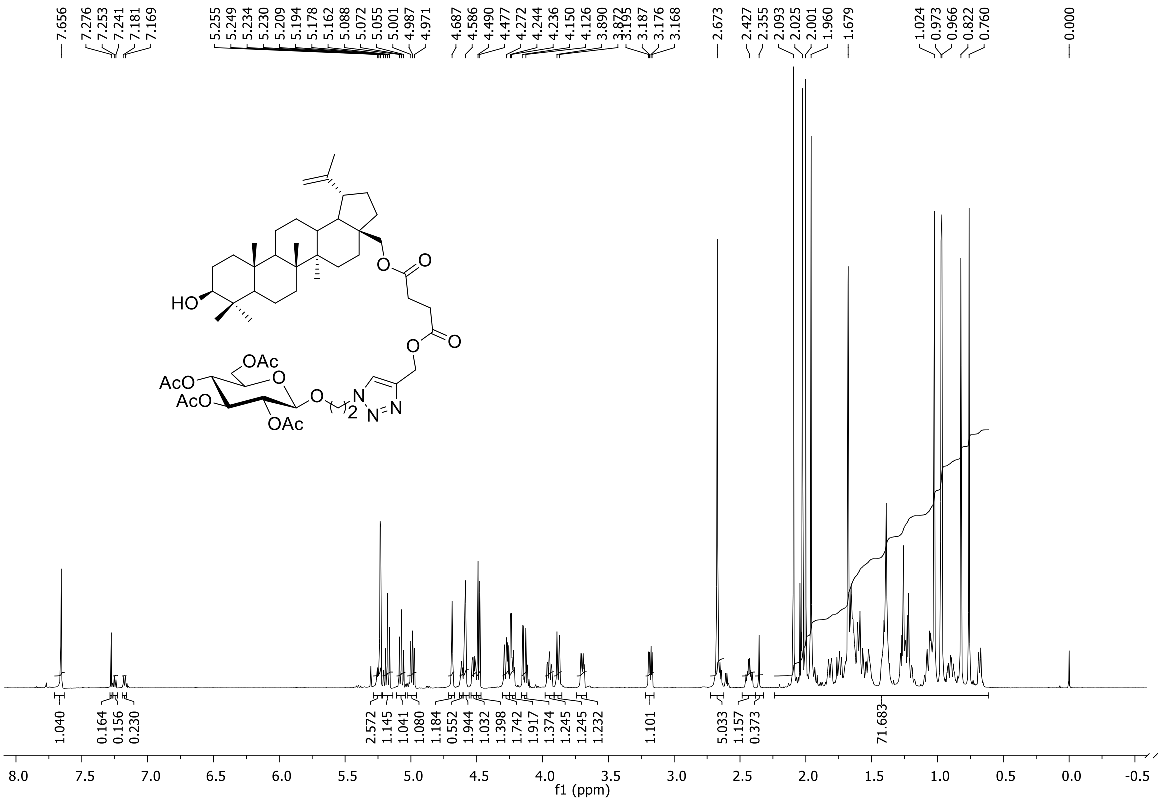

^{1}H-NMR spectrum of (**7c**); 600 MHz/$CDCl_3$/TMS; δ (ppm).

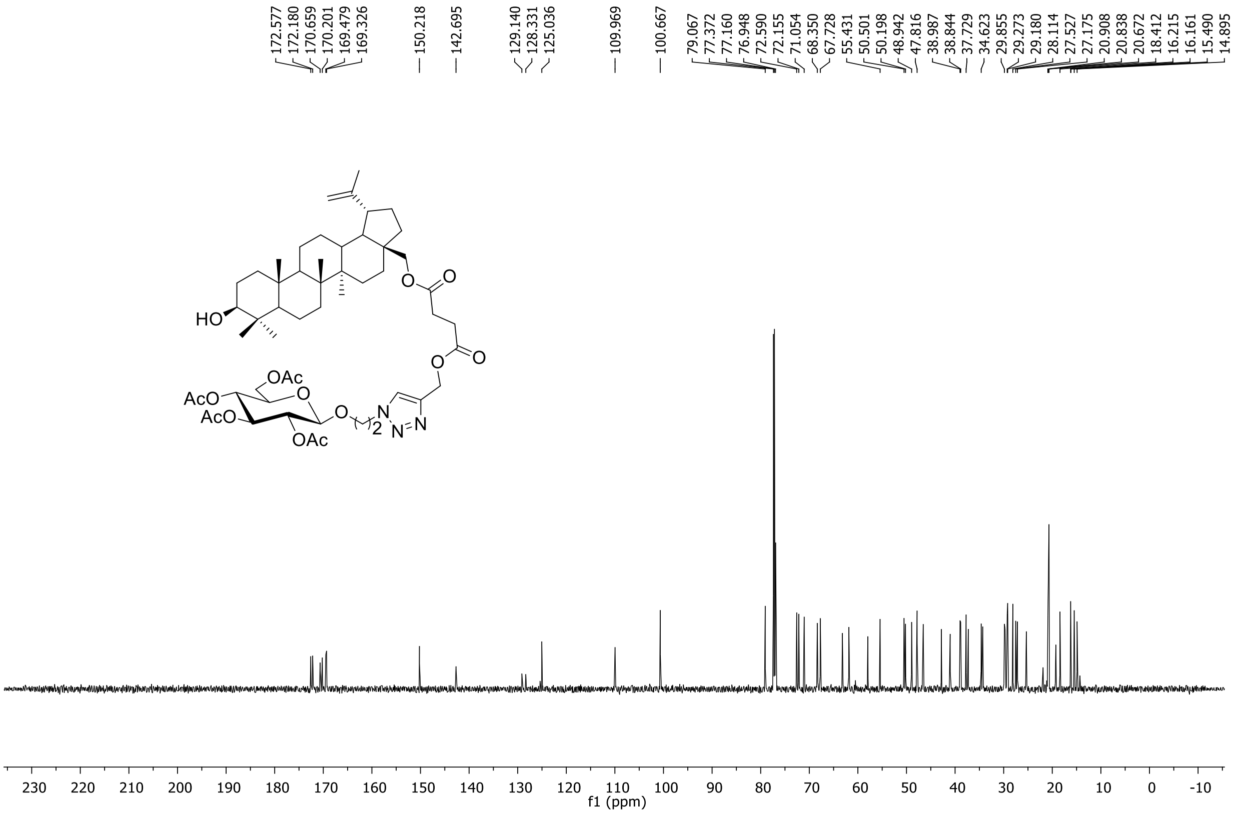

^{13}C-NMR spectrum of (**7c**); 150 MHz/$CDCl_3$/TMS; δ (ppm).

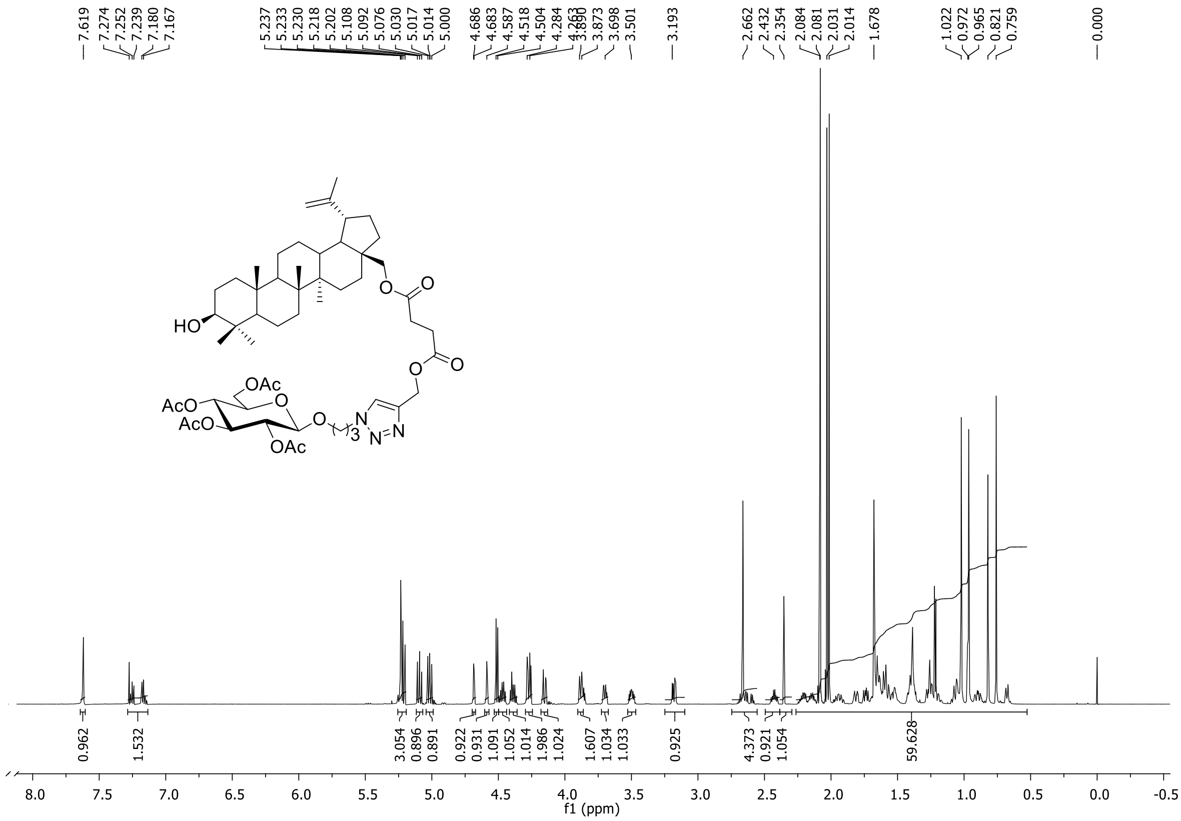

1H-NMR spectrum of (**7d**); 600 MHz/CDCl3/TMS; δ (ppm).

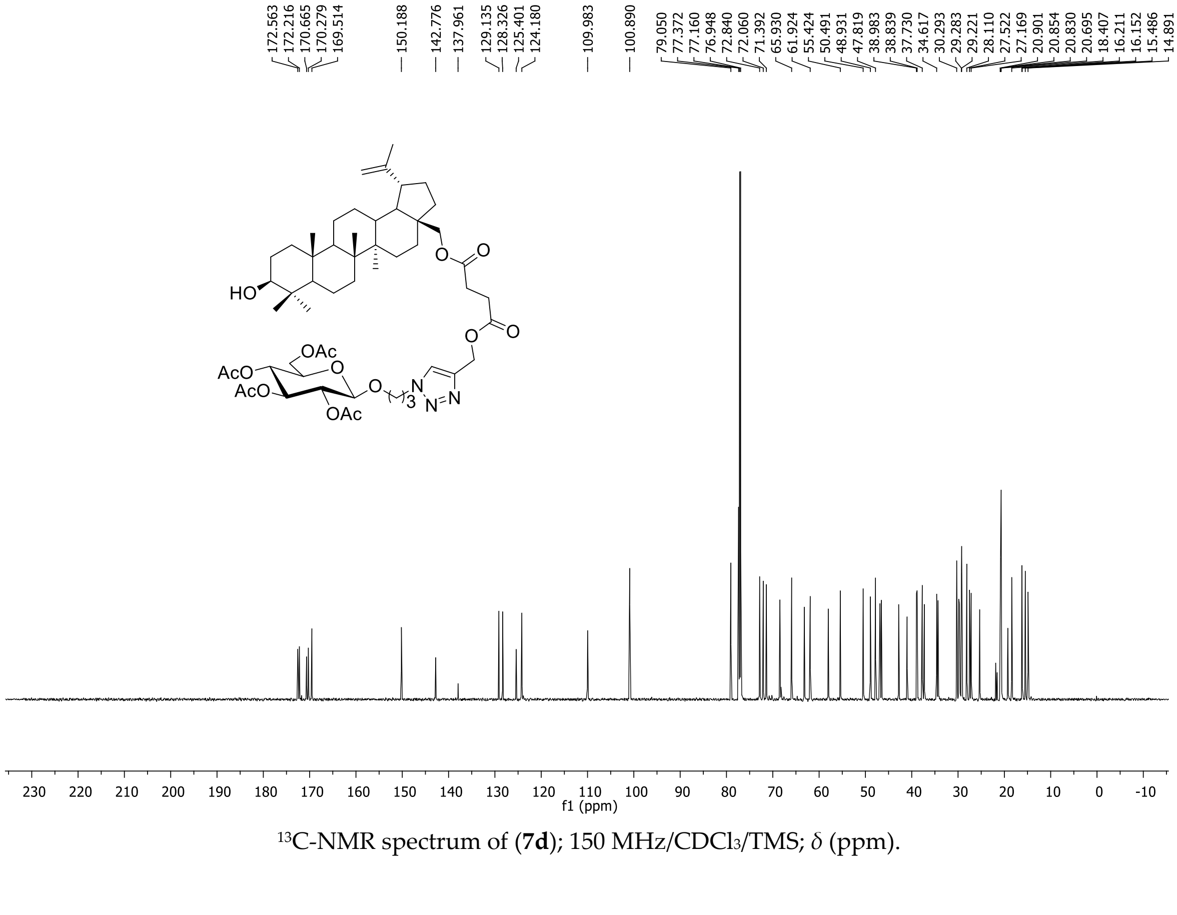

^{13}C-NMR spectrum of (**7d**); 150 MHz/CDCl3/TMS; δ (ppm).

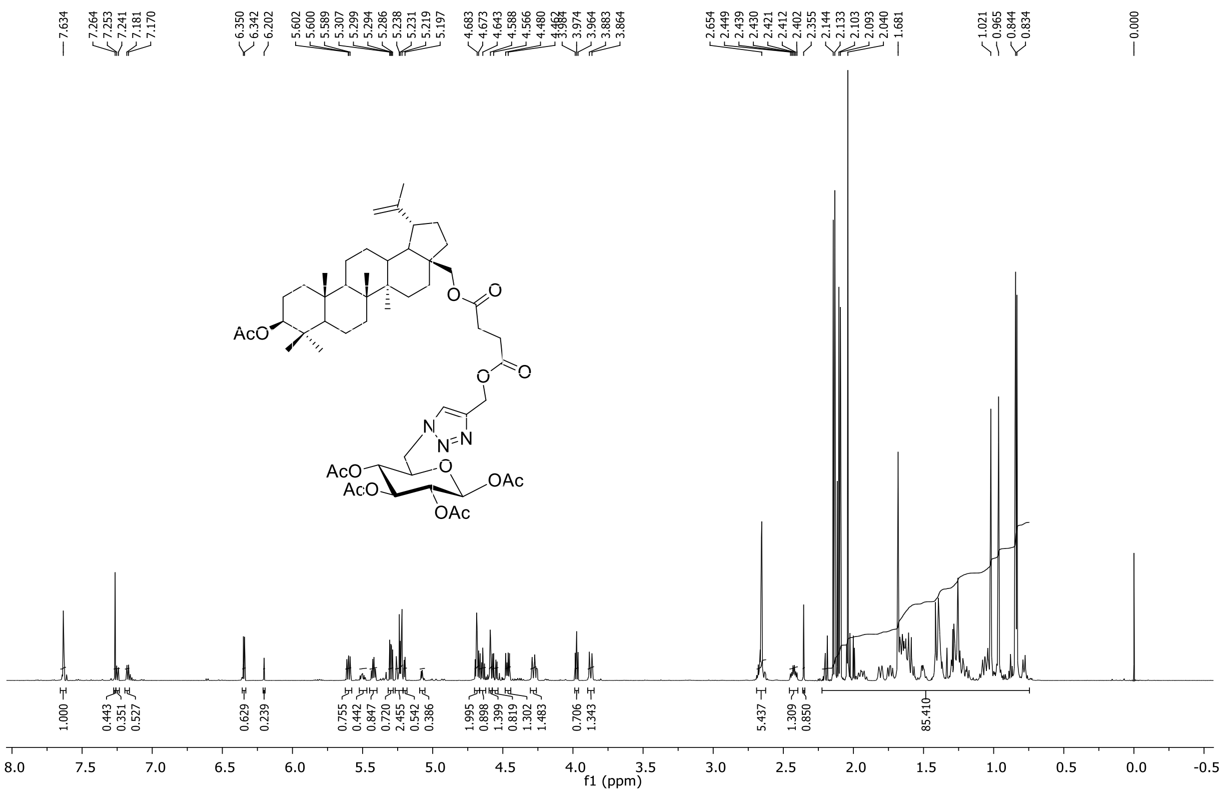

^{1}H-NMR spectrum of (**8a**); 600 MHz/CDCl3/TMS; δ (ppm).

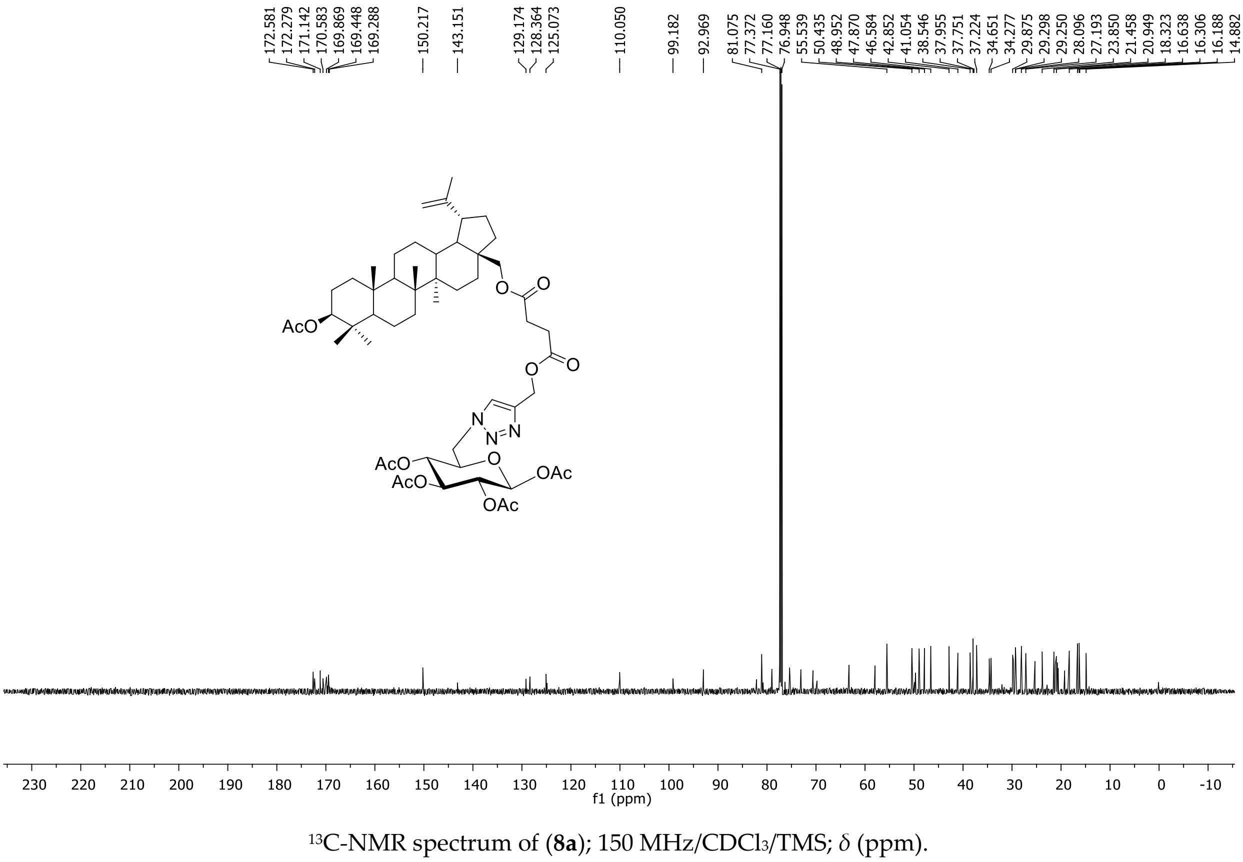

^{13}C-NMR spectrum of (**8a**); 150 MHz/CDCl3/TMS; δ (ppm).

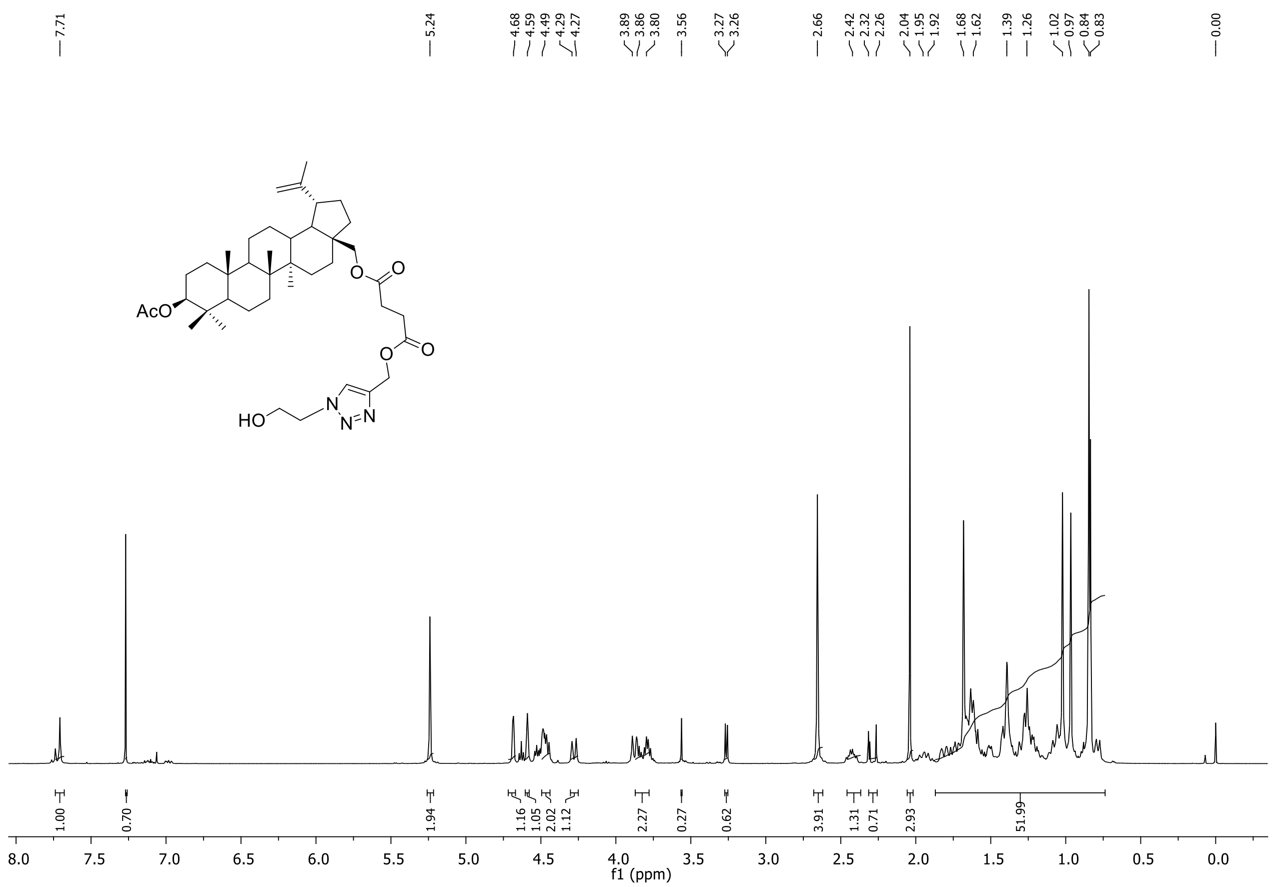

^{1}H-NMR spectrum of (**9a**); 400 MHz/$CDCl_3$/TMS; δ (ppm).

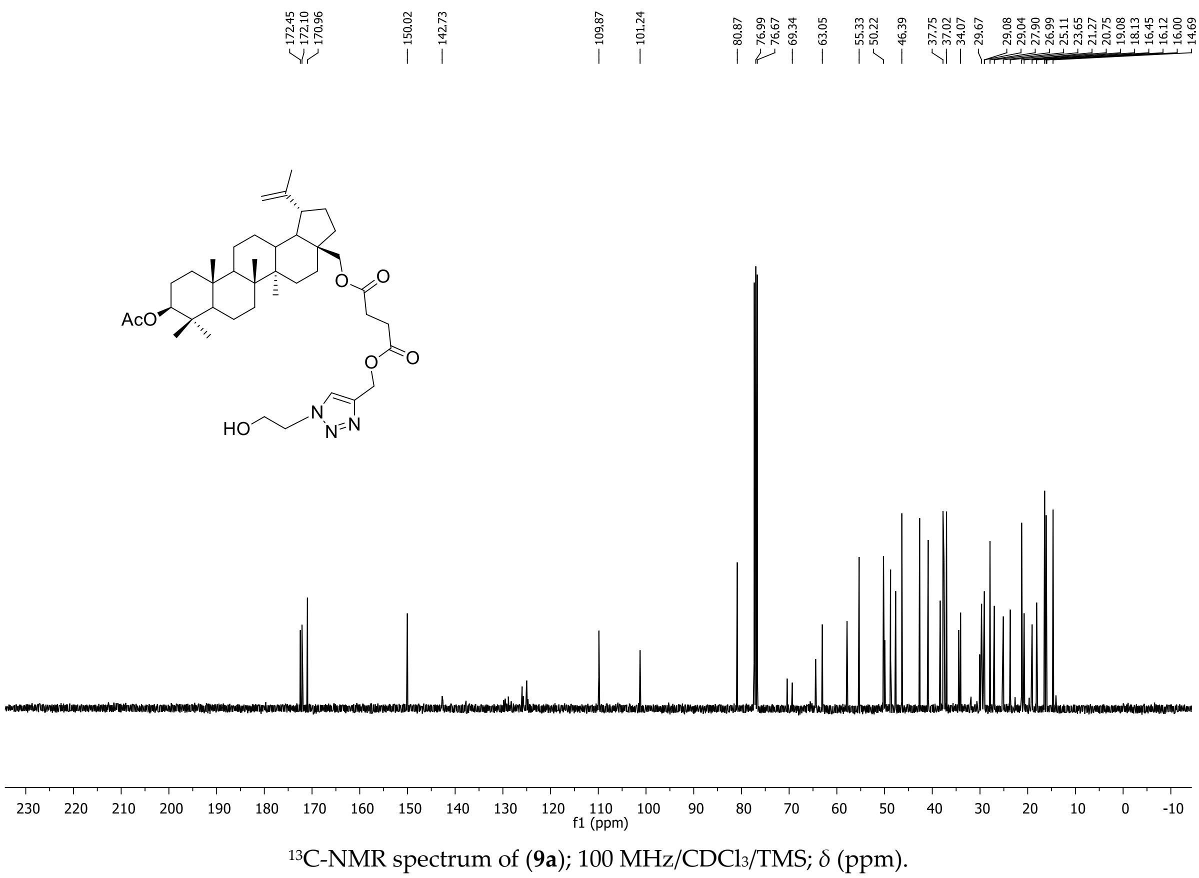

^{13}C-NMR spectrum of (**9a**); 100 MHz/$CDCl_3$/TMS; δ (ppm).

4. References

1. Thibeault, D.; Gauthier, C.; Legault, J.; Bouchard, J.; Dufour, P.; Pichette, A. Synthesis and Structure–Activity Relationship Study of Cytotoxic Germanicane- and Lupane-Type 3β-O-Monodesmosidic Saponins Starting from Betulin. *Bioorg. Med. Chem.* **2007**, *15*, 6144–6157, doi:10.1016/j.bmc.2007.06.033.
2. Grymel, M.; Lalik, A.; Kazek-Kęsik, A.; Szewczyk, M.; Grabiec, P.; Erfurt, K. Design, Synthesis and Preliminary Evaluation of the Cytotoxicity and Antibacterial Activity of Novel Triphenylphosphonium Derivatives of Betulin. *Molecules* **2022**, *27*, 5156, doi:10.3390/molecules27165156.
3. Le Roux, A.; Meunier, S.; Le Gall, T.; Denis, J.; Bischoff, P.; Wagner, A. Synthesis and Radioprotective Properties of Pulvinic Acid Derivatives. *ChemMedChem* **2011**, *6*, 561–569, doi:10.1002/cmdc.201000391.
4. Ibatullin, F.M.; Shabalin, K.A. A Simple and Convenient Synthesis of Glycosyl Azides. *Synth. Commun.* **2000**, *30*, 2819–2823, doi:10.1080/00397910008086908.
5. Brzuska, G.; Pastuch-Gawolek, G.; Krawczyk, M.; Szewczyk, B.; Krol, E. Anti-Tick-Borne Encephalitis Virus Activity of Novel Uridine Glycoconjugates Containing Amide or/and 1,2,3-Triazole Moiety in the Linker Structure. *Pharmaceuticals* **2020**, *13*, 460, doi:10.3390/ph13120460.
6. Pastuch-Gawołek, G.; Malarz, K.; Mrozek-Wilczkiewicz, A.; Musioł, M.; Serda, M.; Czaplinska, B.; Musiol, R. Small Molecule Glycoconjugates with Anticancer Activity. *Eur. J. Med. Chem.* **2016**, *112*, 130–144, doi:10.1016/j.ejmech.2016.01.061.
7. Joosten, J.A.F.; Loimaranta, V.; Appeldoorn, C.C.M.; Haataja, S.; El Maate, F.A.; Liskamp, R.M.J.; Finne, J.; Pieters, R.J. Inhibition of *Streptococcus s Uis* Adhesion by Dendritic Galabiose Compounds at Low Nanomolar Concentration. *J. Med. Chem.* **2004**, *47*, 6499–6508, doi:10.1021/jm049476+.
8. Domińska, M.; Pastuch-Gawołek, G.; Skonieczna, M.; Szeja, W.; Domiński, A.; Kurcok, P. Glycoconjugation of Quinoline Derivatives Using the C-6 Position in Sugars as a Strategy for Improving the Selectivity and Cytotoxicity of Functionalized Compounds. *Molecules* **2022**, *27*, 6918, doi:10.3390/molecules27206918.
